# On the use of ^3^*J*-coupling NMR data to derive structural information on proteins

**DOI:** 10.1007/s10858-020-00355-5

**Published:** 2021-01-25

**Authors:** Lorna J. Smith, Wilfred F. van Gunsteren, Bartosz Stankiewicz, Niels Hansen

**Affiliations:** 1 Department of Chemistry, Inorganic Chemistry Laboratory, University of Oxford, South Parks Road, Oxford, OX1 3QR UK; 2 Laboratory of Physical Chemistry, Swiss Federal Institute of Technology, ETH, 8093 Zurich, Switzerland; 3 Institute of Thermodynamics and Thermal Process Engineering, University of Stuttgart, 70569 Stuttgart, Germany

**Keywords:** Structure refinement, Nuclear magnetic resonance, ^3^*J*-couplings, Averaging time, Restraining force, Conformational sampling, Local-elevation sampling

## Abstract

Values of ^3^*J*-couplings as obtained from NMR experiments on proteins cannot easily be used to determine protein structure due to the difficulty of accounting for the high sensitivity of intermediate ^3^*J*-coupling values (4–8 Hz) to the averaging period that must cover the conformational variability of the torsional angle related to the ^3^*J*-coupling, and due to the difficulty of handling the multiple-valued character of the inverse Karplus relation between torsional angle and ^3^*J*-coupling. Both problems can be solved by using ^3^*J*-coupling time-averaging local-elevation restraining MD simulation. Application to the protein hen egg white lysozyme using 213 backbone and side-chain ^3^*J*-coupling restraints shows that a conformational ensemble compatible with the experimental data can be obtained using this technique, and that accounting for averaging and the ability of the algorithm to escape from local minima for the torsional angle induced by the Karplus relation, are essential for a comprehensive use of ^3^*J*-coupling data in protein structure determination.

**Supplementary Information:**

The online version of this article (10.1007/s10858-020-00355-5) contains supplementary material, which is available to authorized users.

## Introduction

Structural information on proteins can be derived from a variety of observable quantities, such as X-ray diffraction intensities, NMR, CD, Raman or infrared spectra to mention a few (van Gunsteren et al. 2016). For proteins in aqueous solution, NMR measurements provide the highest information density, i.e. ratio of the number of independent, measured values of observable quantities for a molecule and the number of independent molecular degrees of freedom. Quantities observable using NMR techniques are ^3^*J*-couplings, chemical shifts, nuclear Overhauser enhancement intensities (NOEs), *S*^2^ order parameters and residual dipolar couplings (RDCs). The measured value of such an observable quantity *Q* is an average 〈*Q*〉_space,time_ of *Q* over the molecules (space) in the test tube and over a time window determined by the particular measurement technique. This means that 〈*Q*〉  constitutes an average over a Boltzmann-weighted set, i.e. a statistical-mechanical ensemble, of configurations $\vec{r}$. The weights are proportional to $\exp(-V(\vec{r})/(k_{B}T)),$ where *V*($\vec{r}$) indicates the energy of a molecular configuration or structure $\vec{r},\;k_B$ is Boltzmann’s constant and *T* is the temperature.

If an observable quantity *Q* is dependent on the molecular configuration $\vec{r}$, one may try to derive an expression or function $Q(\vec{r})$ that approximates the relation between *Q* and $\vec{r},$ which expression may then be used to derive molecular structures that are compatible with measured values of *Q*, i.e.

1 $$\langle Q \rangle =\int Q(\vec{r})\mathrm{exp}(-V(\vec{r})/({k}_{B}T)){\rm d}\vec{r}/\int \mathrm{exp}(-V(\vec{r})/({k}_{B}T)){\rm d}\vec{r},$$

i.e. Boltzmann averages over the high-dimensional molecular configuration space.

When deriving molecular structures $\vec{r}$ from a set of measured values *Q*^*exp*^ of *Q*, the following problems may be met.

1. *Q*^*exp*^ values are subject to uncertainty or error.
2. It is not possible to fully account for averaging over space and time inherent in the experimental measurement: inversion of the averaging operation in Eq. (1) is impossible.
3. For most bio-molecular systems the number of independent *Q*^*exp*^ values available is much smaller than the number of degrees of freedom of the system. This means that the structure determination problem is underdetermined and can only be addressed by using an atomic model, i.e. a function *V(*$\vec{r}$*)* specifying likely structural parameters (e.g. bond lengths and bond angles) of a system. The function *V*($\vec{r}$) may yield low-energy values for configurations that are physically most reasonable. The fewer *Q*^*exp*^ values are available, the larger the influence of the choice of molecular model and interaction function *V*($\vec{r}$) and its (in)accuracy on the generated structures will be.
4. The function *Q*($\vec{r}$) is not known or its accuracy is uncertain or low.
5. The inverse function $\vec{r}$(*Q*) of the function *Q*($\vec{r}$) may not exist, or if it does, it may be multiple-valued, as in the case of the Karplus relation or function (Karplus 1959, 1963) linking a ^3^*J*-coupling (*Q*) to a torsional angle *θ*($\vec{r}$) in a molecule,
  2 $${}^{3}J(\theta ) = a\cos^{2} (\theta ) + b\cos (\theta ) + c,$$
6. The generation or sampling of molecular configurations $\vec{r}$ must be biased, i.e. guided towards those that are (on average) compatible with *Q*^*exp*^. This is particularly challenging when the inverse function $\vec{r}$(*Q*) of the function *Q*($\vec{r}$) is multiple-valued. The inverse function *θ*(^3^*J*) of Eq. (2) is multiple-valued. For a range of ^3^*J*-values there are four different *θ*-values satisfying Eq. (2).

Although ^3^*J*-couplings are relatively easily measurable, their use to derive molecular structure is hampered by all six mentioned problematic aspects of procedures to derive molecular structure from measured *Q*^*exp*^ values: (i) Small values of ^3^*J*-couplings, such as occurring for ^3^*J*_*HNHα*_-couplings in helical structures, are not easily precisely measured; (ii) The averaging of ^3^*J*-couplings may cover very long time periods and is not limited by the rotational tumbling time of the molecule, as for NOEs; (iii) ^3^*J*-couplings can only be measured for particular parts of proteins, backbone ^3^*J*_*HNHα*_-couplings and side-chain ^3^*J*_*HαHβ*_-couplings only address values for the *φ* and *χ*_1_ torsional angles respectively; Although a range of other ^3^*J*-couplings can be measured, particularly with isotopically labelled samples, in general these show a smaller range of values and often have less well defined Karplus relation parameters. They are therefore not so useful for structure determination (Wang and Bax 1996; Perez et al. 2001); (iv) The parameters *a*, *b* and *c* of the Karplus relation Eq. (2) are of empirical nature and their values are commonly derived by matching X-ray diffraction derived crystal structures ($\vec{r}$) to solution NMR measured ^3^*J*-couplings (*Q*) for a set of (protein) molecules (Schmidt et al. 1999; Schmidt 2007). This leads to an uncertainty of 1–2 Hz in Eq. (2) (Dolenc et al. 2010; Steiner et al. 2012); (v) The inverse function *θ*(^3^*J*) of Eq. (2) is up to fourfold multiple-valued.

A few years ago, a procedure has been proposed to effectively handle the multiple-valuedness of *θ*(^3^*J*) by searching for all possible local minima of the biasing restraining function *V*_*k*_^*J,restr*^(*J*_*k*_(*θ*_*k*_($\vec{r}$(*t*))), 〈*J*_*k*_〉_t_; *K*^*Jr*^, *N*_*le*_, *J*_*k*_^0^, Δ*J*^*fb*^*)* that keeps the value of 〈*J*_*k*_〉 of the *k*th ^3^*J*-coupling close to *J*_*k*_^0^ = *J*_*k*_^*exp*^ (Smith et al. 2016). This restraining function has a flat-bottom on the interval [*J*_*k*_^0^ − Δ*J*^*fb*^, *J*_*k*_^0^ + Δ*J*^*fb*^] and is beyond this interval harmonic, see Fig. 1 of Smith et al. (2016) with Δ*Q*^*h*^ = ∞. Time is denoted by *t* and *K*^*Jr*^ is the overall weight or force constant of the penalty or restraining function. The local-elevation parameter *N*_*le*_ defines the number of intervals *N*_*le*_ of the torsional angle *θ* around the *θ*_*i*_^0^-values

3 $$\theta_{i}^{0} \equiv { 2}\pi {i}/N_{le} \quad i = {1},{2}, \ldots ,N_{le}$$

and their widths

4 $$\Delta \theta^{0} \equiv { 2}\pi /N_{le} ,$$

used in the local-elevation search and sampling algorithm (Christen et al. 2007). The restraining function, biquadratic in *J*_*k*_(*θ*_*k*_($\vec{r}$(*t*))) and 〈*J*_*k*_〉_*t*_, only yields a non-zero energy and restraining force when both, *J*_*k*_(*θ*_*k*_($\vec{r}$(*t*))) and 〈*J*_*k*_〉_*t*_, adopt values on the same side outside the flat-bottom region. When either the instantaneous value *J*_*k*_(*θ*_*k*_($\vec{r}$(*t*))) or the average 〈*J*_*k*_〉_*t*_ satisfies *J*^0^ = *J*^*exp*^ within a given uncertainty Δ*J*^*fb*^, there is no need for restraining.

The local-elevation searching and sampling technique (Huber et al. 1994) is used, in which the potential energy at already visited parts of configuration space is raised in order to avoid repetitive sampling of the same parts of configuration space in a simulation. The basic idea of local-elevation structure refinement based on adaptive restraints (Christen et al. 2007) is that whenever the simulated average  〈*J*〉 _*t*_ of the ^3^*J*-coupling and the current value *J*(*t*) of the ^3^*J*-coupling do not match the measured target value *J*^*0*^ = *J*^*exp*^ to within an uncertainty Δ*J*^*fb*^, the force constant or weight ${\omega }_{{\theta }_{ki}}(t)$ of the penalty or restraining function *V*_*k*_^*J,restr*^(*J*_*k*_(*θ*_*k*_($\vec{r}$(*t*)))), 〈*J*_*k*_〉_*t*_;* K*^*Jr*^, *N*_*le*_, *J*_*k*_^*0*^, Δ*J*^*fb*^), acting on the current value *θ*_*k*_(*t*) of the torsional angle *θ*_*k*_ corresponding to the ^3^*J*-coupling, is increased. The restraining potential-energy term of a given ^3^*J*-coupling *J*_*k*_ is a sum of *N*_*le*_ terms, local with respect to a particular value *θ*_*i*_^*0*^ of *θ*_*k*_,

5 $$V_{k}^{J,restr} \left( {\theta_{k} \left( {\vec{r}\left( t \right)} \right);K^{Jr} ,N_{le} ,J_{k}^{0} ,\Delta J^{fb} } \right) = \sum\limits_{i = 1}^{{N_{le} }} {K^{Jr} \omega_{{\theta_{ki} }} (t)\exp \left( { - (\theta_{k} (t) - \theta_{i}^{0} )^{2} /(2(\Delta \theta^{0} )^{2} )} \right)}$$

with the weight ${\omega }_{{\theta }_{ki}}\left(t\right)$ of the *i*th penalty function changing during the simulation according to

6 $$\omega_{{\theta_{ki} }} \left( t \right) = t^{ - 1} \mathop \smallint \limits_{0}^{t} \delta_{{\theta_{k} \left( {\vec{r}\left( {t^{\prime}} \right)} \right)\theta_{i}^{0} }} (J_{k} \left( {t^{\prime}} \right) - J_{k}^{0} - \Delta J^{fb} )^{2} H\left( {J_{k} \left( {t^{\prime}} \right);J_{k}^{0} + \Delta J^{fb} } \right)( \langle J_{k} \rangle_{t^{\prime}} - J_{k}^{0} - \Delta J^{fb} )^{2} H( \langle J_{k} \rangle_{t^{\prime}} ;J_{k}^{0} + \Delta J^{fb} ){\rm d}t^{\prime}$$

for an attractive (both, *J*_*k*_(*t*) and  〈*J*_*k*_〉 _*t*_ larger than *J*_*k*_^*0*^) ^3^*J*-coupling restraint, and according to

7 $$\omega _{{\theta _{{ki}} }} \left( t \right) = t^{{ - 1}} \mathop \smallint \limits_{0}^{t} \delta _{{\theta _{k} \left( {\vec{r}\left( {t^{\prime}} \right)} \right)\theta _{i}^{0} }} (J_{k} \left( {t^{\prime}} \right) - J_{k}^{0} + \Delta J^{{fb}} )^{2} (1 - H\left( {J_{k} \left( {t^{\prime}} \right);J_{k}^{0} - \Delta J^{{fb}} } \right))(\langle J_{k} \rangle _{{t^{\prime}}} - J_{k}^{0} + \Delta J^{{fb}} )^{2} (1 - H(\langle J_{k} \rangle _{{t^{\prime}}} ;J_{k}^{0} - \Delta J^{{fb}} )){\rm d}t^{\prime}$$

for a repulsive (both, *J*_*k*_(*t*) and  〈*J*_*k*_〉 _*t*_ smaller than *J*_*k*_^*0*^) ^3^*J*-coupling restraint. The Kronecker delta δ is defined using finite differences,

8 $$\begin{aligned} \delta_{{\theta_{k} \left( {\vec{r}\left( {t^{\prime}} \right)} \right)\theta_{i}^{0} }} &= 1\quad {\text{ if }}\left( {\theta_{i}^{0} - \Delta \, \theta_{i}^{0} /{2}} \right) \, \le \theta_{i} (t) < \left( {\theta_{i}^{0} + \Delta \, \theta_{i}^{0} /{2}} \right) \hfill \\ \delta_{{\theta_{k} \left( {\vec{r}\left( {t^{\prime}} \right)} \right)\theta_{i}^{0} }} &= 0\quad {\text{ otherwise}} \hfill \\ \end{aligned}$$

and the Heaviside step function *H*(*x*;*x*_*0*_) is defined by

9 $$\begin{aligned} H\left( {x;x_{0} } \right) & = 0{\text{ for }}x < x_{0} \\ & {\text{ = 1 for }}x \ge x_{0} . \\ \end{aligned}$$

This means that the torsional angle *θ* is pushed away from the value *θ*(*t*) when both the current *J*(*t*) and the averaged  〈*J*〉 _*t*_ values are deviating more than ± Δ*J*^*fb*^ from the target value *J*^*0*^. In this way the whole range of *θ* values is searched for those values that yield ^3^*J*-couplings close to *J*^*exp*^ (Allison and van Gunsteren 2009). The time-averaged value  〈*J*〉 _*t*_ of *J*(*t*) is commonly (Torda et al. 1989) exponentially damped

10 $$\langle {J}_{k}({\theta }_{k}(\vec{r}(t))){\rangle}_{t}={\left[{\tau }_{J}(1-\text{exp}(-t/{\tau }_{J}))\right]}^{-1}{\int }_{0}^{t}\text{exp}(-(t-t{^{\prime}})/{\tau }_{J}){J}_{k}({\theta }_{k}(\vec{r}(t{^{\prime}}))){\rm d}t{^{\prime}}$$

in the restraining function *V*_*k*_^*J,restr*^(*J*_*k*_*(θ*_*k*_*(*$\vec{r}$(*t*))), 〈*J*_*k*_〉_*t*_; *K*^*Jr*^, *N*_*le*_, *J*_*k*_^*0*^, Δ*J*^*fb*^*)*, Eqs. (6) and (7), in order to avoid that the restraining force progressively approaches zero with time.

This procedure to refine protein structure using measured ^3^*J*-couplings was applied (Smith et al. 2016) to sets of 95 backbone ^3^*J*_*HNHα*_-couplings and 62 side-chain ^3^*J*_*HαHβ*_-couplings (58 stereo-specifically assigned ^3^*J*_*HαHβ*_-couplings plus those of Glu 7 and Arg 45 were included in the set of restraints) measured for hen egg white lysozyme (HEWL) (Smith et al. 1991). It was concluded that (i) the weight or force constant *K*^*J,restr*^ of the restraining function should be chosen as small as possible while large enough to bring the average ^3^*J*-couplings close to the target values in order to approximate as good as possible the properly (using *V*($\vec{r}$)) Boltzmann-weighted conformational probability distribution, (ii) the averaging time *τ*_*J*_ should match the experimental one but should not be larger than 1/10 of the total simulation time in order to secure sufficient statistics when averaging, and (iii) the flat-bottom Δ^3^*J*^*fb*^ should represent the uncertainty or inaccuracy of the Karplus relation ^3^*J(θ)*.

In the present article the biquadratic time-averaging local-elevation restraining (BQ-TA-LER) method (Smith et al. 2016) is further investigated using NMR and X-ray data on HEWL, particularly concentrating on side chains whose ^3^*J*_*HαHβ*_-couplings are significantly averaged.

**Fig. 1:**
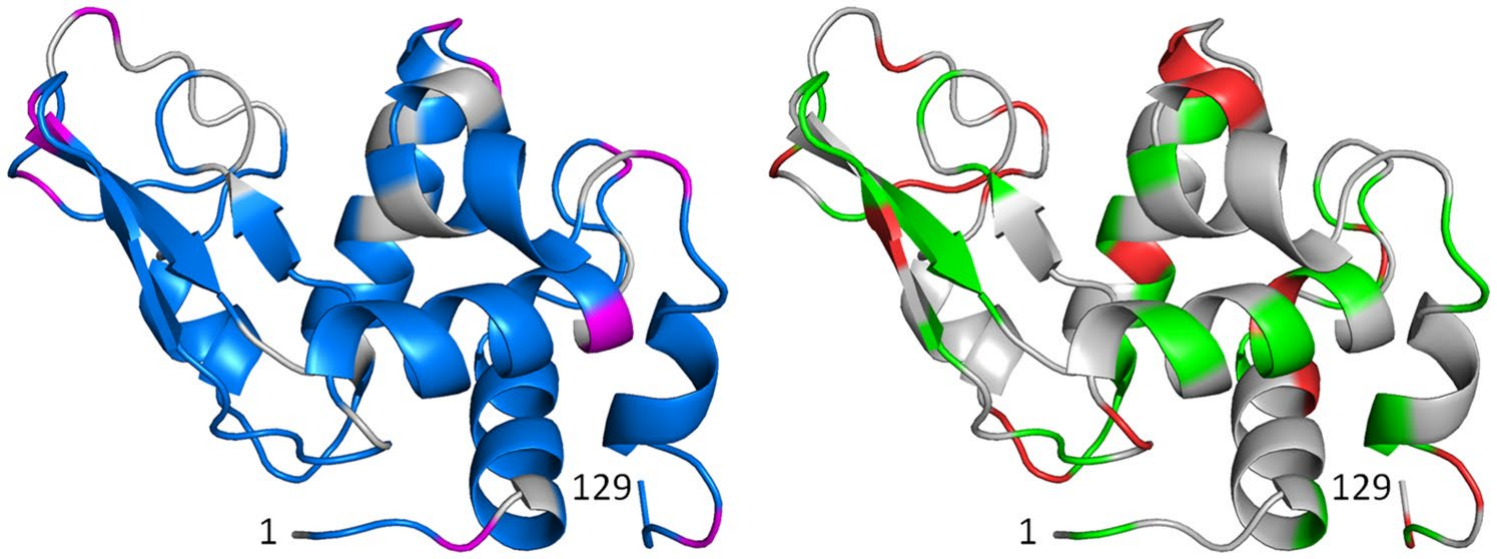
Ribbon pictures of the structure of HEWL with residues for which experimentally derived backbone ^3^*J*_*HNHα*_-coupling values (left panel, experimentally stereo-specifically assigned (set *bb1*) in blue, computationally stereo-specifically assigned (set *bb2*) in magenta) and side-chain ^3^*J*_*Hα-Hβ*_-coupling values (right panel, experimentally stereo-specifically assigned (set *sc1*) in green, computationally stereo-specifically assigned (set *sc2*) in red) are available

## Materials and methods

Energy minimisations and molecular dynamics simulations were performed using the GROMOS bio-molecular simulation software (Schmid et al. 2011a, 2012; van Gunsteren et al. 2019).

### Molecular model

The protein was modelled using the GROMOS bio-molecular force field 54A7 (Poger et al. 2010; Schmid et al. 2011b). In view of the pH used in the experimental NMR measurements, pH = 3.5, only Glu 35 was protonated and His was doubly protonated (Bartik et al. 1994). The simple point charge (SPC) model (Berendsen et al. 1981) was used to describe the solvent molecules in the rectangular periodic box. To compensate for the overall positive charge of the protein, 10 Cl^−^ ions were included in the solution. All bond lengths and the bond angle of the water molecules were kept rigid with a relative geometric precision of 10^–4^ using the SHAKE algorithm (Ryckaert et al. 1977), allowing for a 2 fs MD time step in the leap-frog algorithm (Hockney and Eastwood 1981) used to integrate the equations of motion. For the non-bonded interactions a triple-range method (van Gunsteren et al. 1986) with cut-off radii of 0.8/1.4 nm was used. Short-range (within 0.8 nm) van der Waals and electrostatic interactions were evaluated every time step based on a charge-group pair list (van Gunsteren et al. 2019). Medium-range van der Waals and electrostatic interactions, between pairs at a distance larger than 0.8 nm and shorter than 1.4 nm, were evaluated every fifth time step (10 fs), at which time point the pair list was updated, and kept constant between updates. Outside the larger cut-off radius (1.4 nm) a reaction-field approximation (Barker and Watts 1973; Tironi et al. 1995) with a relative dielectric permittivity of 61 (Heinz et al. 2001) was used. Minimum-image periodic boundary conditions were applied.

### Simulation set-up

Four X-ray crystal structures were used as initial structures for the energy minimisations followed by MD simulations.

1. Structure “2VB1” of the Protein Data Bank (PDB) (Berman et al. 2000), derived from a triclinic unit cell at 0.065 nm resolution at *T* = 100 K. It contains multiple side-chain conformations for 46 residues.
2. Structure “4LZT” of the PDB, derived from a triclinic unit cell at 0.095 nm resolution at *T* = 295 K. It contains multiple side-chain conformations for 8 residues.
3. Structure “1IEE” of the PDB, derived from a tetragonal unit cell at 0.094 nm resolution at *T* = 110 K. It contains multiple side-chain conformations for 33 residues.
4. Structure “1AKI” of the PDB, derived from an orthorhombic unit cell at 0.15 nm resolution at *T* = 298 K. It contains no multiple side-chain conformations.

For the initial structures the side-chain conformation with the highest occupancy was chosen. The atom-positional root-mean-square differences (RMSD) between these four initial structures are for *2VB1/4LZT* 0.086 nm for all atoms and 0.027 nm for the backbone atoms, for *2VB1/1IEE* 0.191 nm (all) and 0.075 nm (bb), for *2VB1/1AKI* 0.165 nm (all) and 0.056 nm (bb), for *4LZT/1IEE* 0.183 nm (all) and 0.073 nm (bb), for *4LZT/1AKI* 0.158 nm (all) and 0.049 nm (bb), and for *1IEE/1AKI* 0.151 nm (all) and 0.047 nm (bb).

An initial structure was first energy minimised in vacuo to release possible strain induced by small differences in bond lengths, bond angles, improper dihedral angles, and short distance non-bonded contacts between the force-field parameters and the X-ray structure. Subsequently, the protein was put into a rectangular box filled with water molecules, such that the minimum solute-wall distance was 1.0 nm, and water molecules closer than 0.23 nm from the solute were removed. This resulted in boxes with 12,157 water molecules for the initial protein structures. In order to relax unfavourable contacts between atoms of the solute and the solvent, a second energy minimisation was performed for the protein in the periodic box with water while keeping the atoms of the solute harmonically position-restrained (van Gunsteren et al. 2019) with a force constant of 25,000 kJ mol^−1^ nm^−2^.

The resulting protein-water configuration was used as initial configuration for the MD simulation. In order to avoid artificial deformations in the protein structure due to relatively high-energy atomic interactions still present in the system, the MD simulation was started at *T* = 60 K and then the temperature was slowly raised to *T* = 308 K. Initial atomic velocities were sampled from a Maxwell distribution at *T* = 60 K. The equilibration scheme consisted of five short 20 ps simulations at temperatures 60, 120, 180, 240 and 308 K at constant volume. During the first four of the equilibration periods, the solute atoms were harmonically restrained to their positions in the initial structures with force constants of 25,000, 2500, 250, and 25 kJ mol^−1^ nm^−2^. The temperature was kept constant using the weak-coupling algorithm (Berendsen et al. 1984) with a relaxation or coupling time *τ*_*Τ*_ = 0.1 ps. Solute and solvent were separately coupled to the heat bath. Following this equilibration procedure, the simulations were performed at a reference temperature of 308 K and a reference pressure of 1 atm. The pressure was kept constant using the weak-coupling algorithm (Berendsen et al. 1984) with a coupling time *τ*_*p*_ = 0.5 ps and an isothermal compressibility *κ*_*T*_ = 4.575 × 10^–4^ (kJ mol^−1^ nm^−3^)^−1^. The centre of mass motion of the system was removed every 1000 time steps (2 ps).

### ^3^*J*-coupling restraining

Two sets of backbone ^3^*J*_*HN-Hα*_ couplings and two sets of side-chain ^3^*J*_*Hα-Hβ*_ couplings for restraining (Smith et al. 2016) were used, see Tables 1, 2, 3 and 4.

1. A set (*bb1*) of 95 backbone ^3^*J*_*HN-Hα*_-coupling values, see Table II of Smith et al. (1991) from which the values for 11 glycine residues were omitted, because these had not been stereo-specifically assigned.
2. A set (*bb2*) of 22 experimentally stereo-specifically unassigned backbone ^3^*J*_*HN-Hα*_-coupling values for the 11 glycine residues, see Table II of Smith et al. (1991). 10 of these were stereo-specifically assigned based on the ^3^*J*_*HN-Hα*_-coupling values calculated from the four unrestrained MD simulations starting from the four mentioned X-ray structures (see below) as either 4 or 3 of the unrestrained MD simulations suggested the same stereo-specific assignment. Gly 104 could not be stereo-specifically assigned using this criterion. Instead it was stereo-specifically assigned based on the ^3^*J*_*HN-Hα*_-coupling values calculated for the four X-ray structures.
3. A set (*sc1*) of 58 ^3^*J*_*Hα-Hβ*_-coupling values, see Tables III and IV of Smith et al. (1991), which were stereo-specifically assigned using experimental data.
4. A set (*sc2*) of 38 out of 40 experimentally stereo-specifically unassigned ^3^*J*_*Hα-Hβ*_-coupling values, see Table III of Smith et al. (1991), which were stereo-specifically assigned based on the ^3^*J*_*Hα-Hβ*_-coupling values calculated from the four unrestrained MD simulations starting from the four mentioned X-ray structures (see below) as either 4 or 3 of the unrestrained MD simulations suggested the same stereo-specific assignment. Glu 7 could not be stereo-specifically assigned using this criterion. It could also not be stereo-specifically assigned using the ^3^*J*_*Hα-Hβ*_-coupling values calculated for the four X-ray structures.

**Table 1:** Backbone ^3^*J*_*HNHα*_-coupling values (95) in Hz derived and assigned based on NMR measurements (set *bb1*) and from four unrestrained MD simulations starting from four X-ray crystal structures, and the mean of the latter four values and the root-mean-square deviation (RMSD) from it

Residue	Experimental value	MD simulation (X-ray crystal)					
		*2VB1*	*4LZT*	*1IEE*	*1AKI*	Mean	RMSD
Val 2	10.0 (9.7)	7.9 (9.6)	7.8 (9.4)	8.3 (8.7)	*7.3* (9.1)	7.8 (9.2)	0.4 (0.3)
Phe 3	7.4	7.5 (6.1)	7.0 (5.7)	6.4 (*5.2*)	7.4 (6.1)	7.1 (5.8)	0.4 (0.4)
Cys 6	5.8	5.5 (5.8)	5.7 (5.7)	6.3 (5.0)	5.5 (5.5)	5.8 (5.5)	0.3 (0.3)
Glu 7	4.5	4.3 (4.6)	4.2 (4.8)	4.1 (3.6)	4.3 (4.9)	4.2 (4.5)	0.1 (0.5)
Leu 8	5.5	4.7 (5.1)	4.7 (4.9)	4.6 (5.0)	4.6 (4.5)	4.7 (4.9)	0.1 (0.2)
Ala 9	3.7	4.2 (3.7)	4.2 (3.8)	4.2 (3.6)	4.2 (2.8)	4.2 (3.5)	0.0 (0.4)
Ala 10	3.9	4.5 (4.8)	4.5 (4.8)	4.6 (4.3)	4.5 (4.9)	4.5 (4.7)	0.0 (0.2)
Ala 11	4.8	4.5 (4.7)	4.6 (4.9)	4.5 (5.3)	4.7 (4.4)	4.6 (4.8)	0.1 (0.3)
Met 12	4.6	4.9 (4.5)	4.5 (5.0)	4.6 (4.3)	4.6 (4.4)	4.7 (4.6)	0.2 (0.3)
Lys 13	4.2	4.7 (4.8)	4.4 (4.8)	4.6 (4.7)	4.7 (4.4)	4.6 (4.7)	0.1 (0.2)
Arg 14	4.4	4.6 (4.1)	4.8 (4.4)	4.7 (4.4)	4.5 (4.6)	4.7 (4.4)	0.1 (0.2)
His 15	9.2	7.4 (7.6)	7.4 (8.3)	7.6 (7.3)	7.4 (8.1)	7.5 (7.8)	0.1 (0.4)
Leu 17	7.6	7.0 (7.5)	7.4 (6.8)	7.4 (7.4)	7.4 (6.6)	7.3 (7.1)	0.2 (0.4)
Asp 18	5.7	6.0 (5.3)	7.0 (5.1)	6.8 (4.5)	6.2 (4.2)	6.5 (4.8)	0.4 (0.5)
Asn 19	7.0	6.4 (6.6)	5.7 (6.7)	6.3 (6.9)	6.7 (6.6)	6.3 (6.7)	0.4 (0.1)
Tyr 20	5.5	7.0 (5.0)	6.5 (5.3)	6.7 (4.7)	5.0 (4.7)	6.3 (4.9)	0.8 (0.3)
Arg 21	6.8	6.0 (6.7)	6.3 (6.8)	5.6 (6.7)	6.6 (6.9)	6.1 (6.8)	0.4 (0.1)
Tyr 23	8.6	7.8 (9.5)	7.1 (9.2)	8.2 (8.8)	7.3 (9.6)	7.6 (9.3)	0.4 (0.3)
Asn 27	5.4	4.2 (4.0)	4.5 (4.1)	4.1 (4.0)	4.3 (3.7)	4.3 (4.0)	0.1 (0.2)
Trp 28	6.0	5.2 (5.2)	5.0 (5.4)	5.2 (5.4)	5.1 (4.6)	5.1 (5.2)	0.1 (0.3)
Val 29	5.9	4.8 (4.9)	4.8 (4.8)	4.8 (4.8)	4.9 (4.2)	4.8 (4.7)	0.0 (0.3)
Cys 30	3.8	4.6 (4.0)	4.4 (4.3)	4.6 (4.0)	4.9 (3.6)	4.6 (4.0)	0.2 (0.2)
Ala 31	3.8	4.4 (4.2)	4.2 (4.2)	4.3 (4.2)	4.6 (4.1)	4.4 (4.2)	0.1 (0.0)
Ala 32	4.8	4.5 (4.7)	4.3 (4.7)	4.6 (4.5)	4.8 (3.5)	4.6 (4.4)	0.2 (0.5)
Lys 33	3.6	4.6 (3.6)	5.2 (3.9)	4.6 (3.8)	*5.7* (4.1)	5.0 (3.9)	0.5 (0.2)
Phe 34	7.6	5.6 (7.7)	*5.2* (7.6)	6.1 (7.5)	*5.3* (7.4)	5.6 (7.6)	0.4 (0.1)
Glu 35	7.2	7.7 (5.6)	6.9 (6.2)	7.8 (6.6)	7.1 (6.2)	7.4 (6.2)	0.4 (0.4)
Ser 36	9.6	7.9 (9.4)	8.5 (9.4)	7.6 (9.4)	*5.7* (9.7)	*7.4* (9.5)	1.0 (0.1)
Phe 38	6.3	6.8 (6.8)	6.8 (6.8)	6.8 (6.8)	6.8 (6.8)	6.8 (6.8)	0.0 (0.0)
Asn 39	8.8	8.1 (8.6)	7.9 (8.9)	7.2 (8.8)	7.6 (9.5)	7.7 (9.0)	0.3 (0.3)
Thr 40	5.4	4.7 (4.7)	4.7 (4.8)	4.7 (4.3)	4.5 (4.6)	4.7 (4.6)	0.1 (0.2)
Gln 41	9.2	*6.5* (8.7)	*6.7* (8.6)	*6.7* (7.7)	*6.9* (9.1)	*6.7* (8.5)	0.1 (0.5)
Ala 42	4.5	5.6 (4.0)	6.0 (4.4)	5.6 (4.5)	5.6 (4.4)	5.7 (4.3)	0.2 (0.2)
Thr 43	9.3	8.7 (9.2)	8.8 (9.0)	8.9 (9.2)	8.8 (9.2)	8.8 (9.1)	0.1 (0.1)
Asn 44	9.4	7.7 (9.2)	8.1 (9.5)	7.8 (8.3)	7.7 (8.8)	7.8 (8.9)	0.2 (0.5)
Arg 45	7.7	8.1 (8.4)	7.8 (8.2)	7.7 (6.3)	7.6 (8.2)	7.8 (7.8)	0.2 (0.9)
Asn 46	8.8	*6.1* (7.5)	7.9 (7.9)	8.1 (8.1)	7.3 (9.6)	7.4 (8.3)	0.8 (0.8)
Thr 47	4.4	4.2 (6.2)	4.3 (6.1)	4.3 (4.1)	4.4 (4.3)	4.3 (5.2)	0.1 (1.0)
Asp 48	7.7	6.3 (6.8)	5.7 (7.3)	5.7 (6.5)	6.1 (6.2)	6.0 (6.7)	0.3 (0.4)
Ser 50	7.8	*5.3* (8.0)	5.8 (8.3)	5.9 (6.2)	*5.7* (6.5)	*5.7* (7.3)	0.2 (0.9)
Thr 51	9.8 (9.7)	8.3 (9.0)	9.2 (9.2)	9.2 (9.3)	8.9 (9.6)	8.9 (9.3)	0.4 (0.2)
Asp 52	9.6	8.0 (9.0)	7.6 (9.0)	*7.4* (9.1)	7.6 (8.4)	7.7 (8.9)	0.2 (0.3)
Tyr 53	9.6	8.9 (9.7)	8.5 (9.7)	8.7 (9.5)	8.1 (9.7)	8.6 (9.7)	0.3 (0.1)
Leu 56	9.7	*7.3* (9.5)	8.3 (9.5)	7.7 (9.7)	8.0 (9.4)	7.8 (9.5)	0.4 (0.1)
Gln 57	6.3	6.8 (6.7)	6.6 (6.7)	6.8 (6.7)	6.7 (6.4)	6.7 (6.6)	0.1 (0.1)
Ile 58	8.0	8.0 (7.2)	8.1 (7.1)	7.9 (8.1)	7.9 (7.4)	8.0 (7.4)	0.1 (0.4)
Ser 60	5.1	5.2 (7.1)	5.0 (6.9)	5.1 (*8.3*)	4.9 (5.9)	5.1 (7.1)	0.0 (0.9)
Arg 61	6.2	5.7 (6.7)	5.6 (6.2)	5.9 (7.8)	6.2 (5.3)	5.9 (6.5)	0.2 (0.9)
Cys 64	8.8	7.5 (9.4)	8.4 (9.2)	7.6 (9.3)	9.1 (9.4)	8.2 (9.3)	0.7 (0.1)
Asn 65	9.4	*6.3* (8.8)	*6.5* (8.9)	*6.0* (9.0)	*5.9* (9.0)	*6.2* (8.9)	0.2 (0.1)
Asp 66	10.0 (9.7)	8.1 (9.6)	8.5 (9.6)	8.3 (9.7)	8.4 (9.6)	8.3 (9.6)	0.1 (0.0)
Arg 68	9.7	*6.9* (9.6)	*6.8* (9.7)	*6.7* (9.2)	*6.7* (9.4)	*6.8* (9.5)	0.1 (0.2)
Thr 69	9.3	*6.0* (9.5)	*5.8* (8.9)	*5.6* (9.6)	*6.3* (9.6)	*5.9* (9.4)	0.3 (0.3)
Cys 76	8.8	7.4 (7.2)	7.8 (7.1)	7.1 (6.8)	7.1 (7.9)	7.4 (7.3)	0.3 (0.4)
Asn 77	7.4	6.8 (6.9)	6.8 (6.9)	6.8 (6.6)	6.7 (6.9)	6.8 (6.8)	0.0 (0.1)
Ile 78	8.0	*4.7* (8.3)	*4.6* (8.2)	*5.1* (6.7)	*5.8* (8.9)	*5.1* (8.0)	0.5 (0.8)
Cys 80	3.6	4.5 (4.5)	4.5 (4.4)	4.5 (4.0)	4.4 (4.1)	4.5 (4.2)	0.0 (0.2)
Ser 81	3.6	4.0 (4.1)	3.9 (4.2)	3.9 (4.3)	3.9 (3.3)	3.9 (4.0)	0.0 (0.4)
Ala 82	5.4	4.7 (4.7)	4.7 (4.6)	4.8 (4.7)	4.8 (5.1)	4.8 (4.8)	0.1 (0.2)
Leu 83	7.2	5.3 (6.4)	*5.1* (6.9)	5.3 (7.2)	5.2 (6.6)	5.2 (6.8)	0.1 (0.3)
Leu 84	9.2	*5.6* (9.4)	*6.2* (9.4)	*5.9* (9.2)	*6.0* (8.9)	*5.9* (9.2)	0.2 (0.2)
Ser 85	5.8	6.3 (5.2)	5.7 (6.0)	6.6 (4.2)	5.9 (5.0)	6.1 (5.1)	0.3 (0.6)
Ser 86	5.8	4.9 (5.8)	4.6 (5.9)	4.8 (4.3)	4.9 (5.7)	4.8 (5.4)	0.1 (0.7)
Asp 87	8.9	*6.5* (*6.4*)	7.9 (7.0)	*6.8* (7.2)	7.5 (7.2)	7.2 (7.0)	0.6 (0.3)
Ile 88	6.5	7.3 (8.0)	6.8 (7.8)	6.6 (7.1)	7.4 (7.0)	7.0 (7.5)	0.3 (0.5)
Ala 90	4.2	4.5 (5.2)	4.8 (4.9)	4.5 (4.9)	4.5 (3.3)	4.6 (4.6)	0.1 (0.7)
Ser 91	5.5	4.2 (4.7)	4.3 (4.6)	4.5 (4.4)	4.3 (4.4)	4.3 (4.5)	0.1 (0.1)
Val 92	5.6	4.8 (4.8)	4.5 (5.0)	5.0 (4.8)	4.8 (4.2)	4.8 (4.7)	0.2 (0.3)
Asn 93	4.4	4.5 (4.7)	4.4 (4.4)	4.5 (4.1)	4.6 (4.0)	4.5 (4.3)	0.1 (0.3)
Cys 94	6.3	5.1 (5.5)	5.6 (5.7)	5.2 (5.6)	5.2 (5.1)	5.3 (5.5)	0.2 (0.2)
Lys 96	4.4	4.3 (4.3)	4.1 (4.6)	4.4 (4.3)	4.2 (4.2)	4.3 (4.4)	0.1 (0.2)
Lys 97	6.5	4.7 (4.5)	5.4 (4.8)	5.1 (4.6)	4.9 (5.5)	5.0 (4.9)	0.3 (0.4)
Val 99	5.2	5.9 (6.1)	5.4 (5.2)	5.0 (5.8)	6.6 (6.1)	5.7 (5.8)	0.6 (0.3)
Asp 101	7.0	6.1 (7.9)	7.3 (7.3)	6.4 (6.3)	6.6 (5.8)	6.6 (6.8)	0.4 (0.8)
Asn 103	8.2	*5.4* (6.6)	*5.6* (8.9)	6.4 (9.4)	6.8 (9.2)	*6.1* (8.5)	0.6 (1.1)
Met 105	7.4	7.0 (3.8)	*4.6* (5.4)	6.5 (6.2)	6.4 (7.4)	6.1 (5.7)	0.9 (1.3)
Ala 107	4.2	5.2 (4.2)	5.8 (4.2)	5.6 (3.6)	6.0 (4.5)	5.7 (4.1)	0.3 (0.3)
Trp 108	9.6	*5.9* (8.7)	*6.2* (8.9)	*6.0* (8.1)	*5.7* (9.1)	*6.0* (8.7)	0.2 (0.4)
Val 109	4.0	4.7 (3.8)	4.4 (4.1)	5.1 (5.3)	4.6 (4.3)	4.7 (4.4)	0.3 (0.6)
Trp 111	7.1	5.4 (*4.8*)	5.4 (*4.6*)	*5.0* (*4.8*)	*5.0* (*4.6*)	5.2 (*4.7*)	0.2 (0.1)
Arg 112	4.5	4.1 (4.3)	4.5 (4.5)	6.4 (4.2)	4.4 (3.3)	4.9 (4.1)	0.9 (0.5)
Asn 113	5.8	7.3 (7.2)	*8.2* (*8.1*)	6.1 (6.7)	6.3 (5.6)	7.0 (6.9)	0.8 (0.9)
Arg 114	9.6	*7.3* (9.6)	*5.0* (9.6)	8.0 (9.6)	*7.3* (9.7)	*6.9* (9.6)	1.1 (0.1)
Cys 115	9.8 (9.7)	*6.2* (9.6)	7.7 (9.7)	8.4 (9.6)	*6.4* (9.3)	*7.2* (9.5)	0.9 (0.2)
Thr 118	9.8 (9.7)	*6.8* (9.5)	*7.1* (8.8)	*6.7* (7.9)	*6.9* (8.9)	*6.9* (8.8)	0.1 (0.6)
Asp 119	6.7	5.9 (6.5)	5.5 (6.9)	6.1 (6.6)	6.3 (6.3)	6.0 (6.6)	0.3 (0.2)
Val 120	4.6	6.4 (5.0)	4.9 (6.1)	*6.7* (5.3)	5.8 (5.0)	6.0 (5.4)	0.7 (0.5)
Gln 121	5.0	4.2 (4.0)	5.4 (5.0)	4.5 (4.4)	4.9 (4.1)	4.8 (4.4)	0.5 (0.4)
Ala 122	3.7	4.9 (3.5)	4.3 (3.5)	4.6 (3.6)	4.6 (2.8)	4.6 (3.4)	0.2 (0.3)
Trp 123	5.4	6.0 (5.8)	5.5 (6.2)	6.3 (5.4)	6.4 (4.9)	6.1 (5.6)	0.4 (0.5)
Ile 124	10.6 (9.7)	*6.7* (9.6)	*6.7* (9.7)	*7.0* (9.4)	*7.1* (9.5)	*6.9* (9.6)	0.2 (0.1)
Arg 125	4.4	5.9 (4.7)	6.2 (4.7)	5.5 (4.0)	6.4 (5.2)	6.0 (4.7)	0.3 (0.4)
Cys 127	7.7	*5.6* (7.6)	6.0 (7.5)	6.4 (6.4)	7.1 (8.9)	6.3 (7.6)	0.6 (0.9)
Arg 128	8.0	6.8 (6.0)	6.8 (6.7)	7.4 (8.5)	7.4 (7.8)	7.1 (7.3)	0.3 (1.0)
Leu 129	9.0	7.5 (9.6)	7.5 (9.2)	7.5 (9.4)	7.3 (9.3)	7.5 (9.4)	0.1 (0.1)

Experimental values from Table II of Smith et al. (1991). The value within brackets in the column “Experimental value” represents the maximum in the Karplus relation (Pardi et al. 1984) used for the calculation of the ^3^*J*_*HNHα*_-couplings. The values within brackets in the six “MD simulation (X-ray crystal)” columns represent the ^3^*J*_*HNHα*_-couplings calculated from the four X-ray crystal structures, their mean and RMSD from it. MD or X-ray values differing more than 2 Hz from the experimental value and the maximum of the Karplus curve are denoted using italics

The distribution of these ^3^*J*_*HN-Hα*_-coupling and ^3^*J*_*Hα-Hβ*_-coupling values over the protein is shown in Fig. 1 (backbone experimentally assigned (*bb1*): blue; backbone computationally stereo-specifically assigned (*bb2*): magenta; side chain experimentally stereo-specifically assigned (*sc1*): green; side chain computationally stereo-specifically assigned (*sc2*): red).

**Fig. 2:**
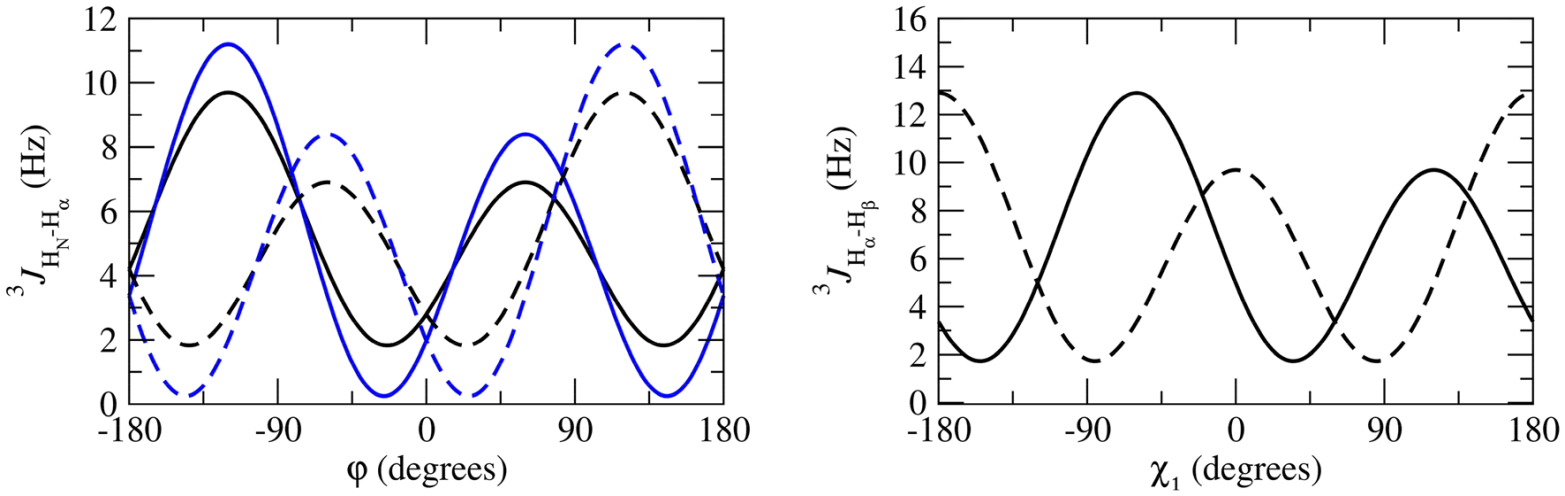
Left panel: ^3^*J*_*HNHα*_-coupling Karplus curves as function of the *φ*-angle (black lines: from Pardi et al. 1984; blue lines: from Brüschweiler and Case 1994; solid lines: *α* hydrogen and *α2 Re* hydrogen for Gly; dashed lines: *α3 Si* hydrogen for Gly). Right panel: ^3^*J*_*Hα-Hβ*_-coupling Karplus curves as function of the *χ*_1_-angle from deMarco et al. 1978 (solid line: β hydrogen for Ile and Thr and β_*2*_ hydrogen, dashed line: β hydrogen for Val and β_*3*_ hydrogen)

For the calculation of the backbone ^3^*J*_*HN-Hα*_-couplings, the Karplus relation Eq. (2) was used with the parameter values *a* = 6.4 Hz, *b* = − 1.4 Hz and *c* = 1.9 Hz (Pardi et al. 1984), see Fig. 2, left panel (black lines). The side-chain ^3^*J*_*Hα-Hβ*_-couplings were calculated using the parameter values *a* = 9.5 Hz, *b* = − 1.6 Hz and *c* = 1.8 Hz (deMarco et al. 1978), see Fig. 2, right panel (black lines).

**Fig. 3:**
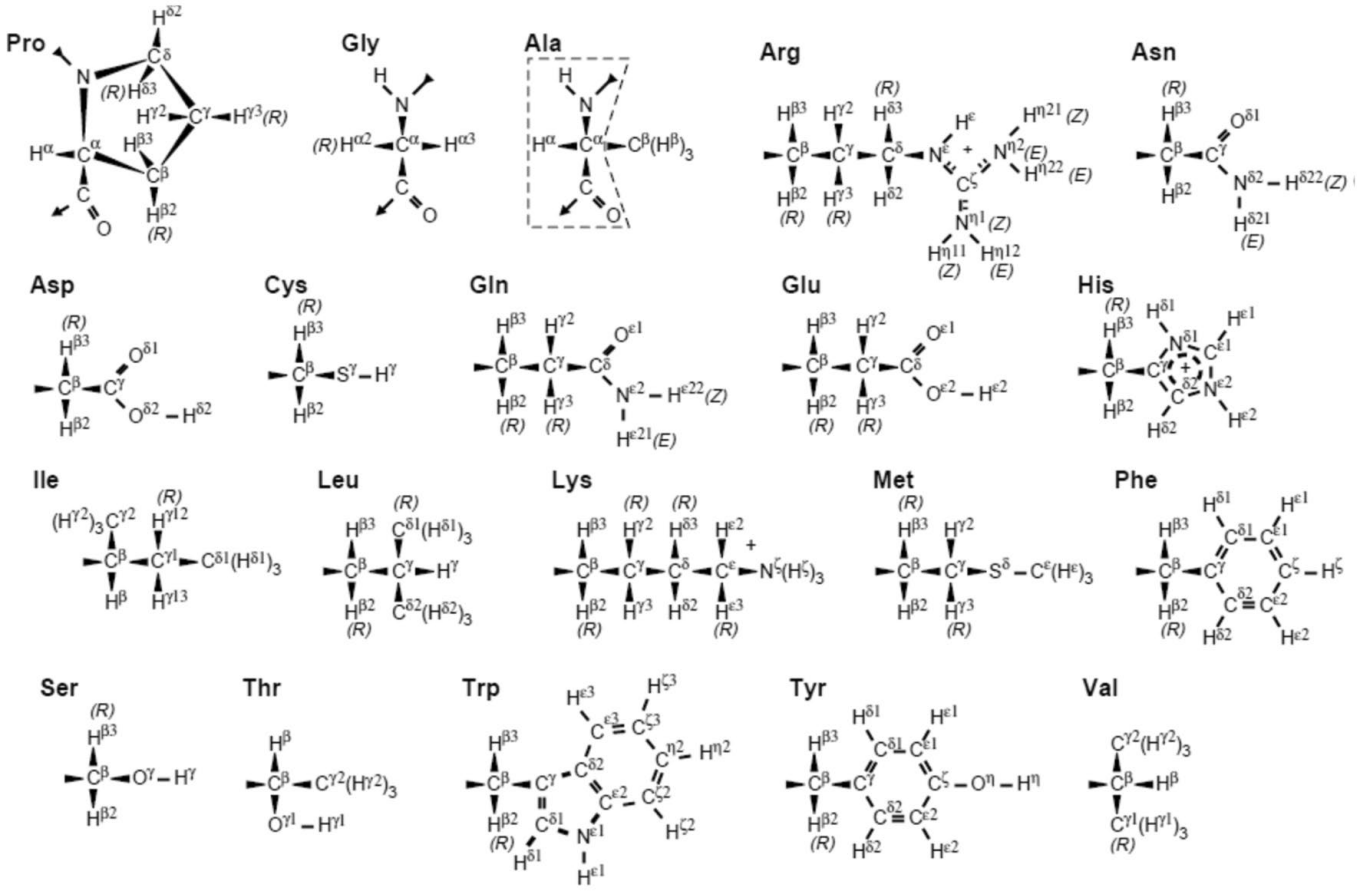
Recommended atom identifiers for the 20 common amino acids follow the 1969 IUPAC-IUB guidelines (IUPAC-IUB Commission on Biochemical Nomenclature 1970). Backbone atoms are shown for Pro, Gly, and Ala but not for the other L-amino acids (where they correspond to those bounded by the dashed line in the Ala structure). Greek letters are used as atom identifiers. Figure taken from Markley et al. (1998, Fig. 1)

The experimentally derived ^3^*J*_*HN-Hα*_-coupling values for Val 2, Thr 51, Asp 66, Cys 115, Thr 118 and Ile 124 lie outside the Karplus curve, so were set to 9.7 Hz, which is the maximum of the Karplus curve used (Pardi et al. 1984). None of the experimentally derived ^3^*J*_*Hα-Hβ*_-coupling values lie outside the Karplus curve used (deMarco et al. 1978). The nomenclature for the H_α2_ and H_α3_ atoms in Gly residues and the H_β_, H_β2_ and H_β3_ atoms in the side chains was defined as in Markley et al. (1998), see Fig. 3.

The initial implementation of the algorithm proposed in Smith et al. (2016) in the GROMOS MD program was incorrect. The factors *t*^−1^ and d*t* in the expressions of Eqs. (6) and (7) (Eqs. (21) and (22) in Smith et al. 2016) were not implemented, which meant that the local-elevation weight factors *ω*_*φ*_ or *ω*_*θ*_ were not time-averaged and of different magnitude than intended. They would thus only stay constant instead of decrease when the restraints were satisfied. This implementation error has been corrected. As a consequence, the values of the restraining force constants *K*^*Jr*^ used in the previous work (Smith et al. 2016) are much smaller than the value of the force constant *K*^*Jr*^ used in the present work. In Smith et al. (2016), *K*^*Jr*^ was varied between 5 and 50⋅10^–4^ kJ mol^−1^ Hz^−4^. Here, *K*^*Jr*^ is set to 50 kJ mol^−1^ Hz^−4^, because the weights *ω*_*θ*_ are much smaller. In Smith et al. (2016), the memory relaxation time was varied between 5 and 50 ps, because of the length of 2 ns of the many test simulations. Here it could be extended to *τ*_*Q*_ ≡ *τ*_*J*_ = 500 ps, in view of the 20 ns length of the MD simulations. In Smith et al. (2016), the flat-bottom parameter of the restraining potential energy term was varied between 0.5 and 1 Hz. Here the value Δ^3^*J*^*fb*^ = 1.0 Hz is used, which means a flat bottom of 2 Hz width (Smith et al. 2016), representing the uncertainty of the Karplus relation used.

### MD simulations performed

Four unrestrained MD simulations, starting from the four mentioned X-ray crystal structures, were performed:

1. *MD_2VB1*,
2. *MD_4LZT*,
3. *MD_1IEE*,
4. *MD_1AKI*,

each 20 ns long. The average solute temperatures were 311 K and the solvent temperatures 312 K.

Starting from the *2VB1* X-ray crystal structure, four ^3^*J*-restraining MD simulations were performed:

5. *MD_2VB1_bb1* + *bb2*, applying ^3^*J*_*HN-Hα*_-coupling restraining to sets *bb1* and *bb2*, 117 restraints,
6. *MD_2VB1_sc1*, applying ^3^*J*_*Hα-Hβ*_-coupling restraining to set *sc1*, 58 restraints,
7. *MD_2VB1_sc1* + *sc2*, applying ^3^*J*_*Hα-Hβ*_-coupling restraining to sets *sc1* and *sc2* (without Glu 7), 96 restraints,
8. *MD_2VB1_bb1* + *bb2* + *sc1* + *sc2*, applying ^3^*J*_*HN-Hα*_-coupling and ^3^*J*_*Hα-Hβ*_-coupling restraining to sets *bb1*, *bb2*, *sc1* and *sc2* (without Glu 7), 213 restraints, again each 20 ns long. The average solute and solvent temperatures were as mentioned above.

### Analysis of atomic trajectories and X-ray structures

Trajectory energies and atomic coordinates were stored at 5 ps intervals and used for analysis (Eichenberger et al. 2011).

In view of the various factors contributing to an uncertainty of about 2 Hz inherent to the Karplus relation linking structure and ^3^*J*-couplings, as discussed in the Introduction, a deviation of less than 2 Hz between ^3^*J*-coupling values calculated from X-ray or MD trajectory structures and ^3^*J*-coupling values derived from experiment is considered insignificant.

The GROMOS force fields treat aliphatic carbons as united CH, CH_2_ and CH_3_ atoms. So inter-hydrogen distances involving the aliphatic hydrogen atoms were calculated using virtual atomic positions for CH and pro-chiral CH_2_ (van Gunsteren et al. 1985) and pseudo-atomic positions for CH_3_ (Wüthrich et al. 1983) for those hydrogen atoms (van Gunsteren et al. 2019). The pseudo-atom NOE distance bound corrections of Wüthrich et al. (1983) were used (van Gunsteren et al. 2016). The set of NOE distance bounds (Smith et al. 1993; Schwalbe et al. 2001) can be found in Table S1 of Supporting Information, together with values of the five simulations starting from the *2VB1* X-ray crystal structure. The NOE between Trp 28 HZ3 and Leu 56 HG was reassigned as between Trp 28 HZ3 and Leu 56 HD* following reassessment of the experimental spectra. Inter-hydrogen distances were calculated as  〈*r*^−3^〉 ^−1/3^, i.e. using *r*^−3^ averaging over the trajectory structures, where *r* indicates the actual hydrogen–hydrogen distance. In view of the uncertainty inherent to the calculation of NOE bounds and *r*^−3^ averaged distances, deviations from experiment of less than 0.1 nm are considered insignificant.

*S*^2^ order parameters were calculated using the ensemble averaging expression (Henry and Szabo 1985)

11 $$S_{XY}^{2} = \frac{1}{2}\left\{ {3\sum\limits_{\alpha = 1}^{3} {\sum\limits_{\beta = 1}^{3} {\left\langle {\frac{{\mu_{XY\alpha } (t)\mu_{XY\beta } (t)}}{{r_{XY}^{3} (t)}}} \right\rangle_{\tau}^{2} } } - \left\langle {\frac{1}{{r_{XY}^{3} (t)}}} \right\rangle_{\tau}^{2} } \right\}(r_{XY}^{eff} )^{6} ,$$

where τ indicates the time-averaging window, here 1 ns, shorter than the rotational correlation time of 5.7 ns of HEWL in solution (Smith et al. 1993),

12 $$\mu_{XY1} \equiv \, \left( {x_{X} {-}x_{Y} } \right)/r_{XY} ,\mu_{XY2} \equiv \, \left( {y_{X} {-}y_{Y} } \right)/r_{XY} ,\mu_{XY3} \equiv \, \left( {z_{X} {-}z_{Y} } \right)/r_{XY} ,$$

are the normalised *x*-, *y*-, and *z*-components of the vector ***r***_*XY*_ ≡ ***r***_*X*_ − ***r***_*Y*_ connecting atoms X and Y, and *r*_*XY*_ ≡ |***r***_*XY*_| its length (Hansen et al. 2014). To obtain a dimensionless quantity the term in curly brackets is multiplied with the 6th power of the effective length (*r*^*eff*^_*XY*_) of the vector ***r***_XY_. Because in the present work bond length constraints are used, the length of ***r***_XY_ is essentially constant over time and thus equal to its effective value *r*^*eff*^_*XY*_. The set of 79 experimentally derived *S*^2^ values (Buck et al. 1995; Moorman et al. 2012) together with the *S*^2^ values calculated from the five MD simulations started from the *2VB1* X-ray crystal structure (Smith et al. 2020) can be found in Supporting Information, Table S2.

Before calculating *S*^2^_*XY*_, the protein trajectory structures are superimposed using the backbone atoms (N, C_α_, C) of residues 3–126 in the fit in order to eliminate the effect of overall rotation of the protein upon the *S*^2^_*XY*_-values. Use of only the backbone atoms of four of the five α-helices and two β-strands in HEWL (residues 4–15, 24–36, 41–45, 50–53, 89–99, and 108–115) did not lead to significantly different *S*^2^_*XY*_-values.

For the Asn and Gln residues, one *S*^2^_*NH*_*(exp)* value per NH_2_ group was available. This required the assignment to one of the two NH1 and NH2 bond vectors. This was done by calculating *S*^2^_*NH1*_*(MD)-* and *S*^2^_*NH2*_*(MD)*-values from the unrestrained simulation *MD_2VB1* starting from the *2VB1* X-ray structure and then selecting the N–H vector with its *S*^2^_*NH*_*(MD)* value closest to *S*^2^_*NH*_*(exp)* for restraining. A corresponding procedure was used to assign experimentally unassigned *S*^2^_*CG1*_- and *S*^2^_*CG2*_-values for Val and *S*^2^_*CD1*_- and *S*^2^_*CD2*_-values for Leu residues (Smith et al. 2020).

In view of the uncertainty inherent to the derivation of *S*^2^_*XY*_*(exp)*-values from relaxation experiments and inherent to the calculation of *S*^2^_*XY*_*(MD)*-values from MD simulation, a deviation of less than 0.2 between simulation and experiment is considered insignificant.

Atom-positional root-mean-square fluctuations, i.e. around their average positions, in the MD trajectories were calculated using the above-mentioned superposition too.

The secondary structure assignment was done with the program DSSP, based on the Kabsch–Sander rules (Kabsch and Sander 1983).

Hydrogen bonds were identified according to a geometric criterion: a hydrogen bond was assumed to exist if the hydrogen-acceptor distance was smaller than 0.25 nm and the donor-hydrogen-acceptor angle was larger than 135°.

## Results and discussion

### Comparison of ^3^*J*-coupling values calculated from X-ray structures or MD trajectories with NMR measured values

Table 1 lists 95 backbone ^3^*J*_*HNHα*_-coupling values derived and stereo-specifically assigned based on NMR measurements (set *bb1*), as calculated from the four unrestrained MD simulations starting from four X-ray crystal structures, and as calculated from the four X-ray structures. The mean of the four MD or X-ray values and the root-mean-square deviation (RMSD) from it are also presented. Deviations from the experimental values of more than 2 Hz are denoted in italics. The mean values of the MD simulations show 14 deviations larger than 2 Hz, while for the X-ray structures there is only one such case (Trp 111). The great majority of the large differences between MD simulation and experiment are found for residues (11), for which large ^3^*J*_*HNHα*_-coupling values (> 9 Hz) have been observed (Ser 36, Gln 41, Asn 65, Arg 68, Thr 69, Leu 84, Trp 108, Arg 114, Cys 115, Thr 118 and Ile 124). This can be explained by considering the Karplus curve in Fig. 2 (left panel). Its maximum of 9.7 Hz lies at *φ* ≈ 240° or − 120° and ^3^*J*_*HNHα*_-coupling values larger than 8 Hz are only found for *φ*-angle values between 210° and 270°. Any motion around *φ* ≈ 240° will lower the calculated ^3^*J*_*HNHα*_-coupling value. We note that other parametrisations of the Karplus curve show maxima of 10 Hz (Wang and Bax 1996) or even 11 Hz (Brüschweiler and Case 1994), see Fig. 1 in Dolenc et al. (2010). The remaining three residues with ^3^*J*_*HNHα*_-coupling value differences with experiment larger than 2 Hz are found for Ser 50, Ile 78 and Asn 103, whose experimental values are 7.8, 8.0 and 8.2 Hz, respectively. For the X-ray structures, the only ^3^*J*_*HNHα*_-coupling value differing more than 2 Hz from experiment (7.1 Hz) is for Trp 111, where all four values are smaller than 4.9 Hz. Use of the Karplus relation of Brüschweiler and Case (1994) (Fig. 2, left panel, blue lines) slightly lowers the ^3^*J*_*HNHα*_-coupling values smaller than 6.3 Hz and increases values larger than 6.3 Hz, the increase being about 1 Hz for ^3^*J*_*HNHα*_-coupling values larger than 8.5 Hz. It does not improve significantly the agreement of the ^3^*J*_*HNHα*_-coupling values obtained from the MD simulations and the X-ray structures with the experimental ones.

Table 2 lists 22 experimentally stereo-specifically unassigned backbone ^3^*J*_*HNHα*_-coupling values derived from NMR measurements (set *bb2*), as calculated from the four unrestrained MD simulations starting from four X-ray crystal structures, and as calculated from the four X-ray structures. The mean of the four MD or X-ray values and the root-mean-square deviation (RMSD) from it are also presented. The stereo-specific assignment of the experimental values for the α2 *Re* and the α3 *Si* hydrogens in glycine residues is based on the criterion that the ^3^*J*_*HN-Hα*_-coupling values calculated from the four unrestrained MD simulations starting from the four X-ray structures do suggest in 4 or 3 of the unrestrained MD simulations the same assignment. Only Gly 104 could not be stereo-specifically assigned using this criterion. For this residue the stereo-specific assignment was based on the ^3^*J*_*HN-Hα*_-coupling values calculated from the four X-ray structures using a corresponding criterion. For only two residues the X-ray structures would suggest stereo-specific assignments different from the ones based on the MD trajectories, for Gly 67, which shows a rather small difference of 0.7 Hz between the two experimental ^3^*J*_*HN-Hα*_-coupling values, and for Gly 126, with a somewhat larger difference of 1.2 Hz between the two experimental ^3^*J*_*HN-Hα*_-coupling values. Deviations of the MD or X-ray calculated ^3^*J*_*HN-Hα*_-coupling values from the experimental ones of more than 2 Hz are denoted in italics. The MD trajectories show four of such deviations, for Gly 49 α2 *Re* in the *MD_2VB1* simulation, for Gly 104 α2 *Re* and α3 *Si* in the *MD_4LZT* simulation and α3 *Si* in the *MD_1AKI* simulation. The X-ray structures show seven of such deviations, for Gly 102 α2 *Re* and α3 *Si* in the *1IEE* and *1AKI* structures, for Gly 104 α3 *Si* in the *2VB1* structure and for Gly 126 α3 *Si* in the *4LZT* and *1AKI* structures.

**Table 2:** Experimentally stereo-specifically unassigned backbone ^3^*J*_*HNHα*_-coupling values (22) in Hz derived from NMR measurements (set *bb2*) and values from four unrestrained MD simulations starting from four X-ray crystal structures, and the mean of the latter four values and the root-mean-square deviation (RMSD) from it

Residue	Experimental value	MD simulation (X-ray crystal)					
		*2VB1*	*4LZT*	*1IEE*	*1AKI*	Mean	RMSD
Gly 4							
α2 *Re*	8.0	7.5 (7.3)	7.8 (6.8)	7.2 (5.8)	6.8 (7.2)	7.3 (6.8)	0.4 (0.6)
α3 *Si*	6.1	4.9 (5.9)	5.0 (6.2)	5.4 (6.6)	5.7 (6.0)	5.2 (6.2)	0.3 (0.3)
Gly 16							
α2 *Re*	6.1	5.3 (5.9)	4.1 (5.8)	5.0 (6.1)	6.2 (6.0)	5.1 (5.9)	0.7 (0.1)
α3 *Si*	6.2	6.3 (7.4)	6.7 (7.5)	6.5 (7.0)	5.6 (7.2)	6.3 (7.3)	0.4 (0.2)
Gly 22							
α2 *Re*	6.0	6.1 (6.2)	5.4 (6.0)	6.1 (5.9)	5.8 (5.6)	5.9 (5.9)	0.3 (0.2)
α3 *Si*	6.8	6.4 (6.9)	5.4 (7.1)	6.3 (7.3)	6.9 (7.7)	6.3 (7.3)	0.5 (0.3)
Gly 26							
α2 *Re*	3.3	3.9 (4.4)	4.0 (4.3)	4.1 (4.4)	4.2 (3.3)	4.1 (4.1)	0.1 (0.4)
α3 *Si*	6.2	6.7 (6.9)	6.8 (6.9)	6.8 (6.9)	6.8 (6.8)	6.8 (6.9)	0.0 (0.0)
Gly 49							
α2 *Re*	5.3	*3.1* (5.6)	3.3 (5.1)	3.5 (5.2)	3.4 (5.0)	3.3 (5.2)	0.2 (0.2)
α3 *Si*	8.0	9.1 (7.8)	9.0 (8.3)	8.8 (8.3)	8.7 (8.4)	8.9 (8.2)	0.1 (0.2)
Gly 67							
α2 *Re*	5.5	6.0 (6.6)	6.1 (6.5)	6.3 (6.7)	5.8 (6.8)	6.0 (6.6)	0.2 (0.1)
α3 *Si*	6.2	6.4 (5.8)	6.2 (6.2)	6.0 (5.6)	6.4 (5.3)	6.2 (5.7)	0.2 (0.3)
Gly 71							
α2 *Re*	5.9	6.2 (6.7)	6.6 (6.5)	6.6 (5.6)	6.6 (6.5)	6.5 (6.3)	0.2 (0.4)
α3 *Si*	5.7	5.5 (5.4)	4.6 (6.1)	4.8 (7.8)	5.1 (6.1)	5.0 (6.4)	0.3 (0.9)
Gly 102							
α2 *Re*	6.2	4.2 (5.6)	4.5 (5.6)	4.6 (*1.9*)	6.3 (*1.9*)	4.9 (*3.8*)	0.8 (1.9)
α3 *Si*	6.4	6.7 (7.7)	6.6 (7.8)	6.8 (*9.0*)	5.3 (*9.1*)	6.4 (8.4)	0.6 (0.6)
Gly 104							
α2 *Re*	6.4	6.7 (6.5)	*4.2* (8.4)	6.4 (6.9)	4.8 (6.9)	5.5 (7.2)	1.1 (0.7)
α3 *Si*	3.9	4.5 (*6.3*)	*6.6* (5.1)	4.8 (4.1)	*6.3* (3.7)	5.5 (4.8)	0.9 (1.0)
Gly 117							
α2 *Re*	6.2	5.3 (6.2)	5.8 (6.2)	5.1 (5.8)	5.0 (5.9)	5.3 (6.0)	0.3 (0.2)
α3 *Si*	6.5	6.1 (6.9)	5.5 (6.8)	6.3 (7.5)	6.4 (7.3)	6.1 (7.1)	0.3 (0.3)
Gly 126							
α2 *Re*	6.8	6.5 (6.2)	6.3 (5.6)	6.3 (5.9)	6.1 (4.8)	6.3 (5.6)	0.1 (0.5)
α3 *Si*	5.6	5.3 (6.9)	5.9 (*7.7*)	5.5 (7.3)	5.3 (*8.7*)	5.5 (7.6)	0.3 (0.7)

Experimental values from Table II of Smith et al. (1991). The values within brackets in the six “MD simulation (X-ray crystal)” columns represent the ^3^*J*_*HNHα*_-couplings calculated from the four X-ray crystal structures, their mean and RMSD values. The stereo-specific assignment of the experimental values for the α2 *Re* and the α3 *Si* hydrogens in glycine residues is based on the criterion that the ^3^*J*_*HN-Hα*_-coupling values calculated from the four unrestrained MD simulations starting from the four X-ray structures do suggest in 4 or 3 of the unrestrained MD simulations the same stereo-specific assignment. Only Gly 104 could not be stereo-specifically assigned using this criterion. For this residue the stereo-specific assignment was based on the ^3^*J*_*HN-Hα*_-coupling values calculated from the four X-ray structures using a corresponding criterion. MD or X-ray values differing more than 2 Hz from the experimental value and the maximum of the Karplus curve are denoted using italics

Table 3 lists 58 side-chain ^3^*J*_*HαHβ*_-coupling values derived and stereo-specifically assigned based on NMR measurements (set *sc1*), as calculated from the four unrestrained MD simulations starting from four X-ray crystal structures, and as calculated from the four X-ray structures. The mean of the four MD or X-ray values and the root-mean-square deviation (RMSD) from it are also presented. Deviations from the experimental values larger than 2 Hz are denoted in italics. Out of 58*4 = 232 ^3^*J*_*HαHβ*_-coupling values, the MD trajectories yield 58 values 2 Hz larger than experiment, and the X-ray structures 55 values. The MD trajectories show a variation in ^3^*J*_*HαHβ*_-coupling values larger than 2 Hz for seven hydrogens in five residues, Tyr 23 β_2_, Asn 27 β_2_, Phe 34 β_2_ and β_3_, Asn 46 β_2_ and β_3_, and Cys 127 β_2_. The X-ray structures show two such cases, for Val 99 and Val 109. In all these cases the mean of the four MD or X-ray values lies within 2 Hz from experiment, but the four values contributing to the mean show a large variation of 2.2–3.7 Hz for the MD values and 3.4–4.6 for the X-ray values. Apparently, MD trajectories and different X-ray structures contain different side-chain *χ*_1_-angle conformers for these residues.

**Table 3:** Side-chain ^3^*J*_*HαHβ*_-coupling values (58) in Hz derived and stereo-specifically assigned based on NMR measurements (set *sc1*) and from four unrestrained MD simulations starting from four X-ray crystal structures, and the mean of the latter four values and the root-mean-square deviation (RMSD) from it

Residue	Expt value	MD simulation (X-ray crystal)					
		*2VB1*	*4LZT*	*1IEE*	*1AKI*	Mean	RMSD
Val 2	10.8	9.3 (12.9)	10.0 (12.9)	9.8 (12.9)	9.3 (12.9)	9.6 (12.9)	0.3 (0.0)
Cys 6							
β_2_	11.5	12.6 (12.7)	12.6 (12.8)	*8.9* (12.7)	12.3 (12.5)	11.6 (12.7)	1.6 (0.1)
β_3_	3.5	3.4 (2.5)	3.1 (2.7)	5.3 (2.5)	2.8 (2.3)	3.6 (2.5)	1.0 (0.1)
His 15							
β_2_	11.2	11.9 (12.9)	12.3 (12.5)	12.0 (12.6)	11.0 (11.4)	11.8 (12.4)	0.5 (0.6)
β_3_	2.6	2.4 (3.1)	2.6 (2.2)	2.5 (2.4)	3.1 (1.8)	2.6 (2.4)	0.3 (0.5)
Asp 18							
β_2_	4.2	5.1 (3.2)	2.9 (2.3)	3.2 (*1.9*)	3.4 (*2.1*)	3.7 (2.4)	0.9 (0.5)
β_3_	11.0	*7.5* (12.9)	11.1 (12.5)	10.0 (12.0)	*8.0* (12.3)	9.2 (12.4)	1.5 (0.3)
Tyr 20							
β_2_	2.3	*7.2* (3.0)	*5.7* (3.2)	*9.9* (2.4)	*8.7* (2.3)	*7.9* (2.7)	1.6 (0.4)
β_3_	11.7	*6.9* (12.9)	*9.6* (12.9)	*5.0* (12.6)	*5.4* (12.5)	*6.7* (12.7)	1.8 (0.2)
Tyr 23							
β_2_	10.9	12.6 (12.5)	*7.1*(12.7)	12.0 (12.5)	11.8(12.7)	10.9 (12.6)	2.2 (0.1)
β_3_	2.7	3.1 (2.2)	*5.8* (2.5)	3.5 (2.3)	3.3 (2.5)	3.9 (2.4)	1.1 (0.1)
Asn 27							
β_2_	10.3	*3.6* (11.6)	12.2 (12.2)	12.1 (11.6)	12.3 (12.2)	10.0 (11.9)	3.7 (0.3)
β_3_	2.4	4.3 (1.8)	3.3 (2.0)	3.4 (1.8)	4.3 (2.0)	3.8 (1.9)	0.5 (0.1)
Val 29	11.1	10.1 (12.8)	10.5 (12.9)	9.5 (12.8)	*7.9* (12.9)	9.5 (12.8)	1.0 (0.0)
Cys 30							
β_2_	5.3	*3.2* (*3.0*)	3.5 (*2.9*)	*3.1* (*2.7*)	3.5 (*2.4*)	3.3 (*2.7*)	0.2 (0.2)
β_3_	10.8	12.6 (*12.9*)	12.6 (12.8)	12.6 (12.8)	12.7 (12.6)	12.6 (12.8)	0.0 (0.1)
Phe 34							
β_2_	10.7	10.7 (*12.8*)	11.3 (12.7)	11.8 (*12.9*)	*4.2* (12.5)	9.5 (12.7)	3.1 (0.1)
β_3_	5.0	3.5 (*2.6*)	*2.2* (*2.6*)	*2.2* (*2.9*)	*10.1* (*2.3*)	4.5 (*2.6*)	3.3 (0.2)
Asn 39							
β_2_	4.5	2.5 (2.6)	2.6 (2.8)	2.8 (2.6)	2.5 (*2.1*)	2.6 (2.5)	0.1 (0.3)
β_3_	10.8	12.0 (12.7)	12.1 (12.8)	11.9 (12.8)	11.9 (12.3)	12.0 (12.6)	0.1 (0.2)
Thr 40	4.5	2.7 (3.5)	2.9 (4.0)	2.9 (4.2)	2.8 (6.2)	2.8 (4.5)	0.1 (1.0)
Thr 43	3.7	3.4 (3.0)	3.0 (3.3)	2.9 (3.8)	3.9 (4.1)	3.3 (3.5)	0.4 (0.4)
Asn 46							
β_2_	11.2	*2.6* (12.9)	*8.4* (12.9)	11.8 (12.9)	*4.6* (12.9)	*6.8* (12.9)	3.5 (0.0)
β_3_	4.7	*9.2* (3.4)	6.0 (3.7)	3.9 (3.4)	*8.3* (3.6)	*6.8* (3.5)	2.1 (0.1)
Thr 47	2.6	3.0 (3.6)	2.9 (4.2)	3.0 (3.9)	2.9 (*5.1*)	2.9 (4.2)	0.1 (0.5)
Asp 48							
β_2_	2.6	4.2 (*5.3*)	4.2 (*4.7*)	4.3 (4.3)	4.2 (*5.5*)	4.2 (*4.9*)	0.0 (0.5)
β_3_	3.7	2.9 (2.1)	2.9 (2.4)	2.9 (2.6)	3.5 (2.0)	3.1 (2.3)	0.3 (0.2)
Thr 51	9.3	*5.6* (*12.9*)	9.6 (*12.9*)	10.7 (*12.9*)	8.7 (*12.8*)	8.7 (*12.9*)	1.9 (0.1)
Asp 52							
β_2_	11.6	12.6 (12.6)	12.5 (12.8)	12.4 (12.4)	12.5 (12.9)	12.5 (12.7)	0.1 (0.2)
β_3_	3.6	3.8 (4.7)	4.0 (4.0)	3.6 (2.2)	3.8 (3.9)	3.8 (3.7)	0.1 (0.9)
Tyr 53							
β_2_	10.4	12.1 (*12.7*)	12.2 (*12.7*)	12.0 (*12.8*)	12.2 (*12.7*)	12.1 (*12.7*)	0.1 (0.1)
β_3_	3.0	2.4 (2.5)	2.4 (2.6)	2.3 (2.9)	2.4 (2.5)	2.4 (2.6)	0.1 (0.2)
Asn 59							
β_2_	5.4	*2.3* (*2.8*)	*2.3* (*2.9*)	*2.2* (*1.9*)	*2.2* (*2.6*)	*2.3* (*2.5*)	0.1 (0.4)
β_3_	11.3	12.3 (12.8)	12.2 (12.8)	12.2 (11.9)	12.2 (12.8)	12.2(12.6)	0.1 (0.4)
Arg 61							
β_2_	5.7	5.5 (*2.6*)	5.6 (*2.4*)	5.3 (*2.9*)	*7.9* (*1.8*)	6.1 (*2.4*)	1.1 (0.4)
β_3_	10.8	*8.4* (12.7)	*7.7* (12.6)	*7.9* (12.8)	*5.6* (11.8)	*7.4* (12.5)	1.1 (0.4)
Asp 66							
β_2_	5.1	3.2 (4.5)	3.2 (4.6)	*3.0* (4.1)	*3.0* (4.6)	3.1 (4.4)	0.1 (0.2)
β_3_	4.5	*11.5* (2.5)	*9.7* (*2.4*)	*10.8* (2.7)	*12.1* (*2.4*)	*11.0* (2.5)	0.9 (0.1)
Thr 69	9.3	*6.1* (*12.9*)	9.1 (*12.9*)	9.4 (*12.7*)	*6.3* (*12.8*)	7.7 (*12.8*)	1.5 (0.1)
Leu 75							
β_2_	12.4	11.5 (12.9)	12.0 (12.9)	11.5 (12.9)	12.1 (12.5)	11.8 (12.8)	0.3 (0.2)
β_3_	2.1	3.0 (3.1)	2.8 (3.3)	2.9 (3.0)	*4.8* (2.3)	3.4 (2.9)	0.8 (0.4)
Asp 87							
β_2_	5.1	3.3 (4.1)	*2.7* (5.2)	3.3 (2.9)	3.2 (3.8)	3.1 (4.0)	0.3 (0.8)
β_3_	11.5	12.2 (12.8)	12.1 (12.4)	11.8 (12.9)	12.0 (12.9)	12.0(12.7)	0.1 (0.2)
Ile 88	4.5	4.3 (4.5)	2.8 (4.4)	4.8 (4.9)	2.8 (4.8)	3.7 (4.6)	0.9 (0.2)
Thr 89	9.5	*4.8* (*12.8*)	*4.2* (*12.7*)	*5.2* (*12.9*)	*4.9* (*12.8*)	*4.8* (*12.8*)	0.4 (0.1)
Val 92	10.1	9.6 (*12.5*)	*12.2* (*12.4*)	*7.5* (*12.7*)	10.3 (*12.3*)	9.9 (*12.5*)	1.7 (0.1)
Cys 94							
β_2_	4.0	2.6 (2.9)	2.3 (2.9)	2.5 (2.7)	2.6 (2.7)	2.5 (2.8)	0.1 (0.1)
β_3_	12.2	12.4 (12.8)	12.0 (12.8)	12.3 (12.8)	12.4 (12.8)	12.3 (12.8)	0.2 (0.0)
Val 99	6.3	*3.0* (*12.8*)	*3.3* (*12.8*)	6.0 (*2.2*)	4.5 (*2.4*)	*4.2* (7.6)	1.2 (5.3)
Val 109	8.0	9.0 (*3.2*)	6.8 (*12.9*)	*5.8* (*12.4*)	8.2 (*3.7*)	7.4 (8.1)	1.2 (4.6)
Thr 118	4.2	2.9 (5.2)	3.1 (5.4)	3.0 (5.3)	3.1 (6.1)	3.0 (5.5)	0.1 (0.3)
Asp 119							
β_2_	4.9	4.5 (3.1)	*2.8* (4.7)	2.9 (3.8)	3.0 (3.2)	3.3 (3.7)	0.7 (0.7)
β_3_	11.7	10.1(12.9)	12.3 (12.6)	12.1 (12.9)	12.2 (12.9)	11.7 (12.8)	0.9 (0.1)
Trp 123							
β_2_	10.6	12.2(11.9)	11.6 (12.5)	10.3 (12.6)	11.2 (*12.8*)	11.3 (12.4)	0.7 (0.3)
β_3_	2.9	3.7 (1.9)	2.9 (2.3)	*5.8* (2.4)	4.4 (2.8)	4.2 (2.3)	1.1 (0.3)
Ile 124	4.6	4.1 (4.4)	5.7 (4.3)	4.2 (3.7)	3.6 (4.6)	4.4 (4.2)	0.8 (0.4)
Cys 127							
β_2_	11.6	12.6 (12.9)	11.6 (12.9)	*4.4* (12.9)	12.4 (12.9)	10.3 (12.9)	3.4 (0.0)
β_3_	4.8	3.2 (3.3)	3.8 (3.4)	5.8 (3.8)	3.2 (3.7)	4.0 (3.5)	1.1 (0.2)

Experimental values from Tables III and IV of Smith et al. (1991). The values within brackets in the six “MD simulation (X-ray crystal)” columns represent the ^3^*J*_*HαHβ*_-couplings calculated from the four X-ray crystal structures, their mean and RMSD from it. MD or X-ray values differing more than 2 Hz from the experimental value are denoted using italics

Table 4 lists 40 experimentally stereo-specifically unassigned side-chain ^3^*J*_*HαHβ*_-coupling values derived from NMR measurements (set *sc2*), as calculated from the four unrestrained MD simulations starting from four X-ray crystal structures, and as calculated from the four X-ray structures. The mean of the four MD or X-ray values and the root-mean-square deviation (RMSD) from it are also presented. The stereo-specific assignment of the experimental values for the β_2_ and β_3_ hydrogens is based on the criterion that the ^3^*J*_*HαHβ*_-coupling values calculated from the four unrestrained MD simulations starting from the four X-ray structures do suggest in 4 or 3 of the unrestrained MD simulations the same stereo-specific assignment. Only Glu 7 could not be stereo-specifically assigned using this criterion. For four residues the X-ray structures would suggest stereo-specific assignments different from the ones based on the MD trajectories, for Asn 19, which shows a rather small difference of 0.9 Hz between the two experimental ^3^*J*_*HαHβ*_-coupling values, for Asn 37, with a larger difference of 3.9 Hz between the two experimental ^3^*J*_*HαHβ*_-coupling values, for Asn 74, with a difference of 6.6 Hz between the two ^3^*J*_*HαHβ*_-coupling values, and for Asn 77, with a difference of 3.4. Hz between the two ^3^*J*_*HαHβ*_-coupling values. Deviations of the MD or X-ray calculated ^3^*J*_*HαHβ*_-coupling values from the experimental ones of more than 2 Hz are denoted in italics. The MD trajectories show 45 of such deviations, for 13 residues, Glu 7, Lys 13, Arg 68, Ser 72, Asn 74, Asn 77, Ser 85, Ser 86, Ser 100, Asp 101, Asn 106, Arg 125 and Arg 128. The X-ray structures show 104 of such deviations, for 17 residues, Glu 7, Lys 13, Asn 19, Trp 28, Asn 37, Arg 45, Arg 68, Ser 72, Asn 74, Asn 77, Ser 85, Ser 86, Ser 100, Asp 101, Asn 106, Arg 125 and Arg 128. The MD trajectories show a variation in ^3^*J*_*HαHβ*_-coupling values larger than 2 Hz for four hydrogens in three residues, Ser 72 β_3_ (2.6 Hz), Asn 74 β_2_ (2.7 Hz), and Asp 101 β_2_ and β_3_ (both 3.2 Hz). The X-ray structures show 15 of such cases in nine residues, Glu 7 β_2_ (4.8 Hz) and β_3_ (5.2 Hz), Arg 45 β_2_ (4.1 Hz) and β_3_ (4.7 Hz), Arg 68 β_2_ (4.4 Hz), Ser 85 β_2_ (4.7 Hz) and β_3_ (3.9 Hz), Ser 86 β_2_ (3.5 Hz), Ser 100 β_2_ (2.9 Hz), Asp 101 β_2_ (4.1 Hz) and β_3_ (4.5 Hz), Arg 125 β_2_ (3.7 Hz) and β_3_ (5.4 Hz), and Arg 128 β_2_ (3.9 Hz) and β_3_ (4.3 Hz). In 6 of these 19 cases the mean of the four MD or X-ray values lies within 2 Hz from experiment, while the four values contributing to the mean show a large variation of 2.6–3.2 Hz for the MD values and 2.9–5.4 for the X-ray values. Apparently, MD trajectories and different X-ray structures contain different side-chain *χ*_1_-angle conformers for these residues.

**Table 4:** Experimentally stereo-specifically unassigned side-chain ^3^*J*_*HαHβ*_-coupling values (40) in Hz derived from NMR measurements (set *sc2* plus Glu 7) and values from four unrestrained MD simulations starting from four X-ray crystal structures, and the mean of the latter four values and the root-mean-square deviation (RMSD) from it

Residue	Expt value	MD simulation (X-ray crystal)					
		*2VB1*	*4LZT*	*1IEE*	*1AKI*	Mean	RMSD
Glu 7							
β_2_	6.7*	7.8 (*12.7*)	5.7 (*12.6*)	4.9 (*2.6*)	*9.4* (*3.7*)	7.0 (7.9)	1.7 (4.8)
β_3_	6.4*	7.0 (*2.6*)	*9.2* (*2.4*)	*10.0* (*12.7*)	6.0 (*12.9*)	8.1 (7.6)	1.6 (5.2)
Lys 13							
β_2_	5.1	5.7 (3.1)	6.3 (*3.0*)	*8.0* (3.6)	6.5 (3.7)	6.6 (3.3)	0.8 (0.3)
β_3_	9.2	8.6 (*12.9*)	8.1 (*12.9*)	*6.3* (*12.9*)	7.5 (*12.9*)	7.6 (*12.9*)	0.9 (0.0)
Asn 19							
β_2_	7.3	8.3 (*1.8*)	7.8 (*1.8*)	8.5 (*2.7*)	9.2 (*3.7*)	8.4 (*2.5*)	0.5 (0.8)
β_3_	6.4	5.9 (*11.4*)	5.7 (*11.5*)	5.5 (7.9)	4.5 (*12.9*)	5.4 (*10.9*)	0.6 (1.8)
Trp 28							
β_2_	10.7	12.6 (*12.8*)	12.4 (*12.9*)	12.4 (*12.8*)	12.4 (*12.9*)	12.4 (*12.8*)	0.1 (0.1)
β_3_	4.1	3.9 (2.8)	3.9 (3.3)	4.3 (2.6)	4.3 (3.3)	4.1 (3.0)	0.2 (0.3)
Asn 37							
β_2_	8.1	9.1 (*1.9*)	8.2 (*1.9*)	10.1 (*1.8*)	9.4 (*1.7*)	9.2 (*1.8*)	0.7 (0.1)
β_3_	4.2	5.1 (*10.0*)	4.9 (*9.8*)	4.4 (*10.4*)	4.9 (*11.0*)	4.9 (*10.3*)	0.3 (0.5)
Arg 45							
β_2_	6.9	8.0 (*12.8*)	8.5 (*12.9*)	8.6 (*3.8*)	8.5 (5.8)	8.4 (8.8)	0.3 (4.1)
β_3_	6.7	6.4 (*2.8*)	5.4 (*3.4*)	6.0 (*12.9*)	5.7 (*12.0*)	5.9 (7.8)	0.4 (4.7)
Cys 64							
β_2_	4.6	4.4 (3.6)	4.4 (3.6)	4.3 (3.7)	4.0 (3.5)	4.2 (3.6)	0.2 (0.1)
β_3_	2.7	2.7 (3.2)	2.7 (3.2)	2.8 (3.1)	3.0 (3.3)	2.8 (3.2)	0.1 (0.1)
Asn 65							
β_2_	4.5	4.2 (3.1)	3.2 (3.0)	5.6 (3.7)	3.7 (3.7)	4.2 (3.4)	0.9 (0.3)
β_3_	11.4	11.3 (12.9)	11.4 (12.9)	9.7 (12.9)	11.3 (12.9)	10.9 (12.9)	0.7 (0.0)
Arg 68							
β_2_	6.5	*10.1* (*12.8*)	*9.5* (*12.9*)	*10.1* (4.6)	*9.7* (*3.5*)	*9.9* (8.4)	0.2 (4.4)
β_3_	4.8	4.5 (*2.6*)	4.5 (3.5)	4.4 (*2.4*)	4.9 (3.2)	4.6 (2.9)	0.2 (0.4)
Ser 72							
β_2_	5.4	*7.5* (4.9)	3.5 (4.2)	3.6 (3.7)	3.7 (6.2)	4.5 (4.8)	1.7 (0.9)
β_3_	7.6	*5.2* (*12.5*)	*12.4* (*12.8*)	8.7 (*12.9*)	9.6 (*11.6*)	9.0 (*12.5*)	2.6 (0.5)
Asn 74							
β_2_	10.5	11.3 (*2.2*)	*5.2* (*2.1*)	9.9 (*2.2*)	*5.3* (*2.1*)	*7.9* (*2.1*)	2.7 (0.1)
β_3_	3.9	4.0 (*12.4*)	4.5 (*12.3*)	5.4 (*12.4*)	*8.8* (*12.3*)	5.7 (*12.4*)	1.9 (0.1)
Asn 77							
β_2_	8.3	*10.8* (*2.0*)	*10.6* (*1.9*)	*10.4* (*2.7*)	9.5 (*1.9*)	10.3 (*2.1*)	0.5 (0.3)
β_3_	5.9	*3.8* (*12.2*)	4.4 (*12.0*)	4.7 (*12.8*)	5.3 (*11.9*)	4.5 (*12.2*)	0.5 (0.3)
Ser 85							
β_2_	5.7	4.9 (*12.9*)	6.1 (*1.9*)	4.3 (*2.0*)	4.6 (*2.4*)	5.0 (4.8)	0.7 (4.7)
β_3_	7.4	*9.6* (*3.5*)	8.2 (*11.9*)	*10.6* (5.6)	9.2 (*12.6*)	9.4 (8.4)	0.9 (3.9)
Ser 86							
β_2_	6.4	*8.7* (*12.8*)	8.3 (*4.0*)	7.7 (*12.2*)	8.3 (*9.3*)	8.3 (*9.6*)	0.4 (3.5)
β_3_	4.1	3.9 (2.8)	3.9 (2.9)	4.0 (*2.0*)	3.7 (2.1)	3.9 (2.4)	0.1 (0.4)
Asn 93							
β_2_	10.8	10.7 (12.7)	11.1 (12.5)	9.5 (12.2)	11.6 (12.4)	10.7 (12.5)	0.7 (0.2)
β_3_	3.5	4.1 (2.5)	3.8 (2.3)	5.1 (2.0)	3.3 (2.2)	4.1 (2.2)	0.7 (0.2)
Ser 100							
β_2_	7.7	7.7 (6.1)	7.3 (*12.6*)	7.4 (*12.9*)	*4.2* (*12.8*)	6.7 (*11.1*)	1.4 (2.9)
β_3_	4.0	5.2 (*1.8*)	*6.4* (2.4)	5.1 (3.3)	5.4 (2.9)	5.5 (2.6)	0.5 (0.5)
Asp 101							
β_2_	5.6	*2.5* (*10.5*)	*3.3* (*10.5*)	*2.8* (*2.2*)	*10.2* (*2.4*)	4.7 (6.4)	3.2 (4.1)
β_3_	6.6	*12.2* (*1.8*)	*11.4* (*1.8*)	*11.4* (*12.4*)	*4.3* (8.4)	*9.8* (6.1)	3.2 (4.5)
Asn 106							
β_2_	10.5	*4.7* (9.3)	9.4 (*12.6*)	*5.5* (*12.9*)	*7.4* (*12.6*)	*6.8* (11.8)	1.8 (1.5)
β_3_	3.6	3.9 (2.1)	4.5 (2.3)	3.9 (3.0)	5.2 (2.4)	4.4 (2.4)	0.5 (0.3)
Arg 125							
β_2_	7.9	*10.4* (*2.6*)	*10.6* (*2.2*)	9.0 (*5.5*)	*11.0* (*11.4*)	*10.3* (*5.4*)	0.8 (3.7)
β_3_	6.1	*4.0* (*12.7*)	*3.8* (*12.5*)	4.5 (*2.0*)	*3.8* (*1.8*)	*4.0* (7.2)	0.3 (5.4)
Arg 128							
β_2_	7.9	9.3 (*12.9*)	8.7 (7.9)	9.8 (*2.3*)	9.3 (*10.5*)	9.2 (8.4)	0.4 (3.9)
β_3_	7.2	*4.8* (*3.2*)	*4.6* (9.0)	*4.7* (*12.5*)	*4.5* (*1.8*)	*4.6* (6.6)	0.1 (4.3)

Experimental values from Table III of Smith et al. (1991). The values within brackets in the six “MD simulation (X-ray crystal)” columns represent the ^3^*J*_*HαHβ*_-couplings calculated from the four X-ray crystal structures, their mean and RMSD values. The stereo-specific assignment of the experimental values for the β_2_ and β_3_ hydrogens is based on the criterion that the ^3^*J*_*HαHβ*_-coupling values calculated from the four unrestrained MD simulations starting from the four X-ray structures do suggest in 4 or 3 of the unrestrained MD simulations the same stereo-specific assignment. *Only Glu 7 could not be stereo-specifically assigned using this criterion. MD or X-ray values differing more than 2 Hz from the experimental value are denoted using italics

This analysis shows that neither the X-ray crystal structures nor the MD trajectories of HEWL in aqueous solution are wholly compatible with the 117 backbone ^3^*J*_*HNHα*_-coupling values and the 96 side-chain ^3^*J*_*HαHβ*_-coupling values as obtained from NMR experiments of this protein in aqueous solution. Application of biquadratic time-averaging local-elevation (BQ-TA-LER) ^3^*J*-coupling restraining in MD simulation should be able to produce atomic trajectories compatible with the experimental ^3^*J*-coupling values.

### Comparison of ^3^*J*-coupling values calculated from ^3^*J*-coupling time-averaging local-elevation restraining MD trajectories with NMR measured values

Table 5 lists 95 backbone ^3^*J*_*HNHα*_-coupling values derived and stereo-specifically assigned based on NMR measurements (set *bb1*) and calculated from the unrestrained and ^3^*J*-coupling time-averaging local-elevation restrained MD simulations starting from the *2VB1* X-ray crystal structure using different sets of backbone and side-chain restraints. The unrestrained MD simulation shows 17 ^3^*J*_*HNHα*_-coupling values (in italics) that deviate more than 2 Hz from the experimental values (residues 41, 46, 50, 56, 65, 68, 69, 78, 84, 87, 103, 108, 114, 115, 118, 124, 127). ^3^*J*_*HNHα*_-coupling time-averaging local-elevation restraining towards the sets *bb1* and *bb2* of 95 and 22 target backbone ^3^*J*_*HNHα*_-coupling values leads, as expected, to good agreement between simulation and experiment for the 95 backbone ^3^*J*_*HNHα*_-couplings. No deviations larger than 2 Hz are observed. Restraining towards the sets *sc1* or *sc1* and *sc2* of 58 and 38 side-chain ^3^*J*_*HαHβ*_-coupling values yields 18 or 20 deviations larger than 2 Hz, respectively. Side-chain ^3^*J*_*HαHβ*_-coupling restraining does not improve the agreement between simulation and experiment for the backbone ^3^*J*_*HNHα*_-couplings.

**Table 5:** Backbone ^3^*J*_*HNHα*_-coupling values (95) in Hz derived and assigned based on NMR measurements (set *bb1*) and from the unrestrained and ^3^*J*-coupling time-averaging local-elevation restrained MD simulations starting from the *2VB1* X-ray crystal structure and using different sets of backbone and side-chain restraints

Residue	Experimental value	Unrestrained MD	^3^*J*-coupling local-elevation restrained MD			
		*2VB1*	*2VB1*_*bb1* + *bb2*	*2VB1*_*sc1*	*2VB1*_*sc1* + *sc2*	*2VB1*_*bb1* + *bb2* + *sc1* + *sc2*
Val 2	10.0 (9.7)	7.9 (1.6)	9.0 (0.7)	*6.7* (1.6)	*7.5* (1.7)	9.0 (0.7)
Phe 3	7.4	7.5 (1.7)	8.0 (1.7)	8.9 (1.2)	6.0 (1.9)	7.8 (1.5)
Cys 6	5.8	5.5 (1.1)	5.6 (1.2)	5.4 (1.1)	4.9 (1.0)	5.4 (1.2)
Glu 7	4.5	4.3 (1.0)	4.5 (1.0)	4.5 (1.0)	4.4 (1.0)	4.4 (1.0)
Leu 8	5.5	4.7 (1.0)	4.5 (0.9)	4.5 (0.9)	4.7 (1.0)	4.9 (0.9)
Ala 9	3.7	4.2 (0.9)	4.2 (0.9)	4.2 (0.9)	4.2 (0.9)	4.1 (0.9)
Ala 10	3.9	4.5 (0.9)	4.5 (0.6)	4.5 (0.9)	4.5 (0.9)	4.5 (0.9)
Ala 11	4.8	4.5 (0.9)	4.7 (1.0)	4.7 (1.0)	4.5 (0.9)	4.6 (1.0)
Met 12	4.6	4.9 (1.0)	4.6 (0.9)	4.7 (1.0)	4.5 (0.9)	4.6 (0.9)
Lys 13	4.2	4.7 (1.0)	4.6 (1.0)	4.6 (1.0)	5.3 (1.2)	4.6 (1.0)
Arg 14	4.4	4.6 (1.0)	4.7 (1.1)	4.8 (1.2)	5.2 (1.5)	4.8 (1.1)
His 15	9.2	7.4 (1.6)	8.3 (1.2)	7.6 (1.6)	7.8 (1.5)	9.0 (0.9)
Leu 17	7.6	7.0 (1.7)	7.6 (1.6)	7.8 (1.6)	7.5 (1.8)	7.1 (1.2)
Asp 18	5.7	6.0 (1.9)	5.8 (1.6)	4.7 (1.1)	5.0 (1.4)	5.5 (1.9)
Asn 19	7.0	6.4 (1.4)	6.5 (0.5)	6.7 (0.3)	6.7 (0.3)	6.7 (0.3)
Tyr 20	5.5	7.0 (2.3)	5.6 (2.0)	4.6 (1.5)	5.1 (1.7)	5.0 (1.8)
Arg 21	6.8	6.0 (1.3)	6.4 (0.9)	6.7 (0.3)	6.7 (0.3)	6.7 (0.3)
Tyr 23	8.6	7.8 (1.9)	8.0 (1.7)	7.2 (2.1)	7.1 (2.0)	8.2 (1.5)
Asn 27	5.4	4.2 (1.0)	4.6 (1.1)	4.3 (1.0)	4.8 (1.0)	4.5 (1.0)
Trp 28	6.0	5.2 (1.0)	5.2 (1.0)	5.1 (1.0)	4.6 (1.0)	5.2 (1.1)
Val 29	5.9	4.8 (0.9)	4.8 (0.9)	4.6 (0.9)	4.7 (0.9)	4.8 (0.9)
Cys 30	3.8	4.6 (0.9)	4.4 (0.9)	4.5 (0.9)	4.6 (0.9)	4.7 (0.9)
Ala 31	3.8	4.4 (0.9)	4.2 (0.9)	4.2 (0.9)	4.4 (0.9)	4.3 (0.9)
Ala 32	4.8	4.5 (0.9)	4.3 (0.9)	4.3 (0.9)	4.4 (0.9)	4.4 (0.9)
Lys 33	3.6	4.6 (1.0)	4.6 (1.1)	4.8 (1.1)	4.6 (1.0)	4.5 (1.0)
Phe 34	7.6	5.6 (1.4)	7.2 (1.2)	6.4 (1.5)	*5.3* (1.4)	7.0 (1.3)
Glu 35	7.2	7.7 (1.5)	6.3 (1.4)	6.0 (1.5)	6.2 (1.4)	6.7 (1.6)
Ser 36	9.6	7.9 (1.7)	8.9 (1.1)	8.4 (1.4)	8.1 (1.6)	8.8 (1.2)
Phe 38	6.3	6.8 (0.2)	6.7 (0.5)	6.5 (0.9)	6.8 (0.2)	6.8 (0.2)
Asn 39	8.8	8.1 (1.5)	8.0 (1.4)	7.6 (1.6)	8.2 (1.6)	8.1 (1.5)
Thr 40	5.4	4.7 (1.1)	5.0 (1.1)	4.8 (1.2)	5.0 (1.3)	4.7 (1.0)
Gln 41	9.2	*6.5* (1.5)	8.4 (1.2)	*6.2* (1.4)	7.7 (1.5)	8.4 (1.1)
Ala 42	4.5	5.6 (1.6)	5.4 (1.6)	6.3 (1.9)	6.2 (1.9)	5.3 (1.5)
Thr 43	9.3	8.7 (1.3)	9.0 (1.0)	8.8 (1.1)	*6.2* (1.6)	8.8 (1.1)
Asn 44	9.4	7.7 (1.7)	8.5 (1.4)	7.4 (1.9)	*6.8* (2.2)	8.8 (1.2)
Arg 45	7.7	8.1 (1.4)	7.9 (1.6)	7.0 (1.7)	8.0 (1.7)	8.4 (1.4)
Asn 46	8.8	*6.1* (1.6)	8.5 (1.4)	6.8 (2.2)	6.9 (1.8)	8.6 (1.4)
Thr 47	4.4	4.2 (1.0)	4.4 (1.1)	5.3 (1.6)	4.1 (1.0)	4.4 (1.1)
Asp 48	7.7	6.3 (1.4)	6.8 (1.3)	6.3 (1.7)	5.8 (1.3)	6.6 (1.3)
Ser 50	7.8	*5.3* (1.4)	7.2 (1.6)	6.3 (1.8)	*5.4* (1.5)	7.7 (1.4)
Thr 51	9.8 (9.7)	8.3 (1.5)	9.2 (0.9)	8.2 (1.9)	8.8 (1.3)	9.1 (0.9)
Asp 52	9.6	8.0 (1.3)	8.5 (1.0)	*6.9* (1.4)	*6.8* (1.4)	8.6 (1.0)
Tyr 53	9.6	8.9 (1.0)	8.9 (1.0)	8.5 (1.3)	9.0 (0.9)	8.9 (1.0)
Leu 56	9.7	*7.3* (1.2)	9.0 (0.8)	7.7 (1.3)	8.7 (1.1)	9.2 (0.6)
Gln 57	6.3	6.8 (0.2)	6.2 (0.9)	6.6 (0.6)	5.2 (1.3)	6.6 (0.4)
Ile 58	8.0	8.0 (1.2)	7.9 (1.3)	8.3 (1.4)	8.1 (1.4)	7.3 (1.5)
Ser 60	5.1	5.2 (1.1)	4.7 (1.0)	4.6 (1.1)	4.3 (1.0)	4.7 (1.1)
Arg 61	6.2	5.7 (1.5)	7.1 (1.5)	4.7 (1.2)	5.3 (1.6)	5.5 (1.7)
Cys 64	8.8	7.5 (1.5)	9.3 (0.6)	7.8 (1.7)	8.1 (1.5)	8.9 (0.9)
Asn 65	9.4	*6.3* (1.3)	8.4 (1.1)	*7.2* (1.6)	*7.0* (1.5)	8.5 (1.0)
Asp 66	10.0 (9.7)	8.1 (1.6)	8.6 (1.3)	8.6 (1.2)	8.7 (1.2)	8.9 (1.0)
Arg 68	9.7	*6.9* (1.9)	8.6 (1.2)	7.7 (1.9)	7.9 (1.8)	9.1 (0.9)
Thr 69	9.3	*6.0* (1.9)	9.2 (0.6)	*5.4* (1.6)	*5.5* (1.5)	8.3 (1.2)
Cys 76	8.8	7.4 (1.5)	7.9 (1.2)	8.0 (1.3)	7.8 (1.4)	8.0 (1.3)
Asn 77	7.4	6.8 (0.2)	6.8 (0.2)	6.8 (0.2)	6.8 (0.2)	6.8 (0.2)
Ile 78	8.0	*4.7* (1.3)	8.6 (1.1)	*4.9* (1.8)	*4.3* (1.2)	7.5 (1.5)
Cys 80	3.6	4.5 (0.9)	4.4 (1.0)	4.5 (0.9)	4.7 (0.9)	4.5 (1.0)
Ser 81	3.6	4.0 (0.9)	4.0 (0.9)	4.0 (0.9)	3.9 (0.9)	3.9 (0.9)
Ala 82	5.4	4.7 (1.0)	4.7 (1.0)	4.8 (1.1)	4.6 (1.0)	4.7 (1.0)
Leu 83	7.2	5.3 (1.2)	6.1 (1.2)	*5.1* (1.2)	*5.1* (1.1)	6.4 (1.2)
Leu 84	9.2	*5.6* (1.3)	8.5 (0.8)	*6.2* (1.4)	*5.0* (1.3)	8.3 (1.1)
Ser 85	5.8	6.3 (1.9)	6.6 (1.9)	5.7 (2.0)	6.3 (1.6)	6.0 (2.0)
Ser 86	5.8	4.9 (1.2)	4.9 (1.3)	4.8 (1.4)	6.1 (1.2)	4.9 (1.3)
Asp 87	8.9	*6.5* (1.9)	8.9 (1.0)	7.4 (2.0)	*6.3* (1.6)	8.5 (1.2)
Ile 88	6.5	7.3 (1.8)	6.9 (1.4)	6.9 (1.5)	5.6 (1.9)	7.1 (1.5)
Ala 90	4.2	4.5 (1.0)	4.9 (1.1)	4.6 (1.0)	4.3 (0.9)	4.4 (1.0)
Ser 91	5.5	4.2 (1.0)	4.5 (0.9)	4.3 (0.9)	4.4 (1.0)	4.6 (1.1)
Val 92	5.6	4.8 (1.0)	4.8 (0.9)	4.7 (0.9)	5.0 (1.0)	5.1 (1.0)
Asn 93	4.4	4.5 (0.9)	4.4 (0.9)	4.5 (0.9)	4.5 (0.9)	4.6 (1.0)
Cys 94	6.3	5.1 (1.0)	5.1 (1.0)	5.3 (1.0)	5.6 (1.1)	5.1 (1.1)
Lys 96	4.4	4.3 (0.9)	4.4 (1.0)	4.2 (0.9)	4.4 (0.9)	4.1 (0.9)
Lys 97	6.5	4.7 (1.0)	6.2 (1.4)	4.9 (1.2)	4.8 (1.1)	5.5 (1.2)
Val 99	5.2	5.9 (1.2)	5.0 (1.4)	5.3 (1.1)	6.1 (1.6)	5.2 (1.3)
Asp 101	7.0	6.1 (1.5)	6.5 (1.8)	6.5 (1.7)	6.0 (1.7)	7.1 (1.9)
Asn 103	8.2	*5.4* (1.4)	8.5 (1.2)	*5.6* (1.8)	*5.9* (2.1)	8.1 (1.5)
Met 105	7.4	7.0 (1.4)	6.7 (1.5)	*4.7* (1.8)	6.5 (1.5)	6.9 (1.2)
Ala 107	4.2	5.2 (1.3)	4.7 (1.4)	*6.3* (2.0)	5.7 (1.7)	4.5 (1.2)
Trp 108	9.6	*5.9* (1.3)	8.6 (1.1)	*5.7* (1.5)	*6.5* (1.8)	8.7 (1.2)
Val 109	4.0	4.7 (1.0)	4.4 (1.1)	4.7 (1.2)	*6.7* (2.2)	4.6 (1.2)
Trp 111	7.1	5.4 (1.2)	6.3 (1.1)	5.2 (1.4)	6.3 (2.5)	6.4 (1.2)
Arg 112	4.5	4.1 (1.1)	4.4 (1.1)	5.2 (1.6)	4.4 (1.5)	4.3 (1.1)
Asn 113	5.8	7.3 (1.8)	5.9 (1.2)	7.6 (2.0)	6.2 (2.0)	6.2 (1.4)
Arg 114	9.6	*7.3* (1.9)	9.2 (0.8)	*6.2* (1.8)	*6.6* (1.9)	9.1 (0.8)
Cys 115	9.8 (9.7)	*6.2* (1.7)	9.2 (0.7)	*6.4* (1.6)	*6.7* (1.7)	9.0 (0.8)
Thr 118	9.8 (9.7)	*6.8* (1.9)	8.7 (1.2)	*6.7* (1.9)	*6.8* (1.9)	8.8 (1.1)
Asp 119	6.7	5.9 (1.8)	6.9 (1.8)	6.9 (2.1)	6.0 (1.8)	6.4 (1.7)
Val 120	4.6	6.4 (2.2)	4.4 (1.1)	4.7 (1.3)	4.5 (1.3)	4.7 (1.2)
Gln 121	5.0	4.2 (1.2)	5.2 (1.4)	5.2 (1.1)	5.1 (1.3)	5.6 (1.3)
Ala 122	3.7	4.9 (1.3)	4.5 (1.2)	4.8 (1.2)	5.0 (1.3)	4.2 (1.2)
Trp 123	5.4	6.0 (1.6)	5.3 (1.5)	5.4 (1.4)	5.2 (1.4)	5.1 (1.4)
Ile 124	10.6 (9.7)	*6.7* (1.6)	8.5 (1.2)	*5.9* (1.4)	*5.5* (1.5)	8.7 (1.0)
Arg 125	4.4	5.9 (1.9)	5.3 (1.4)	*6.5* (1.9)	*6.5* (1.9)	5.1 (1.4)
Cys 127	7.7	*5.6* (1.8)	6.9 (1.9)	*5.3* (1.8)	7.0 (1.9)	6.9 (1.7)
Arg 128	8.0	6.8 (1.9)	7.7 (1.7)	6.7 (1.7)	6.9 (1.6)	7.8 (1.6)
Leu 129	9.0	7.5 (1.8)	8.5 (1.4)	7.7 (1.8)	7.8 (1.8)	8.1 (1.6)

Experimental values from Table II of Smith et al. (1991). The value within brackets in the column “Experimental value” represents the maximum in the Karplus relation (Pardi et al. 1984) used for the calculation of the ^3^*J*_*HNHα*_-couplings. The root-mean-square fluctuations (RMSF) of the ^3^*J*-couplings in the MD simulations are given within parentheses. MD values differing more than 2 Hz from the experimental value and the maximum of the Karplus curve are denoted using italics

Table 6 lists 22 experimentally stereo-specifically unassigned backbone ^3^*J*_*HNHα*_-coupling values derived from NMR measurements (set *bb2*) and values calculated from the unrestrained and ^3^*J*-coupling time-averaging local-elevation restrained MD simulations starting from the *2VB1* X-ray crystal structure using different sets of backbone and side-chain restraints. The unrestrained MD simulation shows one ^3^*J*_*HNHα*_-coupling value (in italics), for the α2 *Re* hydrogen in residue Gly 49, that deviates more than 2 Hz from the experimental value. ^3^*J*_*HNHα*_-coupling time-averaging local-elevation restraining towards the sets *bb1* and *bb2* of 95 and 22 target backbone ^3^*J*_*HNHα*_-coupling values leads, as expected, to good agreement between simulation and experiment for the 22 backbone ^3^*J*_*HNHα*_-couplings. No deviations larger than 2 Hz are observed.

**Table 6:** Experimentally stereo-specifically unassigned backbone ^3^*J*_*HNHα*_-coupling values (22) in Hz derived from NMR measurements (set *bb2*) and values from the unrestrained and ^3^*J*-coupling time-averaging local-elevation restrained MD simulations starting from the *2VB1* X-ray crystal structure and using different sets of backbone and side-chain restraints

Residue	Experimental value	Unrestrained MD	^3^*J*-coupling local-elevation restrained MD			
		*2VB1*	*2VB1*_*bb1* + *bb2*	*2VB1*_*sc1*	*2VB1*_*sc1* + *sc2*	*2VB1*_*bb1* + *bb2* + *sc1* + *sc2*
Gly 4						
α2 Re	8.0	7.5 (1.8)	7.3 (1.5)	7.6 (1.6)	6.3 (1.9)	7.4 (1.6)
α3 Si	6.1	4.9 (1.7)	5.4 (1.3)	5.1 (1.4)	5.9 (1.4)	5.3 (1.3)
Gly 16						
α2 Re	6.1	5.3 (1.7)	5.9 (1.3)	5.8 (1.3)	6.2 (1.1)	5.5 (1.5)
α3 Si	6.2	6.3 (2.0)	6.1 (1.9)	6.2 (1.8)	5.3 (1.6)	5.7 (2.0)
Gly 22						
α2 Re	6.0	6.1 (0.9)	5.9 (1.2)	6.0 (0.9)	5.8 (1.2)	5.9 (1.1)
α3 Si	6.8	6.4 (1.6)	6.4 (1.6)	6.5 (1.6)	6.7 (1.7)	6.7 (1.7)
Gly 26						
α2 Re	3.3	3.9 (1.0)	4.1 (1.0)	4.0 (0.9)	4.2 (1.0)	4.0 (1.0)
α3 Si	6.2	6.7 (0.3)	6.8 (0.2)	6.8 (0.2)	6.8 (0.2)	6.7 (0.2)
Gly 49						
α2 Re	5.3	*3.1* (1.2)	4.6 (1.6)	4.4 (1.8)	3.4 (1.4)	5.0 (1.4)
α3 Si	8.0	9.1 (1.0)	8.1 (1.6)	7.3 (1.9)	8.9 (1.2)	7.8 (1.7)
Gly 67						
α2 Re	5.5	6.0 (1.6)	5.6 (1.5)	5.9 (1.4)	6.0 (1.2)	6.2 (1.0)
α3 Si	6.2	6.4 (1.4)	6.4 (1.0)	6.6 (1.6)	6.3 (1.8)	6.0 (1.7)
Gly 71						
α2 Re	5.9	6.2 (1.1)	5.7 (1.6)	6.7 (0.4)	6.6 (0.6)	6.6 (0.6)
α3 Si	5.7	5.5 (1.8)	6.2 (2.0)	4.8 (1.3)	5.1 (1.4)	5.2 (1.4)
Gly 102						
α2 Re	6.2	4.2 (1.1)	5.2 (1.4)	4.2 (1.7)	5.6 (1.8)	5.9 (2.0)
α3 Si	6.4	6.7 (0.3)	6.6 (0.5)	7.2 (2.0)	4.8 (2.0)	5.9 (1.9)
Gly 104						
α2 Re	6.4	6.7 (0.4)	5.7 (1.6)	5.8 (1.5)	5.9 (1.8)	6.4 (1.9)
α3 Si	3.9	4.5 (1.2)	4.6 (1.8)	5.5 (1.8)	4.8 (2.0)	4.5 (1.7)
Gly 117						
α2 Re	6.2	5.3 (1.6)	6.5 (1.5)	6.1 (1.5)	6.3 (1.5)	5.8 (1.6)
α3 Si	6.5	6.1 (1.3)	5.7 (1.8)	5.1 (2.0)	5.4 (1.7)	6.4 (1.8)
Gly 126						
α2 Re	6.8	6.5 (1.1)	6.5 (1.4)	6.2 (1.3)	6.1 (1.8)	6.4 (1.1)
α3 Si	5.6	5.3 (1.8)	5.8 (1.5)	5.9 (1.9)	5.5 (2.2)	5.7 (1.7)

Experimental values from Table II of Smith et al. (1991). The root-mean-square fluctuations (RMSF) of the ^3^*J*-couplings in the MD simulations are given within parentheses. Stereo-specific assignments for restraining were based on the ^3^*J*_*HN-Hα*_-coupling values calculated from the four unrestrained MD simulations starting from the four X-ray structures in case 4 or 3 of the unrestrained MD simulations suggested the same stereo-specific assignment. Only Gly 104 could not be stereo-specifically assigned using this criterion. For this residue the stereo-specific assignment was based on the ^3^*J*_*HN-Hα*_-coupling values calculated from the four X-ray structures using a corresponding criterion. MD values differing more than 2 Hz from the experimental value and the maximum of the Karplus curve are denoted using italics

Table 7 lists 58 side-chain ^3^*J*_*HαHβ*_-coupling values in Hz derived and stereo-specifically assigned based on NMR measurements (set *sc1*) and from the unrestrained and ^3^*J*-coupling time-averaging local-elevation restrained MD simulations starting from the *2VB1* X-ray crystal structure using different sets of backbone and side-chain restraints. The unrestrained MD simulation shows 14 ^3^*J*_*HαHβ*_-coupling values (in italics) that deviate more than 2 Hz from the experimental values (residues 18, 20(2), 27, 30, 46(2), 51, 59, 61, 66, 69, 89, 99). Restraining towards the sets *bb1* and *bb2* of 95 and 22 backbone ^3^*J*_*HNHα*_-coupling values yields 18 deviations larger than 2 Hz, and so no improvement of the agreement between simulated and experimental ^3^*J*_*HαHβ*_-coupling values, as one would expect. Backbone ^3^*J*_*HNHα*_-coupling restraining does not improve the agreement between simulation and experiment for the side-chain ^3^*J*_*HαHβ*_-couplings. ^3^*J*_*HαHβ*_-coupling time-averaging local-elevation restraining towards the sets *sc1* or *sc1* and *sc2* of 58 and 38 target side-chain ^3^*J*_*HαHβ*_-coupling values leads, as expected, to good agreement between simulation and experiment for the 58 side-chain ^3^*J*_*HαHβ*_-couplings. No deviations larger than 2 Hz are observed. This is also the case when restraining towards all 213 experimental backbone and side-chain ^3^*J-*coupling values (sets *bb1*, *bb2*, *sc1* and *sc2*).

**Table 7:** Side-chain ^3^*J*_*HαHβ*_-coupling values (58) in Hz derived and stereo-specifically assigned based on NMR measurements (set *sc1*) and from the unrestrained and ^3^*J*-coupling time-averaging local-elevation restrained MD simulations starting from the *2VB1* X-ray crystal structure and using different sets of backbone and side-chain restraints

Residue	Experimental value	Unrestrained MD	^3^*J*-coupling local-elevation restrained MD			
		*2VB1*	*2VB1*_*bb1* + *bb2*	*2VB1*_*sc1*	*2VB1*_*sc1* + *sc2*	*2VB1*_*bb1* + *bb2* + *sc1* + *sc2*
Val 2	10.8	9.3 (4.6)	*7.8* (4.9)	11.9 (1.9)	11.5 (2.8)	11.6 (2.6)
Cys 6 β_2_	11.5	12.6 (0.6)	12. 6 (0.4)	12.5 (1.0)	10.7 (1.9)	12.5 (0.7)
β_3_	3.5	3.4 (1.1)	3.3 (1.0)	3.2 (1.2)	2.8 (1.7)	3.0 (1.0)
His 15 β_2_	11.2	11.9 (1.3)	12.4 (0.7)	12.4 (0.7)	12.2 (1.0)	12.4 (0.6)
β_3_	2.6	2.4 (0.9)	2.7 (0.8)	2.8 (0.9)	2.7 (0.9)	2.9 (1.0)
Asp 18 β_2_	4.2	5.1 (4.1)	4.6 (4.1)	3.1 (1.3)	3.0 (1.5)	3.1 (1.2)
β_3_	11.0	*7.5* (4.3)	*8.8* (3.9)	11.9 (1.9)	11.8 (1.9)	12.1 (1.1)
Tyr 20 β_2_	2.3	*7.2* (4.8)	*9.9* (4.1)	2.7 (1.5)	2.4 (0.7)	2.5 (1.0)
β_3_	11.7	*6.9* (4.5)	*4.8* (3.7)	11.9 (1.8)	12.2 (0.8)	12.0 (1.5)
Tyr 23 β_2_	10.9	12.6 (0.5)	12.3 (1.0)	11.9 (1.4)	11.5 (1.6)	11.6 (1.5)
β_3_	2.7	3.1 (1.0)	3.2 (1.2)	3.0 (1.4)	2.8 (1.3)	2.8 (1.3)
Asn 27 β_2_	10.3	*3.6* (2.9)	11.9 (2.1)	11.0 (1.9)	11.1 (1.8)	10.7 (1.7)
β_3_	2.4	4.3 (1.2)	3.7 (1.6)	2.5 (1.5)	2.5 (1.5)	2.1 (1.0)
Val 29	11.1	10.1 (4.3)	10.3 (4.1)	12.0 (2.1)	12.1 (1.9)	12.2 (1.6)
Cys 30 β_2_	5.3	*3.2* (1.0)	*3.0* (1.1)	5.4 (1.7)	5.4 (1.8)	4.7 (1.7)
β_3_	10.8	12.6 (0.6)	12.5 (0.9)	11.7 (1.4)	11.3 (2.3)	11.8 (1.9)
Phe 34 β_2_	10.7	10.7 (3.3)	11.9 (1.4)	11.3 (2.7)	10.5 (2.6)	11.7 (2.1)
β_3_	5.0	3.5 (3.1)	*2.5* (1.1)	4.3 (2.0)	4.8 (2.2)	4.1 (1.8)
Asn 39 β_2_	4.5	2.5 (1.0)	2.6 (0.9)	3.3 (1.6)	3.7 (1.8)	3.5 (1.4)
β_3_	10.8	12.0 (1.1)	12.0 (1.1)	11.3 (2.3)	11.3 (2.3)	12.0 (1.2)
Thr 40	4.5	2.7 (0.8)	3.0 (0.9)	3.4 (1.0)	3.3 (1.0)	3.3 (1.2)
Thr 43	3.7	3.4 (2.6)	3.0 (1.8)	2.9 (1.5)	3.0 (1.6)	2.9 (1.5)
Asn 46 β_2_	11.2	*2.6* (2.0)	*8.9* (5.0)	11.5 (1.9)	12.2 (1.5)	12.3 (1.3)
β_3_	4.7	*9.2* (1.9)	5.8 (3.5)	4.1 (1.7)	3.8 (1.4)	3.7 (1.5)
Thr 47	2.6	3.0 (1.5)	2.9 (1.2)	2.9 (1.4)	2.9 (1.4)	2.9 (1.4)
Asp 48 β_2_	2.6	4.2 (1.1)	3.9 (1.2)	3.5 (1.4)	3.5 (1.1)	3.5 (1.2)
β_3_	3.7	2.9 (1.0)	3.2 (1.1)	3.7 (1.4)	3.5 (1.1)	3.6 (1.1)
Thr 51	9.3	*5.6* (4.6)	9.5 (4.3)	10.0 (1.9)	9.6 (3.1)	10.1 (2.3)
Asp 52 β_2_	11.6	12.6 (0.4)	12.5 (0.7)	12.4 (1.1)	12.4 (0.8)	12.5 (0.7)
β_3_	3.6	3.8 (1.1)	3.8 (1.3)	3.8 (1.2)	3.1 (1.2)	4.1 (1.3)
Tyr 53 β_2_	10.4	12.1 (0.9)	12.4 (0.7)	11.3 (1.3)	11.6 (1.1)	11.5 (1.1)
β_3_	3.0	2.4 (0.7)	2.6 (0.8)	2.2 (0.7)	2.5 (1.2)	2.3 (0.9)
Asn 59 β_2_	5.4	*2.3* (0.5)	*2.2* (0.5)	4.6 (1.0)	4.9 (0.9)	4.3 (0.9)
β_3_	11.3	12.3 (0.6)	12.1 (0.7)	12.1 (1.5)	12.2 (1.4)	12.4 (1.4)
Arg 61 β_2_	5.7	5.5 (4.3)	*9.5* (4.3)	4.8 (1.8)	4.9 (1.4)	4.6 (1.5)
β_3_	10.8	*8.4* (4.6)	*5.1* (4.0)	11.5 (2.2)	11.5 (2.3)	10.8 (3.1)
Asp 66 β_2_	5.1	3.2 (1.2)	3.1 (1.3)	4.6 (2.1)	4.7 (2.1)	4.2 (1.9)
β_3_	4.5	*11.5* (2.9)	*10.6* (3.8)	3.4 (1.8)	3.6 (2.1)	3.5 (1.7)
Thr 69	9.3	*6.1* (4.6)	*6.9* (4.6)	9.9 (1.9)	10.3 (1.3)	10.2 (1.8)
Leu 75 β_2_	12.4	11.5 (2.4)	*8.2* (4.6)	11.9 (1.6)	11.9 (1.6)	11.7 (2.0)
β_3_	2.1	3.0 (1.8)	*6.1* (4.6)	2.7 (1.2)	2.6 (1.0)	2.9 (1.6)
Asp 87 β_2_	5.1	3.3 (1.3)	*2.7* (1.0)	4.2 (1.4)	4.1 (1.1)	4.4 (1.4)
β_3_	11.5	12.2 (1.1)	12.0 (1.1)	12.0 (1.6)	12.3 (1.4)	12.2 (1.7)
Ile 88	4.5	4.3 (3.8)	2.8 (0.8)	3.5 (1.9)	3.5 (2.0)	3.8 (2.3)
Thr 89	9.5	*4.8* (3.4)	*3.5* (2.8)	10.1 (2.3)	10.2 (1.7)	10.4 (1.7)
Val 92	10.1	9.6 (4.5)	*12.2* (1.9)	11.0 (2.0)	10.7 (2.3)	10.9 (2.4)
Cys 94 β_2_	4.0	2.6 (0.7)	2.8 (1.5)	2.8 (1.1)	2.7 (0.8)	3.1 (0.9)
β_3_	12.2	12.4 (0.6)	12.1 (1.6)	12.4 (1.0)	12.4 (0.8)	12.5 (0.9)
Val 99	6.3	*3.0* (1.6)	*3.2* (2.2)	5.3 (2.0)	5.6 (2.7)	5.3 (2.1)
Val 109	8.0	9.0 (4.7)	9.3 (4.7)	7.5 (3.9)	8.3 (3.8)	8.3 (3.7)
Thr 118	4.2	2.9 (1.1)	3.3 (2.3)	3.2 (2.1)	3.5 (2.4)	3.6 (0.6)
Asp 119 β_2_	4.9	4.5 (3.8)	3.0 (1.2)	4.3 (1.5)	4.2 (1.7)	3.9 (1.6)
β_3_	11.7	10.1 (3.8)	12.1 (1.7)	12.1 (1.8)	11.7 (2.5)	12.3 (1.5)
Trp 123 β_2_	10.6	12.2 (1.2)	9.7 (2.8)	10.4 (2.0)	10.6 (2.4)	10.7 (1.8)
β_3_	2.9	3.7 (1.6)	2.9 (1.9)	2.4 (1.6)	2.7 (1.8)	2.3 (1.2)
Ile 124	4.6	4.1 (2.7)	3.8 (2.0)	4.1 (2.1)	3.9 (2.1)	3.9 (1.6)
Cys 127 β_2_	11.6	12.6 (0.6)	12.5 (0.7)	12.2 (1.7)	12.1 (1.9)	12.5 (0.5)
β_3_	4.8	3.2 (1.0)	3.4 (1.2)	3.9 (1.5)	3.9 (1.6)	4.1 (1.2)

Experimental values from Tables III and IV of Smith et al. (1991). The root-mean-square fluctuations (RMSF) of the ^3^*J*-couplings in the MD simulations are given within parentheses. MD values differing more than 2 Hz from the experimental value are denoted using italics

Table 8 lists 40 experimentally stereo-specifically unassigned side-chain ^3^*J*_*HαHβ*_-coupling values derived from NMR measurements (set *sc2* plus Glu 7) and values from the unrestrained and ^3^*J*-coupling time-averaging local-elevation restrained MD simulations starting from the *2VB1* X-ray crystal structure using different sets of backbone and side-chain restraints. The unrestrained MD simulation shows 13 ^3^*J*_*HαHβ*_-coupling values (in italics) that deviate more than 2 Hz from the experimental values (residues 68, 72(2), 77(2), 85, 86, 101(2), 106, 125(2), 128). Restraining towards the sets *bb1* and *bb2* of 95 and 22 backbone ^3^*J*_*HNHα*_-coupling values yields 13 deviations larger than 2 Hz, and so no improvement of the agreement between simulated and experimental ^3^*J*_*HαHβ*_-coupling values, as one would expect. Backbone ^3^*J*_*HNHα*_-coupling restraining does not improve the agreement between simulation and experiment for the side-chain ^3^*J*_*HαHβ*_-couplings, apart from a few cases. This is also observed using the *sc1* set of 58 restraints (14 deviations larger than 2 Hz). ^3^*J*_*HαHβ*_-coupling time-averaging local-elevation restraining towards the sets *sc1* and *sc2* of 58 and 38 target side-chain ^3^*J*_*HαHβ*_-coupling values leads, as expected, to good agreement between simulation and experiment for the 40 side-chain ^3^*J*_*HαHβ*_-couplings. Only two deviations (Glu 7 β_2_ and β_3_) larger than 2 Hz are observed. This is also the case when restraining towards all 213 experimental backbone and side-chain ^3^*J-*coupling values (sets *bb1*, *bb2*, *sc1* and *sc2*), only for Glu 7 β_2_ the deviation is larger than 2 Hz. Note that Glu 7 is not part of the set *sc2* of restraints.

**Table 8:** Experimentally stereo-specifically unassigned side-chain ^3^*J*_*HαHβ*_-coupling values (40) in Hz derived from NMR measurements (set *sc2* plus Glu 7) and values from the unrestrained and ^3^*J*-coupling time-averaging local-elevation restrained MD simulations starting from the *2VB1* X-ray crystal structure and using different sets of backbone and side-chain restraints

Residue	Experimental value	Unrestrained MD	^3^*J*-coupling local-elevation restrained MD			
		*2VB1*	*2VB1*_*bb1* + *bb2*	*2VB1*_ *sc1*	*2VB1*_ *sc1* + *sc2*	*2VB1*_*bb1* + *bb2* + *sc1* + *sc2*
Glu 7						
β_2_	6.7*	7.8 (4.7)	*9.7* (4.3)	*11.5* (2.8)	*11.5* (2.9)	*9.1* (4.6)
β_3_	6.4*	7.0 (4.6)	5.6 (4.1)	*4.1* (2.6)	*3.6* (2.3)	5.9 (4.2)
Lys 13						
β_2_	5.1	5.7 (4.5)	*7.7* (4.7)	6.8 (4.5)	4.7 (3.3)	4.4 (2.6)
β_3_	9.2	8.6 (4.7)	*6.5* (4.8)	7.2 (4.9)	8.8 (3.2)	9.2 (3.5)
Asn 19						
β_2_	7.3	8.3 (4.6)	8.0 (4.7)	7.3 (3.8)	6.7 (3.0)	6.9 (2.5)
β_3_	6.4	5.9 (4.6)	5.7 (4.4)	*3.3* (2.8)	6.0 (3.3)	5.9 (3.2)
Trp 28						
β_2_	10.7	12.6 (0.5)	12.4 (0.7)	12.5 (0.5)	11.8 (1.3)	11.9 (1.4)
β_3_	4.1	3.9 (1.2)	4.2 (1.5)	3.0 (1.0)	3.0 (1.5)	4.5 (1.8)
Asn 37						
β_2_	8.1	9.1 (4.6)	6.2 (4.7)	*4.7* (4.1)	8.4 (3.0)	8.2 (3.4)
β_3_	4.2	5.1 (3.9)	*7.9* (4.6)	*9.6* (4.1)	3.6 (2.5)	3.8 (2.2)
Arg 45						
β_2_	6.9	8.0 (4.8)	8.9 (4.6)	*9.0* (4.5)	6.6 (3.1)	6.8 (3.8)
β_3_	6.7	6.4 (4.6)	5.4 (4.2)	5.0 (3.9)	6.2 (3.3)	5.9 (2.7)
Cys 64						
β_2_	4.6	4.4 (1.0)	4.0 (0.9)	4.7 (1.0)	4.5 (1.0)	4.5 (1.0)
β_3_	2.7	2.7 (0.7)	2.9 (0.7)	2.5 (0.6)	2.6 (0.6)	2.6 (0.6)
Asn 65						
β_2_	4.5	4.2 (3.1)	2.9 (1.3)	3.5 (2.4)	3.5 (1.6)	3.6 (1.7)
β_3_	11.4	11.3 (3.2)	10.1 (3.9)	11.9 (2.2)	12.2 (1.6)	12.1 (1.6)
Arg 68						
β_2_	6.5	*10.1* (4.1)	*10.3* (3.9)	*9.2* (4.5)	6.3 (3.0)	6.9 (2.8)
β_3_	4.8	4.5 (3.6)	4.3 (3.4)	5.2 (4.0)	4.6 (3.0)	4.3 (3.0)
Ser 72						
β_2_	5.4	*7.5* (4.7)	3.6 (2.4)	3.9 (3.0)	4.9 (2.8)	5.1 (3.7)
β_3_	7.6	*5.2* (3.6)	*11.6* (2.7)	7.5 (4.3)	7.3 (2.9)	7.1 (2.3)
Asn 74						
β_2_	10.5	11.3 (3.2)	*2.5* (1.6)	9.8 (4.5)	11.6 (1.2)	11.6 (1.8)
β_3_	3.9	4.0 (2.2)	*11.7* (1.7)	5.7 (3.7)	3.9 (2.0)	3.8 (2.1)
Asn 77						
β_2_	8.3	*10.8* (3.4)	*11.2* (3.3)	*11.4* (3.0)	8.4 (3.5)	8.6 (3.0)
β_3_	5.9	*3.8* (2.6)	3.9 (2.7)	3.9 (2.7)	5.4 (3.1)	5.3 (2.7)
Ser 85						
β_2_	5.7	4.9 (3.9)	4.5 (3.6)	4.9 (4.0)	5.6 (4.1)	5.7 (3.7)
β_3_	7.4	*9.6* (4.3)	9.0 (4.3)	8.5 (4.4)	6.8 (3.1)	7.3 (3.4)
Ser 86						
β_2_	6.4	*8.7* (4.6)	7.6 (4.7)	*8.5* (4.6)	6.2 (2.9)	6.3 (2.8)
β_3_	4.1	3.9 (2.1)	4.2 (2.6)	4.4 (3.0)	3.4 (2.5)	3.7 (2.8)
Asn 93						
β_2_	10.8	10.7 (3.6)	11.1 (3.1)	10.1 (4.0)	12.0 (1.0)	11.9 (1.5)
β_3_	3.5	4.1 (3.6)	3.7 (3.1)	4.6 (4.0)	2.4 (0.8)	2.6 (1.3)
Ser 100						
β_2_	7.7	7.7 (4.6)	7.9 (4.7)	8.8 (4.6)	8.0 (2.5)	8.1 (2.5)
β_3_	4.0	5.2 (4.0)	4.4 (2.8)	5.0 (3.8)	3.7 (2.7)	3.6 (2.2)
Asp 101						
β_2_	5.6	*2.5* (0.8)	*3.2* (2.4)	3.8 (3.0)	5.3 (2.8)	5.6 (2.8)
β_3_	6.6	*12.2* (0.9)	*11.3* (2.8)	*11.0* (3.3)	5.8 (2.1)	5.9 (2.2)
Asn 106						
β_2_	10.5	*4.7* (2.8)	*7.8* (4.6)	*4.4* (3.3)	11.5 (2.0)	11.1 (2.3)
β_3_	3.6	3.9 (2.7)	5.2 (3.9)	4.8 (3.0)	3.0 (1.6)	3.1 (1.9)
Arg 125						
β_2_	7.9	*10.4* (3.7)	9.6 (4.1)	*11.2* (3.0)	7.8 (2.9)	7.6 (3.0)
β_3_	6.1	*4.0* (3.3)	4.3 (3.7)	*3.3* (2.3)	5.4 (3.3)	5.7 (3.2)
Arg 128						
β_2_	7.9	9.3 (4.4)	9.3 (4.4)	9.7 (4.1)	7.8 (2.9)	7.8 (3.6)
β_3_	7.2	*4.8* (3.9)	*5.0* (4.0)	*4.2* (3.5)	7.0 (3.2)	6.7 (3.1)

Experimental values from Table III of Smith et al. (1991). The root-mean-square fluctuations (RMSF) of the ^3^*J*-couplings in the MD simulations are given within parentheses. Stereo-specific assignments for restraining were based on the ^3^*J*_*Hα-Hβ*_-coupling values calculated from the four unrestrained MD simulations starting from the four X-ray structures in case 4 or 3 of the unrestrained MD simulations suggested the same stereo-specific assignment. *Only Glu 7 could not be stereo-specifically assigned using this criterion. MD values differing more than 2 Hz from the experimental value are denoted using italics

Tables 9, 10, 11 and 12 summarise the agreement between experimental ^3^*J-*coupling values and those calculated from the four X-ray structures, from the four unrestrained MD simulation trajectories and from the ^3^*J*-coupling time-averaging local-elevation restrained MD simulation trajectories. Using backbone ^3^*J*_*HNHα*_-coupling restraints (sets *bb1* and *bb2*) only, the experimental ^3^*J*_*HNHα*_-coupling values are reproduced and using ^3^*J*_*HαHβ*_-coupling restraints (*sc1* or *sc1* and *sc2*) only, the experimental ^3^*J*_*HαHβ*_-coupling values are reproduced. Not surprisingly, there appears to be insignificant mutual influence between the different sets of restraints. Using all 213 experimental backbone and side-chain ^3^*J-*coupling restraints (sets *bb1*, *bb2*, *sc1* and *sc2*) no significant deviations from experimental ^3^*J-*coupling values are observed.

**Table 9:** Number of deviations, |^3^*J*_*HNHα*_ (exp) − ^3^*J*_*HNHα*_ (MD or X-ray)|, for the 95 backbone ^3^*J*_*HNHα*_-coupling values derived and assigned based on NMR measurements (set *bb1*), in four X-ray crystal structures, in the four unrestrained MD simulations starting from these, and in the ^3^*J*-coupling time-averaging local-elevation restrained MD simulations starting from the *2VB1* X-ray crystal structure and using different sets of backbone and side-chain restraints

Crystal structure or simulation	Size of ^3^*J*_*HNHα*_ deviation (in Hz)				
	1–2	2–3	3–4	4–5	> 5
*X-ray_2VB1*	13	2	1	0	0
*X-ray_4LZT*	12	2	0	0	0
*X-ray_1IEE*	25	3	1	0	0
*X-ray_1AKI*	18	1	0	0	0
*MD_2VB1*	25	10	8	0	0
*MD_4LZT*	27	12	4	1	0
*MD_1IEE*	30	7	6	0	0
*MD_1AKI*	25	10	7	0	0
*MD_2VB1_bb1* + *bb2*	7	0	0	0	0
*MD_2VB1_sc1*	29	12	6	0	0
*MD_2VB1_sc1* + *sc2*	21	14	4	2	0
*MD_2VB1_bb1* + *bb2* + *sc1* + *sc2*	2	0	0	0	0

**Table 10:** Number of deviations, |^3^*J*_*HNHα*_ (exp) − ^3^*J*_*HNHα*_ (MD or X-ray)|, for the 22 backbone ^3^*J*_*HNHα*_-coupling values derived but stereo-specifically unassigned from NMR measurements (set *bb2*), in four X-ray crystal structures, in the four unrestrained MD simulations starting from these, and in the ^3^*J*-coupling time-averaging local-elevation restrained MD simulations starting from the *2VB1* X-ray crystal structure and using different sets of backbone and side-chain restraints

Crystal structure or simulation	Size of ^3^*J*_*HNHα*_ deviation (in Hz)				
	1–2	2–3	3–4	4–5	> 5
*X-ray_2VB1*	5	0	0	0	0
*X-ray_4LZT*	4	1	0	0	0
*X-ray_1IEE*	3	3	0	1	0
*X-ray_1AKI*	2	1	1	1	0
*MD_2VB1*	3	1	0	0	0
*MD_4LZT*	6	0	0	0	0
*MD_1IEE*	3	0	0	0	0
*MD_1AKI*	3	0	0	0	0
*MD_2VB1_bb1* + *bb2*	0	0	0	0	0
*MD_2VB1_sc1*	3	0	0	0	0
*MD_2VB1_sc1* + *sc2*	4	0	0	0	0
*MD_2VB1_bb1* + *bb2* + *sc1* + *sc2*	0	0	0	0	0

Stereo-specific assignments for restraining were based on the ^3^*J*_*HN-Hα*_-coupling values calculated from the four unrestrained MD simulations starting from the four X-ray structures in case 4 or 3 of the unrestrained MD simulations suggested the same stereo-specific assignment. Only Gly 104 could not be stereo-specifically assigned using this criterion. For this residue the stereo-specific assignment was based on the ^3^*J*_*HN-Hα*_-coupling values calculated from the four X-ray structures using a corresponding criterion

**Table 11:** Number of deviations, |^3^*J*_*HαHβ*_ (exp) − ^3^*J*_*HαHβ*_ (MD or X-ray)|, for the 58 side-chain ^3^*J*_*HαHβ*_-coupling values derived and stereo-specifically assigned based on NMR measurements (set *sc1*), in four X-ray crystal structures, in the four unrestrained MD simulations starting from these, and in the ^3^*J*-coupling time-averaging local-elevation restrained MD simulations starting from the *2VB1* X-ray crystal structure and using different sets of backbone and side-chain restraints

Crystal structure or simulation	Size of ^3^*J*_*HαHβ*_ deviation (in Hz)				
	1–2	2–3	3–4	4–5	> 5
*X-ray_2VB1*	23	9	4	1	1
*X-ray_4LZT*	23	8	4	1	1
*X-ray_1IEE*	23	9	4	2	0
*X-ray_1AKI*	22	12	5	1	0
*MD_2VB1*	18	2	5	4	3
*MD_4LZT*	18	7	5	0	2
*MD_1IEE*	15	9	1	1	4
*MD_1AKI*	18	3	4	1	7
*MD_2VB1_bb1* + *bb2*	19	8	4	1	5
*MD_2VB1_sc1*	7	0	0	0	0
*MD_2VB1_sc1* + *sc2*	4	0	0	0	0
*MD_2VB1_bb1* + *bb2* + *sc1* + *sc2*	11	0	0	0	0

**Table 12:** Number of deviations, |^3^*J*_*HαHβ*_ (exp) − ^3^*J*_*HαHβ*_ (MD or X-ray)|, for the 38 side-chain ^3^*J*_*HαHβ*_-coupling values derived but stereo-specifically unassigned from NMR measurements (set *sc2*), in four X-ray crystal structures, in the four unrestrained MD simulations starting from these, and in the ^3^*J*-coupling time-averaging local-elevation restrained MD simulations starting from the *2VB1* X-ray crystal structure and using different sets of backbone and side-chain restraints

Crystal structure or simulation	Size of ^3^*J*_*HαHβ*_ deviation (in Hz)				
	1–2	2–3	3–4	4–5	> 5
*X-ray_2VB1*	9	3	4	5	13
*X-ray_4LZT*	10	4	3	4	13
*X-ray_1IEE*	9	5	4	2	13
*X-ray_1AKI*	11	5	6	1	11
*MD_2VB1*	4	9	2	0	2
*MD_4LZT*	10	7	0	2	1
*MD_1IEE*	18	5	2	4	0
*MD_1AKI*	16	3	4	2	1
*MD_2VB1_bb1* + *bb2*	15	6	3	1	2
*MD_2VB1_sc1*	14	5	4	1	2
*MD_2VB1_sc1* + *sc2*	5	0	0	0	0
*MD_2VB1_bb1* + *bb2* + *sc1* + *sc2*	3	0	0	0	0

Stereo-specific assignments for restraining were based on the ^3^*J*_*Hα-Hβ*_-coupling values calculated from the four unrestrained MD simulations starting from the four X-ray structures in case 4 or 3 of the unrestrained MD simulations suggested the same stereo-specific assignment. Only Glu 7 could not be stereo-specifically assigned using this criterion. Its ^3^*J*_*Hα-Hβ*_-coupling values were not included in the calculation

### Other quantities and agreement with NOE atom–atom distance bounds and *S*^2^ order-parameter values

The application of backbone ^3^*J*_*HNHα*_-coupling time-averaging local-elevation restraining in MD simulation does not significantly influence the secondary structure of the protein, with the four main α-helices, the two 3_10_-helices and the triple-stranded anti-parallel β-sheet in the protein all being maintained (see Fig. S1 and S2 of Supporting Information). However, there are some subtle differences in loop regions, particularly around residues Gly 102–Gly 104 and around Gly 117–Thr 118. For example, there are increases in the populations of the hydrogen bonds 107 NH–104 O and 118 NH–115 O and a decrease in the population of the hydrogen bond 104 NH–101 O compared to the unrestrained simulations. These regions are known to be mobile in solution, Gly 102, Asn 103 and Thr 118 all having lower backbone NH order parameters (0.72, 0.52 and 0.72, respectively; Buck et al. 1995) and these regions were less well defined in the NMR structure of HEWL (Schwalbe et al. 2001) having few longer range NOE identified for them. They provide an example of the extra conformational insights that could be obtained with time-averaging local-elevation restraining to glycine ^3^*J*_*HNHα*_-couplings although further experimental data would be needed to confirm the details of the hydrogen bond population changes observed here. As expected, the time-averaging local-elevation restraining does slightly enhance the atomic mobility, as observed from the backbone atom-positional fluctuations, see Figure S3 in Supporting Information.

Table 13 summarises for 1630 NOE atom–atom distance bounds of HEWL derived from NMR experiments (Smith et al. 1993; Schwalbe et al. 2001) the agreement between experimental NOE atom–atom distance bounds and the corresponding distances calculated from the four X-ray structures, from the four unrestrained MD simulation trajectories and from the ^3^*J*-coupling time-averaging local-elevation restrained MD simulation trajectories using different sets of backbone and side-chain restraints. Only ^3^*J*-coupling restraining to all 213 backbone and side-chain restraints does improve the agreement with experiment for the 1630 NOE atom–atom distance bounds.

**Table 13:** Number of NOE distance bound violations in four X-ray crystal structures, in the four unrestrained MD simulations starting from these, and in the ^3^*J*-coupling time-averaging local-elevation restrained MD simulations starting from the *2VB1* X-ray crystal structure and using different sets of backbone and side-chain restraints. Number of NOE distance bounds: 1630, see Table S1 in Supporting Information

Structure or simulation	Size of NOE distance bound violation (in nm)					
	0.05–0.1	0.1–0.15	0.15–0.2	0.2–0.25	0.25–0.3	> 0.3
*X-ray_2VB1*	21	7	5	0	0	0
*X-ray_4LZT*	20	7	4	0	0	0
*X-ray_1IEE*	20	7	5	0	0	0
*X-ray_1AKI*	15	10	4	0	0	0
*MD_2VB1*	44	18	11	5	3	5
*MD_4LZT*	41	13	13	5	3	5
*MD_1IEE*	43	20	13	8	3	5
*MD_1AKI*	44	15	14	2	3	8
*MD_2VB1_bb1* + *bb2*	38	20	10	4	1	7
*MD_2VB1_sc1*	36	19	11	5	1	1
*MD_2VB1_sc1* + *sc2*	46	24	10	5	2	8
*MD_2VB1_bb1* + *bb2* + *sc1* + *sc2*	30	11	5	3	2	2

Table 14 summarises for 51 *S*^2^_*CH*_ and 28 *S*^2^_*NH*_ side-chain order parameters of HEWL derived from NMR experiments (Buck et al. 1995; Moorman et al. 2012) the agreement between experimental *S*^2^ order parameter values and those calculated from the four unrestrained MD simulation trajectories and from the ^3^*J*-coupling time-averaging local-elevation restrained MD simulation trajectories using different sets of backbone and side-chain restraints. ^3^*J*-coupling restraining does not significantly change the agreement with experiment for the 79 *S*^2^ order parameters. This is not surprising, because the 79 *S*^2^ order parameters reflect motions along degrees of freedom that are different from the ones for which ^3^*J*-couplings are available.

**Table 14:** Number of deviations, |*S*^2^(exp) − *S*^2^(MD)|, for the 51 *S*^2^_*CH*_-values, the 11 *S*^2^_*NH*_-values of Trp and Arg residues and the 17 *S*^2^_*NH2*_-values of Asn and Gln residues, respectively (Smith et al. 2020), in the four unrestrained MD simulations starting from four different X-ray crystal structures and in the ^3^*J*-coupling time-averaging local-elevation restrained MD simulations starting from the *2VB1* X-ray crystal structure and using different sets of backbone and side-chain restraints. Order parameter values are reported in Table S2 in Supporting Information

Simulation	Size of *S*^2^ deviation					
	0.05–0.1	0.1–0.2	0.2–0.3	0.3–0.4	0.4–0.5	> 0.5
*MD_2VB1*	7/2/6	12/3/5	9/0/2	6/1/1	5/0/0	1/0/0
*MD_4LZT*	6/5/5	11/3/6	8/1/2	7/0/0	1/0/0	1/0/0
*MD_1IEE*	9/3/6	11/1/5	9/2/1	5/1/1	1/0/0	3/0/0
*MD_1AKI*	7/4/5	6/4/6	16/1/1	8/0/1	0/0/0	2/0/0
*MD_2VB1_bb1* + *bb2*	10/4/3	14/4/6	8/0/2	6/1/0	1/0/0	2/0/0
*MD_2VB1_sc1*	11/4/8	7/4/4	12/1/1	2/0/0	3/0/0	3/0/0
*MD_2VB1_sc1* + *sc2*	7/3/7	6/1/3	9/5/2	6/0/0	6/0/0	3/0/0
*MD_2VB1_bb1* + *bb2* + *sc1* + *sc2*	8/4/3	8/5/4	7/0/4	4/1/0	5/0/0	1/0/0

Table 15 summarises for 121 backbone *S*^2^_*NH*_ order parameters of HEWL derived from NMR experiments (Buck et al. 1995) the agreement between experimental *S*^2^ order parameter values and those calculated from the four unrestrained MD simulation trajectories and from the ^3^*J*-coupling time-averaging local-elevation restrained MD simulation trajectories using different sets of backbone and side-chain restraints. ^3^*J*-coupling restraining does reduce the number of deviations larger than 0.4, but the number of deviations larger than 0.2 is not reduced.

**Table 15:** Number of deviations, |*S*^2^(exp) − *S*^2^(MD)|, for the 121 backbone *S*^2^_*NH*_-values, (Buck et al. 1995), in the four unrestrained MD simulations starting from four different X-ray crystal structures and in the ^3^*J*-coupling time-averaging local-elevation restrained MD simulations starting from the *2VB1* X-ray crystal structure and using different sets of backbone and side-chain restraints. Order parameter values are reported in Table S3 in Supporting Information

Simulation	Size of *S*^2^ deviation					
	0.05–0.1	0.1–0.2	0.2–0.3	0.3–0.4	0.4–0.5	> 0.5
*MD_2VB1*	27	27	13	6	2	0
*MD_4LZT*	23	29	14	4	1	1
*MD_1IEE*	26	32	11	7	1	0
*MD_1AKI*	32	32	11	4	1	0
*MD_2VB1_bb1* + *bb2*	26	34	16	8	0	0
*MD_2VB1_sc1*	27	34	15	6	0	0
*MD_2VB1_sc1* + *sc2*	26	28	23	8	0	0
*MD_2VB1_bb1* + *bb2* + *sc1* + *sc2*	20	36	14	7	0	0

### Importance of time-averaging

**Fig. 4:**
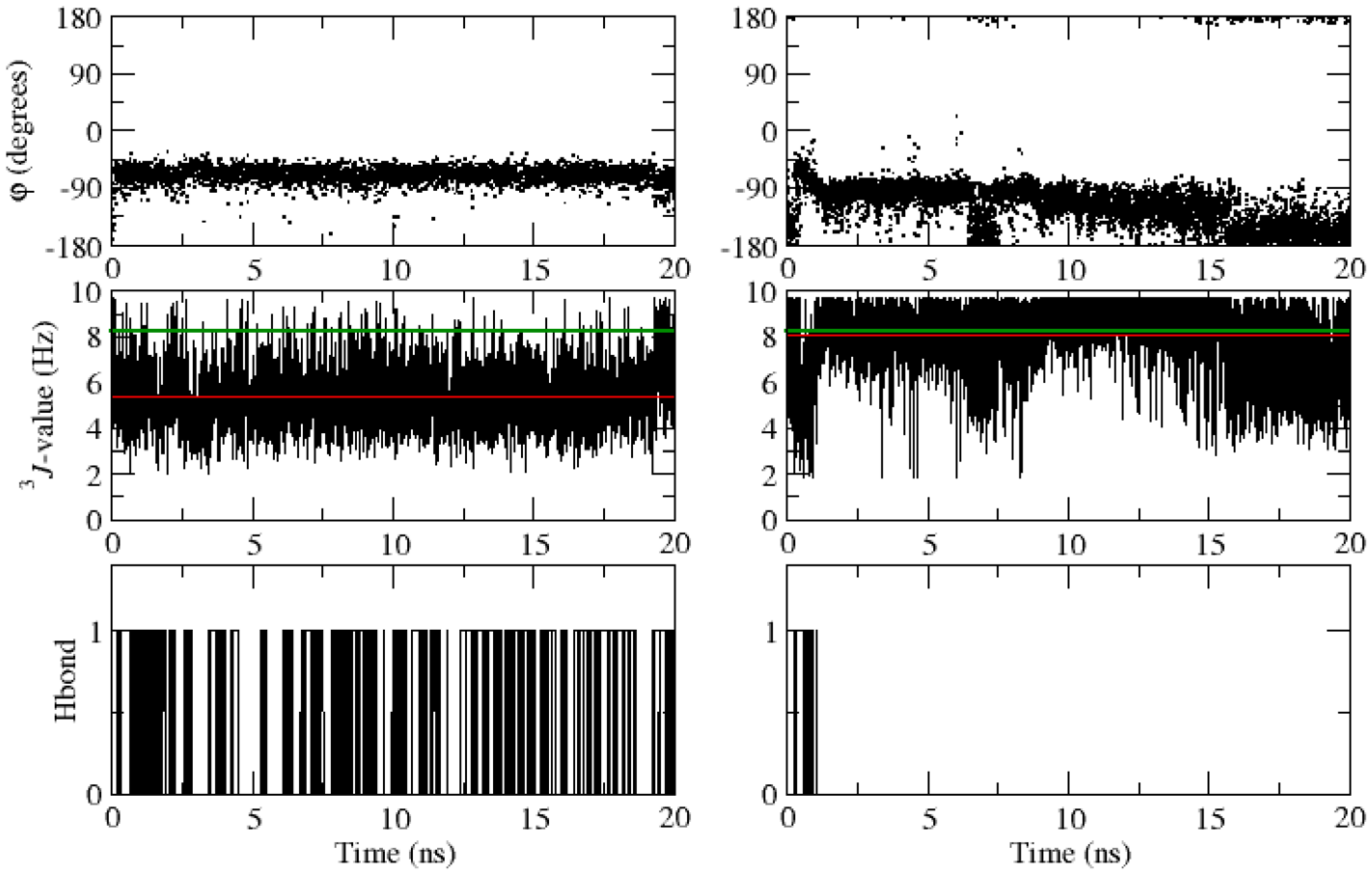
Variation of the backbone *φ*-angle (degree) determining the ^3^*J*_*HNHα*_-coupling, of this ^3^*J*_*HNHα*_-coupling (Hz) with an experimental value of 8.2 Hz (green line), and the presence of the Asn 103 NH–Asp 101 OD1 hydrogen bond for residue Asn 103 as function of time from the unrestrained MD simulation *MD_2VB1* (left panels) and from the ^3^*J*-coupling time-averaging local-elevation restraining (to all 213 experimental ^3^*J*-coupling values) MD simulation *MD_2VB1_bb1* + *bb2* + *sc1* + *sc2* (right panels), each starting from the *2VB1* X-ray structure. Red lines: average ^3^*J*-coupling value in the MD simulations

**Fig. 5:**
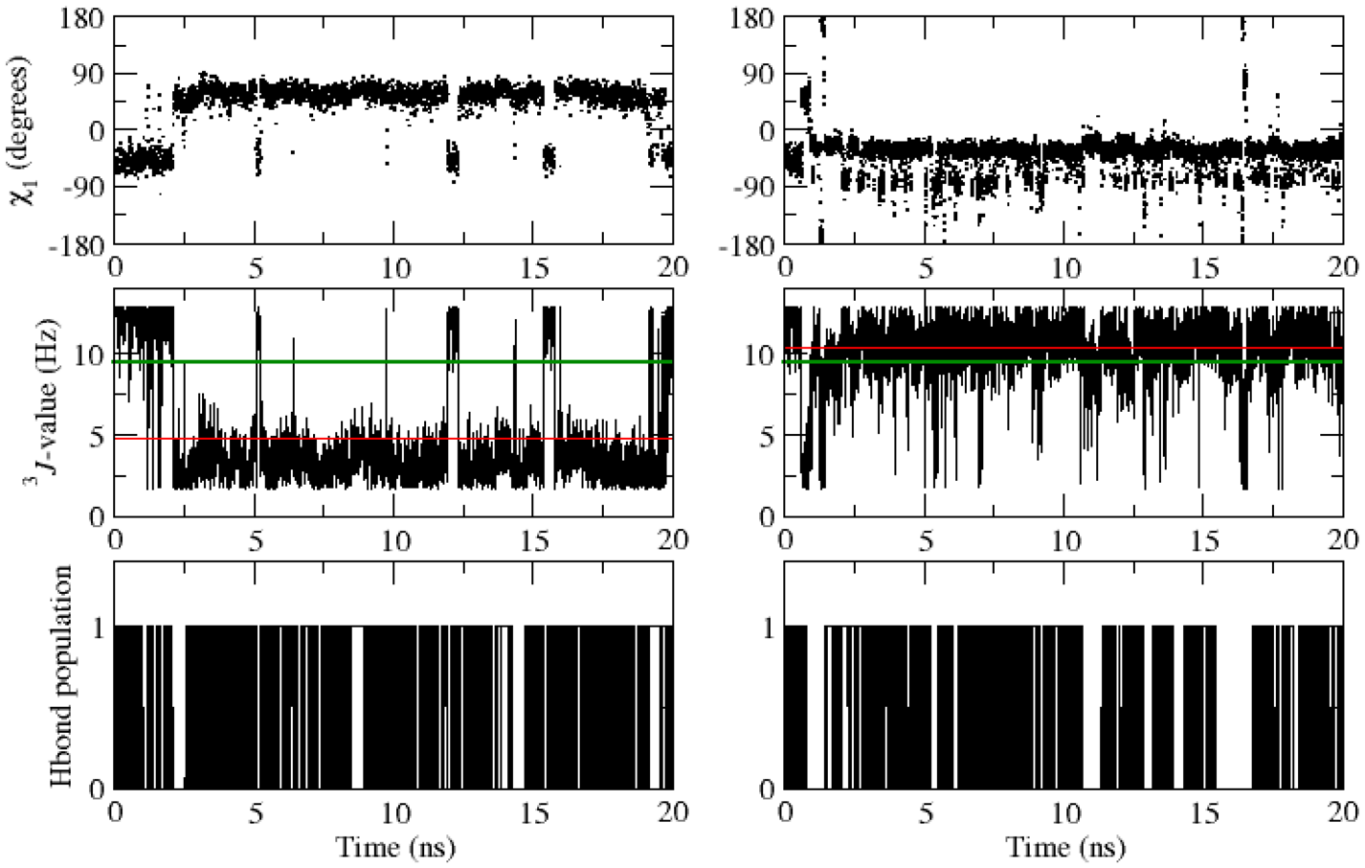
Variation of the side-chain *χ*_1_-angle (degree) determining the ^3^*J*_*Hα-Hβ*_-coupling, of this ^3^*J*_*Hα-Hβ*_-coupling (Hz) with an experimental value of 9.5 Hz (green line), and the presence of the Thr 89 OG1-HG1–Asp 87 OD1 hydrogen bond for residue Thr 89 as function of time from the unrestrained MD simulation *MD_2VB1* (left panels) and from the ^3^*J*-coupling time-averaging local-elevation restraining (to all 213 experimental ^3^*J*-coupling values) MD simulation *MD_VB1_bb1* + *bb2* + *sc1* + *sc2* (right panels), each starting from the *2VB1* X-ray structure. Red lines: average ^3^*J*-coupling value in the MD simulations

**Fig. 6:**
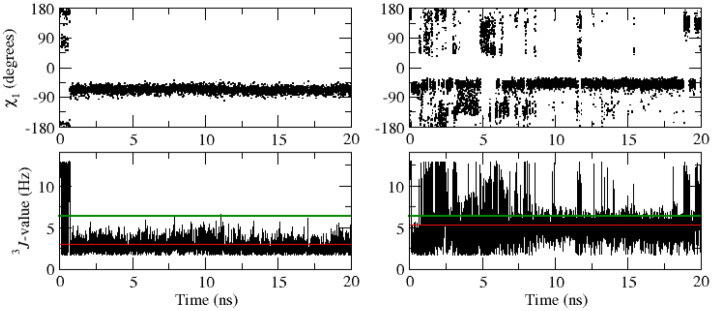
Variation of the side-chain *χ*_1_-angle (degree) determining the ^3^*J*_*Hα-Hβ*_-coupling, and of this ^3^*J*_*Hα-Hβ*_-coupling (Hz) with an experimental value of 6.3 Hz (green line), for residue Val 99 as function of time from the unrestrained MD simulation *MD_VB1* (left panels) and from the ^3^*J*-coupling time-averaging local-elevation restraining (to all 213 experimental ^3^*J*-coupling values) MD simulation *MD_VB1_bb1* + *bb2* + *sc1* + *sc2* (right panels), each starting from the *2VB1* X-ray structure. Red lines: average ^3^*J*-coupling value in the MD simulations

**Fig. 7:**
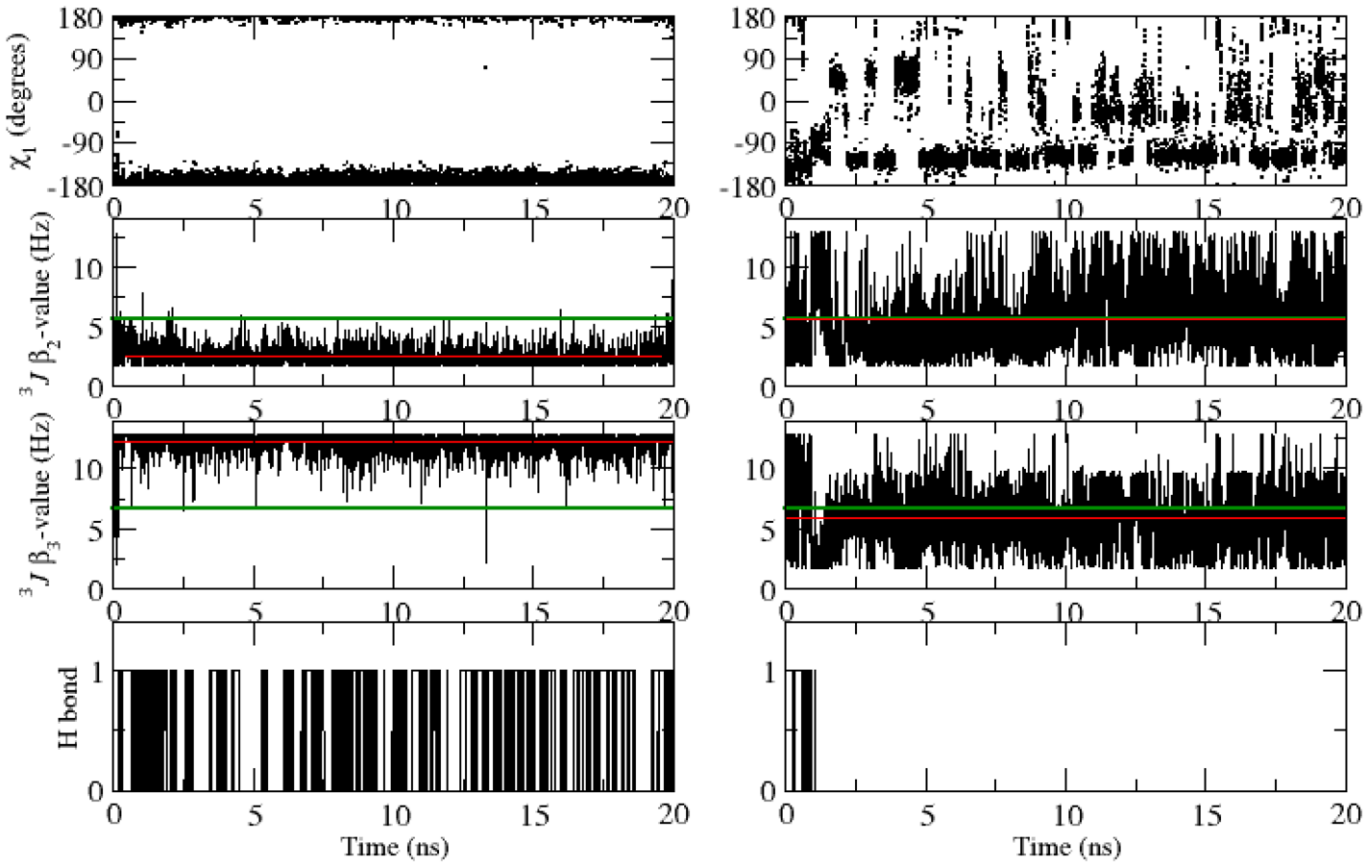
Variation of the side-chain *χ*_1_-angle (degree) determining the two, β_*2*_ and β_*3*_, ^3^*J*_*Hα-Hβ*_-couplings, of these two ^3^*J*_*Hα-Hβ*_-couplings (Hz) with experimental values of 5.6 Hz and 6.6 Hz respectively (green lines), and the presence of the Asn 103 NH–Asp 101 OD1 hydrogen bond for residue Asp 101 as function of time from the unrestrained MD simulation *MD_2VB1* (left panels) and from the ^3^*J*-coupling time-averaging local-elevation restraining (to all 213 experimental ^3^*J*-coupling values) MD simulation *MD_2VB1_bb1* + *bb2* + *sc1* + *sc2* (right panels), each starting from the *2VB1* X-ray structure. Red lines: average ^3^*J*-coupling value in the MD simulations

**Fig. 8:**
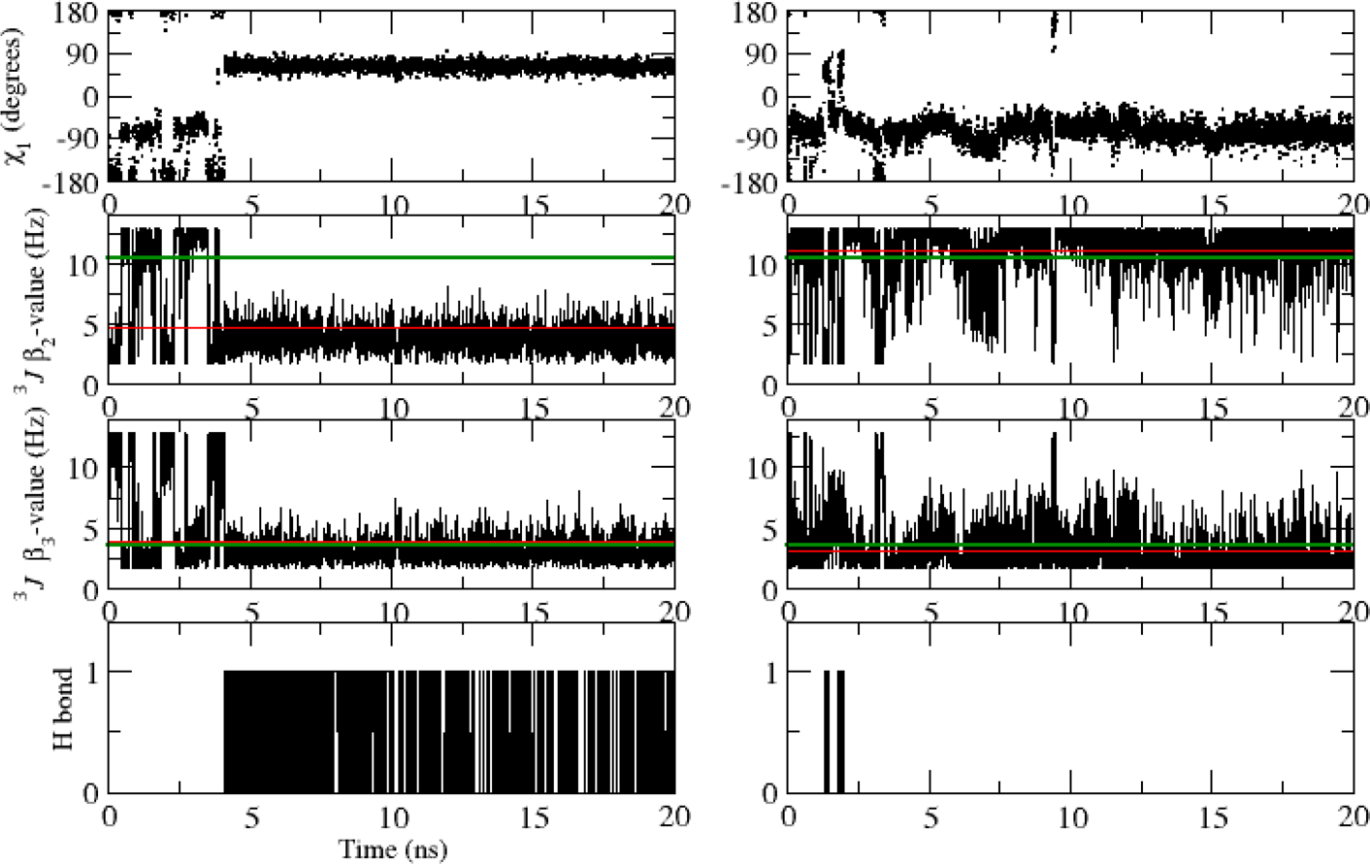
Variation of the side-chain *χ*_1_-angle (degree) determining the two, β_*2*_ and β_*3*_, ^3^*J*_*Hα-Hβ*_-couplings, of these two ^3^*J*_*Hα-Hβ*_-couplings (Hz) with experimental values of 10.5 Hz and 3.6 Hz respectively (green lines), and the presence of the Ala 107 NH–Asn 106 OD1 hydrogen bond for residue Asn 106 as function of time from the unrestrained MD simulation *MD_2VB1* (left panels) and from the ^3^*J*-coupling time-averaging local-elevation restraining (to all 213 experimental ^3^*J*-coupling values) MD simulation *MD_2VB1_bb1* + *bb2* + *sc1* + *sc2* (right panels), each starting from the *2VB1* X-ray structure. Red lines: average ^3^*J*-coupling value in the MD simulations

Values of measured ^3^*J*-couplings may the result from considerable conformational averaging. This is illustrated in Figs. 4, 5, 6, 7 and 8 for one backbone *φ*-angle and four side-chain *χ*_1_-angles.

Figure 4 shows for Asn 103 the variation of the backbone *φ*-angle determining the ^3^*J*_*HNHα*_-coupling, of this ^3^*J*_*HNHα*_-coupling with an experimental value of 8.2 Hz (green line), and the presence of the Asn 103 NH–Asp 101 OD1 hydrogen bond as function of time for the unrestrained (left panels) and the ^3^*J*-coupling time-averaging local-elevation restraining to all 213 experimental ^3^*J*-coupling values (right panels) MD simulations starting from the *2VB1* X-ray structure. A variation of the *φ*-angle of about 30° around its average value of − 69° leads to the ^3^*J*_*HNHα*_-coupling covering the range 2–10 Hz (RMSF 1.4 Hz) and an average value of 5.4 Hz (red line) in the unrestrained MD simulation, and in the ^3^*J*-coupling time-averaging local-elevation restraining simulation to a distribution of ^3^*J*_*HNHα*_-couplings shifted to larger values (RMSF 1.5 Hz) with an average of 8.1 Hz. The restraining shifts the average ^3^*J*_*HNHα*_-coupling more than 2 Hz towards the experimental value and slightly increases its fluctuation. The Asn 103 NH–Asp 101 OD1 hydrogen bond regularly populated in the unrestrained simulation disappears upon ^3^*J*_*HNHα*_-coupling restraining. The *2VB1* X-ray structure contains two *φ*-angle conformations, − 79° (main conformation) and − 93° (alternative conformation), with ^3^*J*_*HNHα*_-couplings of 6.6 Hz and 8.2 Hz respectively, of which only one matches the experimental ^3^*J*_*HNHα*_-coupling value. Both *φ*-angle values are covered in the MD simulations.

Figure 5 shows for Thr 89 the variation of the side-chain *χ*_1_-angle determining the ^3^*J*_*Hα-Hβ*_-coupling, of this ^3^*J*_*Hα-Hβ*_-coupling with an experimental value of 9.5 Hz (green line), and the presence of the Thr 89 OG1-HG1–Asp 87 OD1 hydrogen bond as function of time from the unrestrained (left panels) and ^3^*J*-coupling time-averaging local-elevation restraining to all 213 experimental ^3^*J*-coupling values (right panels) MD simulations starting from the *2VB1* X-ray structure. In the unrestrained simulation, the *χ*_1_-angle stays around + 60° with an occasional excursion to − 50°, leading to ^3^*J*_*Hα-Hβ*_-coupling values between 2 and 6 Hz, with an occasional excursion to 10–12 Hz. The average ^3^*J*_*Hα-Hβ*_-coupling of 4.8 Hz (RMSF 3.4 Hz) (red line) deviates substantially from the experimental value of 9.5 Hz. Restraining completely changes the *χ*_1_-angle and ^3^*J*_*Hα-Hβ*_-coupling distributions. The average *χ*_1_-angle becomes − 40° with large fluctuations (RMSF 31°) and the average ^3^*J*_*Hα-Hβ*_-coupling becomes 10.4 Hz (red line) with a much-reduced variation (RMSF 1.7 Hz). The presence of the Thr 89 OG1-HG1–Asp 87 OD1 hydrogen bond is somewhat reduced by the restraining. The *2VB1* X-ray *χ*_1_-angle values of − 67° or − 69° result in ^3^*J*_*Hα-Hβ*_-couplings of 12.8 Hz and 12.7 Hz respectively, deviations of more than 3 Hz from the experimental value of 9.5 Hz.

Figure 6 shows for Val 99 the variation of the side-chain *χ*_1_-angle determining the ^3^*J*_*Hα-Hβ*_-coupling and of this ^3^*J*_*Hα-Hβ*_-coupling with an experimental value of 6.3 Hz (green line), as function of time from the unrestrained (left panels) and ^3^*J*-coupling time-averaging local-elevation restraining to all 213 experimental ^3^*J*-coupling values (right panels) MD simulations starting from the *2VB1* X-ray structure. The unrestrained simulation shows a stable *χ*_1_-angle value of − 62° with little variation (RMSF 10°) resulting in an average ^3^*J*_*Hα-Hβ*_-coupling of 3.0 Hz (red line) (RMSF 1.6 Hz). It does not reproduce the experimental value of 6.3 Hz. The four X-ray structures deviate with ^3^*J*_*Hα-Hβ*_-coupling values of 12.8 Hz (*2VB1* and *4LZT*), 2.2 Hz (*1IEE*) and 2.4 Hz (*1AKI*) even more from the experimental value. Using ^3^*J*-coupling local-elevation restraining other conformations of the *χ*_1_-angle are accessed, resulting in a larger variation (RMSF 2.1 Hz) and raising the average to 5.3 Hz (red line). The *2VB1* X-ray structure contains two conformations, with *χ*_1_-angles of 176° (main conformation) and − 53° (alternative conformation) and ^3^*J*_*Hα-Hβ*_-couplings of 12.8 Hz and 4.2 Hz respectively. Averaging over different conformations seems to occur in aqueous solution.

Figure 7 shows for Asp 101 the variation of the side-chain *χ*_1_-angle determining the two, β_*2*_ and β_*3*_, ^3^*J*_*Hα-Hβ*_-couplings, of these two ^3^*J*_*Hα-Hβ*_-couplings (Hz) with experimental values of 5.6 Hz and 6.6 Hz respectively (green lines), and the presence of the Asn 103 NH–Asp 101 OD1 hydrogen bond as function of time from the unrestrained (left panels) and ^3^*J*-coupling local-elevation restraining to all 213 experimental ^3^*J*-coupling values (right panels) MD simulations starting from the *2VB1* X-ray structure. The unrestrained simulation shows a stable *χ*_1_-angle value of − 169° with little variation (RMSF 11°) resulting in average ^3^*J*_*Hα-Hβ*_-couplings of 2.5 Hz (RMSF 0.8 Hz) and 12.2 Hz (RMSF 0.9 Hz) (red lines). These values deviate significantly from the experimental values of 5.6 Hz and 6.6 Hz. The four X-ray structures deviate with *χ*_1_-angles of − 89° and ^3^*J*_*Hα-Hβ*_-coupling values of 10.5 Hz (β_*2*_) and 1.8 Hz (β_*3*_) (*2VB1* and *4LZT*), a *χ*_1_-angle of − 167° and ^3^*J*_*Hα-Hβ*_-couplings of 2.2 Hz (β_*2*_) and 12.4 Hz (β_*3*_) (*1IEE*) and a *χ*_1_-angle of − 139° and ^3^*J*_*Hα-Hβ*_-couplings of 2.4 Hz (β_*2*_) and 8.4 Hz (β_*3*_) (*1AKI*) even more from the experimental values. Using ^3^*J*-coupling time-averaging local-elevation restraining other conformations of the *χ*_1_-angle are accessed, resulting in a larger variations (RMSF 2.8 Hz (β_*2*_) and 2.2 Hz (β_*3*_)) of the ^3^*J*_*Hα-Hβ*_-couplings and raising the β_*2*_ average to 5.6 Hz and lowering the β_*3*_ average to 5.9 Hz (red line).

Figure 8 shows for Asn 106 the variation of the side-chain *χ*_1_-angle determining the two, β_*2*_ and β_*3*_, ^3^*J*_*Hα-Hβ*_-couplings, of these two ^3^*J*_*Hα-Hβ*_-couplings with experimental values of 10.5 Hz and 3.6 Hz respectively (green lines), and the presence of the Ala 107 NH–Asn 106 OD1 hydrogen bond as function of time from the unrestrained (left panels) and ^3^*J*-coupling local-elevation restraining to all 213 experimental ^3^*J*-coupling values (right panels) MD simulations starting from the *2VB1* X-ray structure. The unrestrained simulation shows after 4 ns a stable *χ*_1_-angle value of about 60° with little variation (RMSF 9°) resulting in average ^3^*J*_*Hα-Hβ*_-couplings of 4.7 Hz (RMSF 2.8 Hz) and 3.9 Hz (RMSF 2.7 Hz) (red lines). The β_*2*_ value deviates significantly from the experimental value of 10.5 Hz, while the β_*3*_ value is close to the experimental value of 3.6 Hz. The *2VB1* X-ray structure contains two *χ*_1_-angle conformations, − 96° (main conformation) and − 169° (alternative conformation), with ^3^*J*_*Hα-Hβ*_-coupling values of 9.3 Hz (β_*2*_) and 2.1 Hz (β_*3*_). The other three X-ray structures show only one conformation, with a *χ*_1_-angle of − 70° and ^3^*J*_*Hα-Hβ*_-couplings of 12.6 Hz (β_*2*_) and 2.3 Hz (β_*3*_) (*4LZT*), a *χ*_1_-angle of − 64° and ^3^*J*_*Hα-Hβ*_-couplings of 12.9 Hz (β_*2*_) and 3.0 Hz (β_*3*_) (*1IEE*) and a *χ*_1_-angle of − 70° and ^3^*J*_*Hα-Hβ*_-couplings of 12.6 Hz (β_*2*_) and 2.4 Hz (β_*3*_) (*1AKI*). Using ^3^*J*-coupling time-averaging local-elevation restraining other conformations of the *χ*_1_-angle are accessed, resulting in smaller variations (RMSF 2.3 Hz (β_*2*_) and 1.9 Hz (β_*3*_)) of the ^3^*J*_*Hα-Hβ*_-couplings, raising the β_*2*_ average to 11.1 Hz and lowering the β_*3*_ average to 3.1 Hz (red lines). The Ala 107 NH–Asn 106 OD1 hydrogen bond disappears when applying ^3^*J*-coupling restraining.

### Importance of escaping from torsional-angle energy minima

**Fig. 9:**
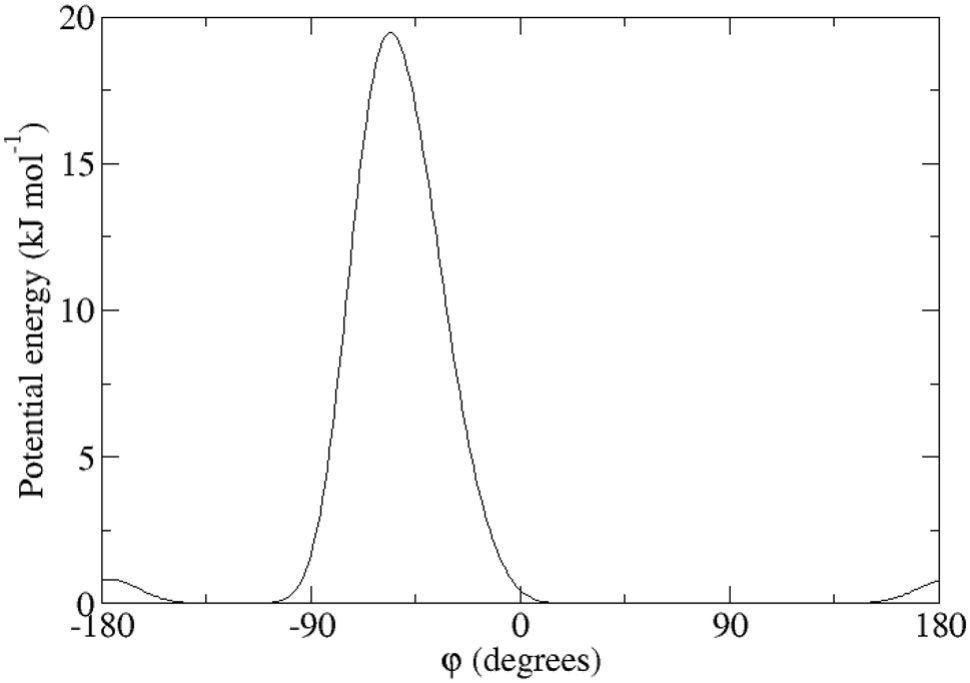
Local-elevation ^3^*J*_*HNHα*_-coupling restraining potential energy as function of the backbone *φ*-angle for residue Asn 103, built-up during the ^3^*J*-coupling time-averaging local-elevation restraining (to all 213 experimental ^3^*J*-coupling values) MD simulation *MD_2VB1_bb1* + *bb2* + *sc1* + *sc2* starting from the *2VB1* X-ray structure

**Fig. 10:**
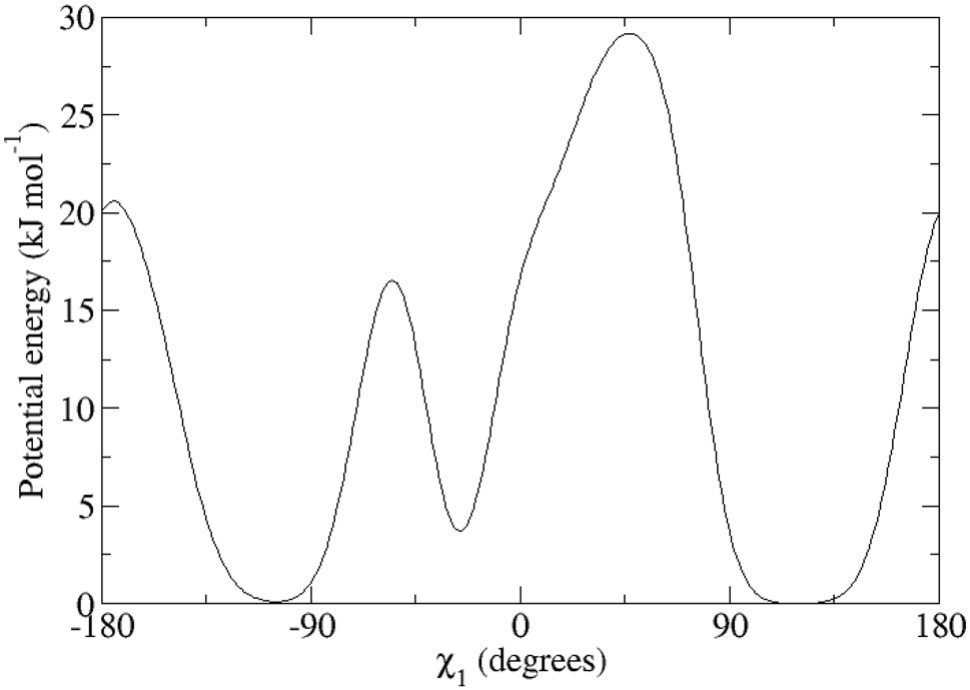
Local-elevation ^3^*J*_*HαHβ*_-coupling restraining potential energy as function of the side-chain *χ*_1_-angle for residue Thr 89, built-up during the ^3^*J*-coupling time-averaging local-elevation restraining (to all 213 experimental ^3^*J*-coupling values) MD simulation *MD_2VB1_bb1* + *bb2* + *sc1* + *sc2* starting from the *2VB1* X-ray structure

**Fig. 11:**
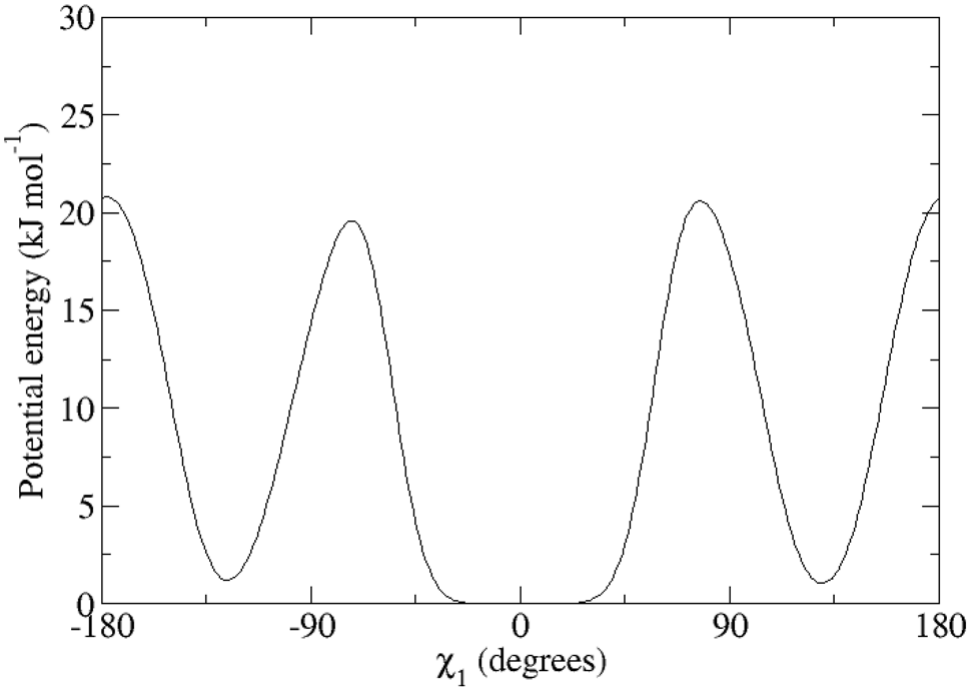
Local-elevation ^3^*J*_*HαHβ*_-coupling restraining potential energy as function of the side-chain *χ*_1_-angle for residue Val 99, built-up during the ^3^*J*-coupling time-averaging local-elevation restraining (to all 213 experimental ^3^*J*-coupling values) MD simulation *MD_2VB1_bb1* + *bb2* + *sc1* + *sc2* starting from the *2VB1* X-ray structure

**Fig. 12:**
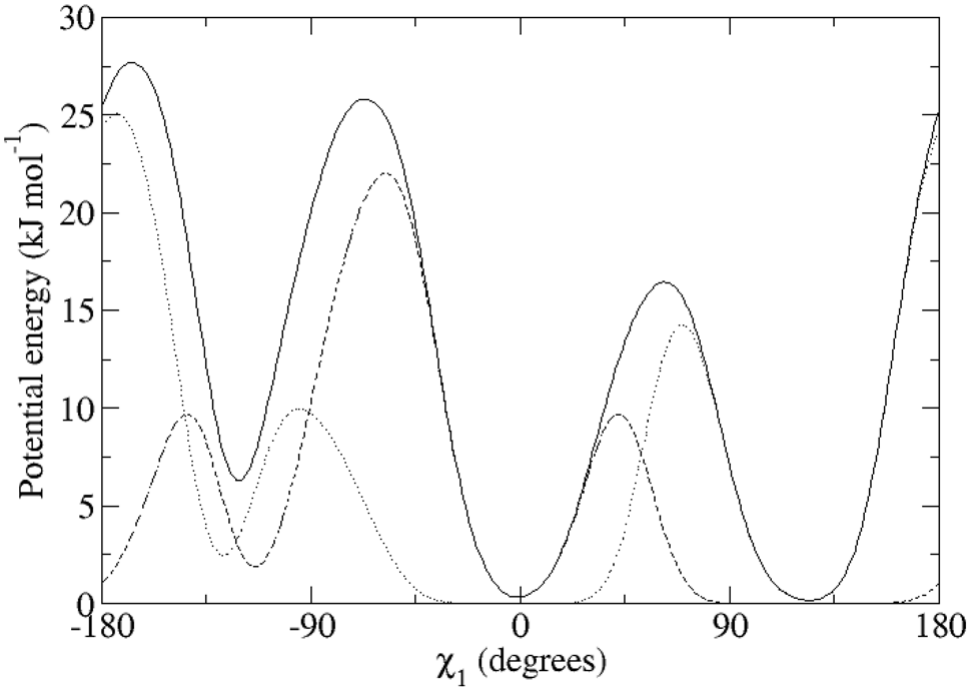
Local-elevation ^3^*J*_*HαHβ2*_-coupling (dashed line) and ^3^*J*_*HαHβ3*_-coupling (dotted line) restraining potential energies and their sum (solid line) as function of the side-chain *χ*_1_-angle for residue Asp 101, built-up during the ^3^*J*-coupling time-averaging local-elevation restraining (to all 213 experimental ^3^*J*-coupling values) MD simulation *MD_2VB1_bb1* + *bb2* + *sc1* + *sc2* starting from the *2VB1* X-ray structure

**Fig. 13:**
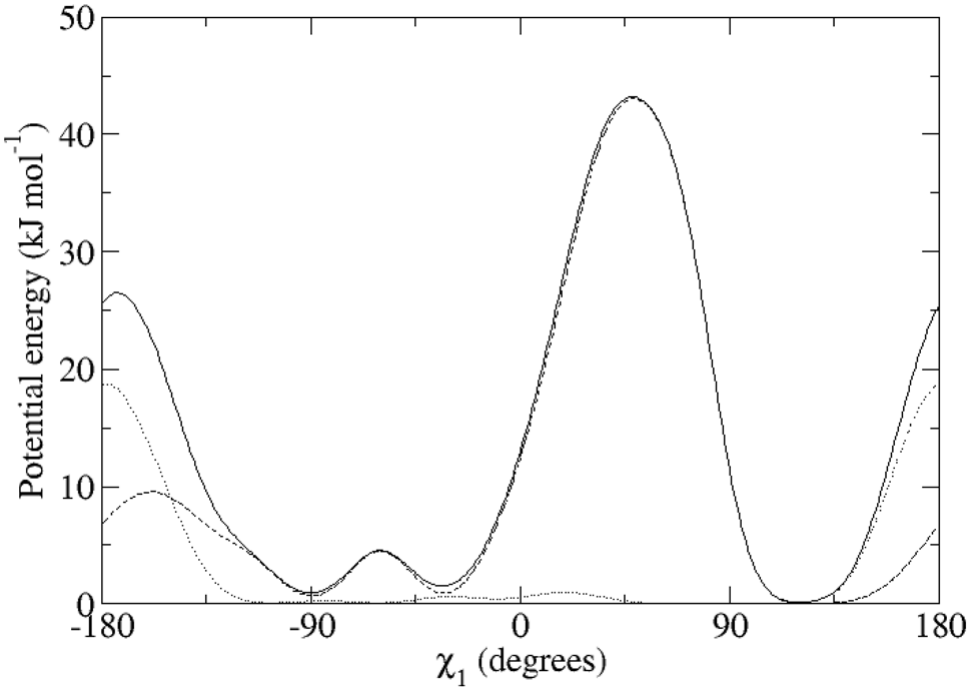
Local-elevation ^3^*J*_*HαHβ2*_-coupling (dashed line) and ^3^*J*_*HαHβ3*_-coupling (dotted line) restraining potential energies and their sum (solid line) as function of the side-chain *χ*_1_-angle for residue Asn 106, built-up during the ^3^*J*-coupling time-averaging local-elevation restraining (to all 213 experimental ^3^*J*-coupling values) MD simulation *MD_2VB1_bb1* + *bb2* + *sc1* + *sc2* starting from the *2VB1* X-ray structure

In Figs. 9, 10, 11, 12 and 13 the local-elevation potential energies after the time-averaging local-elevation restraining (to all 213 experimental ^3^*J*-coupling values) MD simulation *MD_2VB1_bb1* + *bb2* + *sc1* + *sc2* for the *φ*-angle of Asn 103 and the *χ*_1_-angles of Thr 89, Val 99, Asp 101 and Asn 106, are shown. The time series of these angles, the corresponding ^3^*J*-couplings and some hydrogen bonds in this simulation were shown in Figs. 4, 5, 6, 7 and 8 (right panels).

The backbone *φ*-angle of Asn 103 in the unrestrained simulation covers values in the range [− 90°, − 45°] (Fig. 3, upper left panel) yielding a ^3^*J*_*HNHα*_-coupling of 5.4 Hz, much lower than the experimental value of 8.2 Hz. In the Karplus curve for this ^3^*J*-coupling (black solid line in the left panel of Fig. 2) this value corresponds to *φ*-angle values of about − 93° and − 147°. Time-averaging local-elevation ^3^*J*-coupling restraining shifts the *φ*-angle values towards the range [− 180°, − 90°] (Fig. 4, upper right panel), shifting the ^3^*J*_*HNHα*_-coupling from 5.4 Hz in the unrestrained simulation to 8.1 Hz, close to the experimental value of 8.2 Hz. Figure 9 shows the local-elevation potential-energy term built-up in the ^3^*J*-coupling restraining simulation for *φ*-angle values in the range [− 90°, − 10°] that causes this shift. There is no build-up of local-elevation potential energy in the range [0°, + 160°], because these *φ*-angle values are not occurring in the ^3^*J*-coupling restraining simulation (Fig. 4, upper right panel).

The side-chain *χ*_1_-angle of Thr 89 in the unrestrained simulation covers values in two ranges, [+ 30°, + 70°] and, less frequently, [− 70°, − 30°] (Fig. 5, upper left panel) yielding a ^3^*J*_*Hα-Hβ*_-coupling of 4.8 Hz, much lower than the experimental value of 9.5 Hz. In the Karplus curve for this ^3^*J*-coupling (solid line in the right panel of Fig. 2) this value corresponds to *χ*_1_-angle values of about − 25° and − 95° or of about + 112° and + 128°, respectively. Time-averaging local-elevation ^3^*J*-coupling restraining shifts the *χ*_1_-angle values towards the range [− 10°, − 100°] (Fig. 5, upper right panel), shifting the ^3^*J*_*Hα-Hβ*_-coupling from 4.8 Hz in the unrestrained simulation to 10.4 Hz, near the experimental value of 9.5 Hz. Figure 10 shows the local-elevation potential-energy term built-up in the ^3^*J*-coupling restraining simulation for *χ*_1_-angle values in the range [− 10°, + 80°] and around − 55°, that causes this shift. The build-up of local-elevation potential energy around − 170° is caused by the *χ*_1_-angle occasionally visiting this region in the ^3^*J*-coupling restraining simulation (Fig. 5, upper right panel). We note that the solvent accessibility of the side chain of Thr 89 in the *2VB1* X-ray structure is 76%.

The side-chain *χ*_1_-angle of Val 99 in the unrestrained simulation initially covers values around 70° and 170° (Fig. 6, upper left panel) yielding ^3^*J*_*Hα-Hβ*_-couplings between 2 and 13 Hz. After about 1 ns, the *χ*_1_-angle stabilizes around − 62° with values in the range [− 50°, − 85°] yielding a ^3^*J*_*Hα-Hβ*_-coupling of 3.0 Hz, much lower than the experimental value of 6.3 Hz. In the Karplus curve for this ^3^*J*-coupling (dashed line in the right panel of Fig. 2) this value corresponds to four *χ*_1_-angle values of about − 129°, − 37°, + 37° and + 129°. The Karplus curve indicates that a slight shift of the *χ*_1_-angle distribution towards less negative values, for example − 40°, would yield a ^3^*J*_*Hα-Hβ*_-coupling of about 6 Hz. Time-averaging local-elevation ^3^*J*-coupling restraining indeed induces this slight shift towards *χ*_1_-angle values in the range [− 40°, − 65°] (Fig. 6, upper right panel), but also makes the *χ*_1_-angle repeatedly move over all angle values but those between − 40° and + 40°, thereby reaching very large ^3^*J*_*Hα-Hβ*_-coupling values, in order to raise the average ^3^*J*_*Hα-Hβ*_-coupling value from 3.0 Hz in the unrestrained simulation towards the experimental value of 6.3 Hz. Figure 11 shows the local-elevation potential-energy term built-up in the ^3^*J*-coupling restraining simulation for *χ*_1_-angle values around − 80°, + 80° and 180°. It shows minima for − 126°, + 130° and for [− 35°, + 35°]. The *χ*_1_-angle range [− 35°, + 35°] is disfavoured by the *χ*_1_ dihedral-angle potential-energy term and the non-bonded van der Waals interaction of the force field. We note that the solvent accessibility of the side chain of Val 99 in the *2VB1* X-ray structure is only 7%. This side chain is surrounded by the side chains of Tyr 20, Trp 28, Ile 98 and Tyr 108.

The last two examples involve longer side chains for which the C_β_-atom is connected to two hydrogens, H_β2_ and H_β3_. If their ^3^*J*_*Hα-Hβ*_-couplings have been stereo-specifically assigned, the corresponding *χ*_1_-angle can be restrained using two local-elevation ^3^*J*_*Hα-Hβ*_-coupling restraining potential-energy terms. This imposes more restriction on the *χ*_1_-angle motion than in the previously discussed cases.

The side-chain *χ*_1_-angle of Asp 101 covers in the unrestrained simulation values around − 170° (Fig. 7, upper left panel) yielding average ^3^*J*_*Hα-Hβ*_-couplings of 2.5 Hz for β_2_ and 12.2 Hz for β_3_, to be compared to experimental values of 5.6 Hz and 6.6 Hz respectively. In the Karplus curve for the ^3^*J*_*Hα-Hβ2*_-coupling (solid line in the right panel of Fig. 2) the experimental value of 5.6 Hz corresponds to four *χ*_1_-angle values of about − 116°, − 4°, + 76° and + 164°. In the Karplus curve for the ^3^*J*_*Hα-Hβ3*_-coupling (dashed line in the right panel of Fig. 2) the experimental value of 6.6 Hz corresponds to four *χ*_1_-angle values of about − 129°, − 37°, + 37° and + 129°. The Karplus curves suggest a *χ*_1_-angle distribution dominantly around − 125°. *χ*_1_-angle values around + 55° would bring both ^3^*J*_*Hα-Hβ*_-couplings near their experimental values, but would require averaging of ^3^*J*_*Hα-Hβ*_-couplings. Time-averaging local-elevation ^3^*J*-coupling restraining indeed shifts the *χ*_1_-angle distribution towards values around − 125° (Fig. 7, upper right panel), but also makes the *χ*_1_-angle repeatedly move over all angle values. Figure 12 shows the β_2_ and β_3_ local-elevation potential-energy terms (dashed and dotted lines, respectively) and their sum (solid line) built-up in the ^3^*J*-coupling restraining simulation. Major build-up is observed for *χ*_1_-angle values around − 165°, − 70° and + 65°. The first two keep the *χ*_1_-angle around − 125°. Yet, excursions to other *χ*_1_-angle values seem required to push the average ^3^*J*_*Hα-Hβ2*_- and ^3^*J*_*Hα-Hβ3*_-couplings towards the corresponding experimental values of 5.6 Hz and 6.6 Hz, respectively. We note that the solvent accessibility of the side chain of Asp 101 in the *2VB1* X-ray structure is 42%.

The side-chain *χ*_1_-angle of Asn 106 in the unrestrained simulation initially covers values around − 70° and − 170° (Fig. 8, upper left panel) yielding ^3^*J*_*Hα-Hβ2*_- and ^3^*J*_*Hα-Hβ3*_-couplings both fluctuating between 2 and 13 Hz. After about 4 ns, the *χ*_1_-angle stabilizes around + 60° yielding an average ^3^*J*_*Hα-Hβ2*_-coupling of 4.7 Hz and an average ^3^*J*_*Hα-Hβ3*_-coupling of 3.9 Hz, the former much lower than the experimental value of 10.5 Hz, the latter close to the experimental value of 3.6 Hz. In the Karplus curve for the ^3^*J*_*Hα-Hβ2*_-coupling (solid line in the right panel of Fig. 2) the experimental value of 10.5 Hz corresponds to two *χ*_1_-angle values of about − 89° and − 31°. In the Karplus curve for the ^3^*J*_*Hα-Hβ3*_-coupling (dashed line in the right panel of Fig. 2) the experimental value of 3.6 Hz corresponds to four *χ*_1_-angle values of about − 111°, − 58°, + 58° and + 111°. The Karplus curves suggest a *χ*_1_-angle distribution dominantly around − 75° with much averaging in the range [− 100°, − 45°]. Time-averaging local-elevation ^3^*J*-coupling restraining indeed shifts the *χ*_1_-angle distribution towards values around between − 100° and − 45° (Fig. 8, upper right panel). Figure 13 shows the β_2_ and β_3_ local-elevation potential-energy terms (dashed and dotted lines, respectively) and their sum (solid line) built-up in the ^3^*J*-coupling restraining simulation. Major build-up is observed for *χ*_1_-angle values between 0° and 90° and around − 170°. The small build-up at − 65° is due to the β_2_ restraint, keeping the ^3^*J*_*Hα-Hβ2*_-coupling below 12 Hz. The averaging between the two local-elevation energy minima in the range [− 100°, − 25°] results in average ^3^*J*_*Hα-Hβ2*_- and ^3^*J*_*Hα-Hβ3*_-couplings of 11.1 Hz and 3.1 Hz, close to the experimental values of 10.5 Hz and 3.6 Hz, respectively. We note that the solvent accessibility of the side chain of Asn 106 in the *2VB1* X-ray structure is 76%.

### Use of measured ^3^*J*-coupling values in force-field validation

Figures 4, 5, 6, 7 and 8 show examples of dihedral angles for which the ^3^*J*-couplings in the unrestrained simulations do not agree with the experimental values. The local-elevation potential-energy term used in the restraining simulations changes the dihedral-angle distribution such that the average ^3^*J*-coupling matches experiment. Thus such a potential-energy term contains information on a possible modification of the dihedral-angle term of the force field used. If a consistent picture of possible modifications would emerge from simulations of a collection of proteins, such a modification could be incorporated into the force field.

## Conclusions

Although ^3^*J*-coupling constants are relatively easy to obtain from NMR experiments, their use in structure determination of proteins has been rather limited due to different aspects of the measurement and the relation between a ^3^*J*-coupling and molecular structure (van Gunsteren et al. 2016). The Karplus relation between structure and ^3^*J*-coupling value is multi-valued, with up to four torsional angle values mapping to a single ^3^*J*-coupling value. In addition, intermediate ^3^*J*-coupling values (4–8 Hz) are sensitive to the experimental averaging period, which is rather long. This is in contrast to some other NMR measurable quantities such as NOE intensities. The difficulty of accounting for conformational averaging and for the multi-valued function of a torsional angle in terms of a ^3^*J*-coupling has severely hampered the use of ^3^*J*-couplings in protein structure determination or refinement. However, with the advent of time-averaging local-elevation restraining MD simulation (Christen et al. 2007; Smith et al. 2016) both problems could be solved. Time-averaging can be taken into account in a simulation (Torda et al. 1989) and a molecular conformation can be induced to escape from an (incorrect) local minima due to restraining based on the Karplus relation by use of the local-elevation algorithm (Huber et al. 1994). This makes a comprehensive use of ^3^*J*-coupling data in structure determination possible (Smith et al. 2016).

The application of ^3^*J*-coupling time-averaging local-elevation restraining to the protein HEWL shows that this technique is able to produce a conformational ensemble compatible with the experimental ^3^*J*-coupling data. Analysis of the conformations underlying the ^3^*J*-couplings shows that conformational averaging plays an essential role in a number of cases and that finding an alternative minimum energy conformation for backbone *φ* or side-chain *χ*_1_ angles is also of importance.

The ^3^*J*-coupling time-averaging local-elevation restraining does improve the agreement with 1630 NOE atom–atom distance bounds for HEWL, but only if all 213 backbone and side-chain ^3^*J*-coupling restraints are applied. It has no significant effect upon the agreement of the conformational ensemble with values of 121 backbone *S*^2^_*NH*_ and 79 side-chain *S*^2^_*CH*_ and *S*^2^_*NH*_ order parameters for HEWL. This one would more or less expect, considering the different degrees of freedom involved.

The results for the backbone ^3^*J*_*HNHα*_-couplings based on the parametrisation of the Karplus relation due to Pardi et al. (1984) show that this parametrisation is not capable of reproducing large ^3^*J*_*HNHα*_-coupling values, due to a maximum of 9.7 Hz of this Karplus relation. One could use alternative parametrisations, such as the one of Wang and Bax (1996) with a maximum of 10 Hz or the one of Brüschweiler and Case (1994) with a maximum of 11 Hz. However, in the present case of HEWL, no significant change of the agreement with experiment is observed by using the latter Karplus relation in the analysis.

Of particular interest from the work reported here, is the description of the behaviour of the side-chains with a high level of conformational mobility. Examples such as the data for the side-chains of Asp 101 and Asn 106 shown in Figs. 7 and 8 indicate that any hydrogen bonds involving flexible side-chains, which are present in crystal structures of the protein, may be very fluctuating or completely absent in solution. Indeed, the simulation results show just how conformationally disordered the surface of the protein really is. This needs to be kept in mind when X-ray structures are being used in areas such as drug design or to help with the interpretation of data from receptor binding or mutational studies.

## Supplementary Information

Below is the link to the electronic supplementary material.

Electronic supplementary material 1 (PDF 12901635 kb)
