# Supplementary material for: On the use of 3J-coupling NMR data to derive structural information on proteins
Source: J Biomol NMR. 2021 Jan 25;75(1):39–70. doi: 10.1007/s10858-020-00355-5 (PMC7897194; doi:10.1007/s10858-020-00355-5)

Supporting Information

Tables S1 – List of 1630 NOE atom-atom distance upper bounds and r^{-3} averaged distances calculated from the *2VB1* X-ray structure, the one unrestrained MD simulation and the four ^{3}J-coupling restraining MD simulations starting from this X-ray structure.

Hen lysozyme NOE data set (data set from Schwalbe et al. *Protein Science* **10,** 677-688 (2001)).

Note that NOE number 297 has been corrected and is now 28 HZ3 – 56 HD rather than 28 HZ3 – 56 HG.

The experimental upper bound for the interproton distance restraint is given together with the calculated distance in the *2VB1* X-ray structure and from the unrestrained *MD_2VB1* and restrained *MD_2VB1_bb1+bb2*, *MD_2VB1_sc1, MD_2VB1_sc1+sc2*, *MD_2VB1_bb1+bb2+sc1+sc2* simulations.

						Distance $<r^{-3}>^{-1/3}$ (nm)					
NOE number	Residue and atom 1		Residue and atom 2		Upper experimental bound (nm)	X-ray *2VB1*	*MD_2VB1*	*MD_2VB1 _bb1+bb2*	*MD_2VB1 _sc1*	*MD_2VB1 _sc1+sc2*	*MD_2VB1_ bb1+bb2+sc1+sc2*
1	1	HA	2	HN	0.25	0.22	0.23	0.23	0.23	0.23	0.23
2	2	HN	2	HA	0.45	0.29	0.28	0.28	0.28	0.28	0.28
3	2	HA	2	HB	0.30	0.29	0.27	0.26	0.28	0.28	0.28
4	2	HA	2	HG2@	0.55	0.29	0.30	0.32	0.30	0.30	0.30
5	2	HA	2	HG1@	0.55	0.28	0.30	0.29	0.29	0.30	0.29
6	2	HA	3	HN	0.25	0.22	0.22	0.22	0.22	0.22	0.22
7	2	HA	40	HN	0.45	0.34	0.37	0.36	0.37	0.37	0.36
8	2	HA	39	HA	0.25	0.24	0.25	0.27	0.25	0.26	0.26
9	2	HG2@	39	HA	0.60	0.37	0.42	0.45	0.41	0.41	0.40
10	2	HG1@	39	HA	0.75	0.42	0.45	0.42	0.45	0.46	0.44
11	2	HN	2	HB	0.30	0.26	0.25	0.28	0.25	0.25	0.25
12	2	HN	2	HG2@	0.60	0.34	0.32	0.31	0.31	0.31	0.32
13	2	HN	2	HG1@	0.75	0.45	0.37	0.38	0.42	0.42	0.42

14	2	HG2@	3	HN	0.75	0.51	0.44	0.47	0.49	0.48	0.47
15	2	HG1@	3	HN	0.60	0.38	0.38	0.39	0.37	0.36	0.35
16	2	HG2@	38	HN	0.75	0.78	0.72	0.77	0.76	0.75	0.74
17	2	HG1@	38	HN	0.60	0.61	0.65	0.65	0.63	0.63	0.62
18	2	HG2@	40	HN	0.75	0.50	0.56	0.59	0.55	0.55	0.53
19	2	HB	2	HG2@	0.60	0.23	0.24	0.24	0.24	0.24	0.24
20	2	HB	2	HG1@	0.60	0.24	0.24	0.24	0.24	0.24	0.24
21	2	HN	40	HN	0.55	0.49	0.51	0.52	0.51	0.51	0.51
22	2	HG1@	40	HN	0.85	0.59	0.62	0.58	0.62	0.63	0.62
23	3	HN	3	HA	0.45	0.28	0.28	0.28	0.28	0.28	0.28
24	3	HA	3	HB2	0.45	0.29	0.29	0.29	0.29	0.29	0.29
25	3	HA	3	HB1	0.30	0.26	0.26	0.26	0.26	0.26	0.26
26	3	HN	3	HB2	0.30	0.22	0.22	0.23	0.22	0.22	0.23
27	3	HN	3	HB1	0.45	0.35	0.34	0.34	0.34	0.34	0.34
28	3	HD@	3	HE@	1.13	0.28	0.28	0.28	0.28	0.28	0.28
29	3	HB2	3	HD@	0.89	0.20	0.21	0.21	0.21	0.21	0.21
30	3	HB1	3	HD@	0.74	0.20	0.21	0.21	0.21	0.21	0.21
31	3	HD@	8	HN	0.89	0.44	0.54	0.49	0.51	0.49	0.50
32	3	HN	3	HD@	0.89	0.33	0.33	0.34	0.33	0.33	0.34
33	3	HD@	4	HN	0.89	0.41	0.39	0.42	0.40	0.40	0.40
34	3	HD@	40	HN	0.89	0.60	0.60	0.57	0.57	0.59	0.59
35	3	HN	39	HA	0.45	0.36	0.36	0.37	0.35	0.35	0.36
36	3	HB1	8	HN	0.45	0.32	0.40	0.35	0.37	0.36	0.36
37	3	HD@	7	HB@	0.94	0.46	0.57	0.44	0.49	0.49	0.48
38	3	HD@	8	HD@	1.42	0.44	0.46	0.49	0.46	0.47	0.49
39	3	HZ	8	HD@	0.98	0.61	0.64	0.69	0.63	0.63	0.67
40	3	HB2	8	HD@	0.98	0.40	0.42	0.42	0.41	0.42	0.43
41	3	HD@	88	HG2@	1.19	0.53	0.63	0.54	0.58	0.60	0.53
42	3	HE@	88	HG2@	1.04	0.35	0.46	0.44	0.44	0.45	0.43
43	3	HZ	88	HG2@	0.55	0.33	0.43	0.44	0.41	0.41	0.41

44	3	HE@	55	HB	0.74	0.68	0.65	0.77	0.75	0.70	0.69
45	3	HD@	55	HB	0.74	0.65	0.60	0.69	0.68	0.66	0.65
46	3	HE@	39	HA	1.19	0.79	0.73	0.75	0.72	0.73	0.75
47	3	HN	40	HN	0.55	0.42	0.46	0.45	0.44	0.44	0.45
48	3	HB2	38	HB@	0.50	0.30	0.33	0.41	0.35	0.33	0.35
49	3	HB2	88	HD@	0.85	0.78	0.72	0.71	0.74	0.71	0.63
50	3	HN	4	HA@	0.75	0.59	0.57	0.56	0.56	0.57	0.57
51	3	HE@	40	HN	0.99	0.68	0.66	0.65	0.63	0.64	0.66
52	3	HE@	88	HD@	1.29	0.53	0.49	0.54	0.55	0.50	0.52
53	3	HD@	88	HD@	1.29	0.68	0.62	0.64	0.67	0.63	0.57
54	4	HN	4	HA@	0.65	0.24	0.24	0.24	0.24	0.24	0.24
55	4	HA@	5	HN	0.65	0.26	0.25	0.24	0.24	0.24	0.24
56	4	HN	7	HB@	0.50	0.33	0.35	0.31	0.32	0.33	0.33
57	5	HN	5	HA	0.30	0.28	0.27	0.26	0.27	0.27	0.27
58	6	HA	6	HB2	0.45	0.28	0.29	0.29	0.29	0.28	0.29
59	6	HA	6	HB1	0.30	0.24	0.24	0.24	0.24	0.24	0.24
60	6	HA	9	HN	0.45	0.32	0.36	0.35	0.35	0.36	0.34
61	6	HA	10	HN	0.45	0.41	0.39	0.39	0.38	0.39	0.38
62	6	HN	6	HB2	0.30	0.24	0.24	0.23	0.23	0.23	0.23
63	6	HN	6	HB1	0.45	0.36	0.34	0.34	0.34	0.34	0.34
64	6	HB1	127	HA	0.45	0.33	0.48	0.47	0.48	0.47	0.44
65	6	HA	9	HB@	0.55	0.32	0.35	0.34	0.35	0.36	0.33
66	6	HN	6	HA	0.45	0.28	0.27	0.27	0.27	0.27	0.27
67	6	HB1	7	HN	0.45	0.36	0.34	0.35	0.34	0.33	0.34
68	6	HN	7	HN	0.30	0.26	0.26	0.26	0.27	0.27	0.27
69	7	HN	7	HA	0.30	0.28	0.26	0.26	0.26	0.26	0.26
70	7	HN	7	HB@	0.50	0.29	0.25	0.26	0.26	0.26	0.26
71	7	HA	7	HB@	0.50	0.26	0.25	0.26	0.25	0.25	0.25
72	7	HA	11	HN	0.45	0.42	0.39	0.39	0.40	0.40	0.41
73	7	HA	10	HN	0.45	0.35	0.37	0.35	0.37	0.38	0.36

74	7	HA	10	HB@	0.75	0.39	0.37	0.35	0.37	0.38	0.36
75	7	HB@	8	HN	0.65	0.30	0.30	0.30	0.29	0.30	0.29
76	7	HN	8	HN	0.30	0.27	0.30	0.30	0.30	0.30	0.30
77	8	HN	8	HA	0.30	0.28	0.27	0.27	0.27	0.27	0.27
78	8	HN	8	HB1	0.30	0.25	0.26	0.28	0.27	0.26	0.27
79	8	HN	8	HB2	0.30	0.26	0.23	0.23	0.23	0.23	0.23
80	8	HA	8	HB1	0.45	0.29	0.29	0.27	0.28	0.29	0.28
81	8	HA	8	HB2	0.45	0.24	0.25	0.26	0.25	0.25	0.25
82	8	HA	8	HD@	0.78	0.35	0.35	0.35	0.34	0.34	0.33
83	8	HN	8	HD@	0.98	0.49	0.46	0.44	0.45	0.47	0.45
84	8	HA	11	HN	0.45	0.34	0.36	0.36	0.35	0.36	0.35
85	8	HA	9	HN	0.45	0.35	0.35	0.35	0.35	0.35	0.35
86	8	HN	9	HN	0.30	0.27	0.29	0.29	0.29	0.29	0.29
87	8	HN	10	HN	0.45	0.43	0.45	0.44	0.44	0.45	0.44
88	8	HA	11	HB@	0.60	0.36	0.35	0.36	0.35	0.36	0.35
89	8	HB@	9	HN	0.65	0.32	0.31	0.29	0.30	0.31	0.30
90	8	HD@	12	HN	0.98	0.53	0.59	0.61	0.60	0.58	0.58
91	8	HD@	9	HN	0.98	0.52	0.51	0.52	0.52	0.51	0.51
92	8	HD@	12	HE@	0.93	0.40	0.46	0.45	0.44	0.44	0.44
93	8	HD@	88	HD@	1.28	0.50	0.51	0.49	0.50	0.48	0.48
94	8	HD@	88	HG2@	1.28	0.52	0.57	0.53	0.51	0.56	0.53
95	8	HG	8	HD@	0.83	0.18	0.18	0.18	0.18	0.18	0.18
96	8	HD@	38	HE@	1.22	0.60	0.60	0.68	0.61	0.59	0.58
97	9	HN	9	HA	0.30	0.27	0.27	0.27	0.27	0.27	0.27
98	9	HN	9	HB@	0.55	0.27	0.27	0.27	0.27	0.27	0.27
99	9	HA	9	HB@	0.55	0.24	0.24	0.24	0.24	0.24	0.24
100	9	HA	12	HN	0.45	0.37	0.37	0.35	0.35	0.36	0.36
101	9	HA	10	HN	0.45	0.35	0.35	0.35	0.35	0.35	0.35
102	9	HN	10	HN	0.30	0.28	0.30	0.30	0.30	0.29	0.30
103	9	HN	11	HN	0.45	0.43	0.45	0.45	0.44	0.45	0.45

104	9	HN	129	HD@	0.98	0.64	1.13	1.17	1.05	0.93	0.91
105	9	HB@	124	HG2@	0.85	0.36	0.37	0.35	0.40	0.37	0.37
106	10	HN	10	HA	0.30	0.28	0.27	0.27	0.27	0.27	0.27
107	10	HN	10	HB@	0.55	0.28	0.27	0.27	0.27	0.27	0.27
108	10	HA	10	HB@	0.55	0.24	0.24	0.24	0.24	0.24	0.24
109	10	HA	12	HN	0.45	0.45	0.46	0.46	0.45	0.46	0.46
110	10	HA	11	HN	0.45	0.35	0.35	0.35	0.35	0.35	0.35
111	10	HN	11	HN	0.30	0.27	0.29	0.30	0.29	0.29	0.30
112	10	HN	12	HN	0.45	0.43	0.44	0.44	0.44	0.44	0.45
113	10	HN	129	HD@	0.83	0.50	1.08	1.14	1.06	0.83	0.71
114	10	HA	129	HD@	0.78	0.42	1.10	1.16	1.09	0.77	0.65
115	11	HN	11	HA	0.30	0.28	0.27	0.27	0.27	0.27	0.27
116	11	HN	11	HB@	0.55	0.27	0.27	0.27	0.27	0.27	0.27
117	11	HA	11	HB@	0.55	0.24	0.24	0.24	0.24	0.24	0.24
118	11	HA	12	HN	0.30	0.35	0.35	0.35	0.35	0.35	0.35
119	11	HN	12	HN	0.30	0.27	0.29	0.29	0.29	0.29	0.29
120	11	HN	13	HN	0.45	0.41	0.44	0.45	0.44	0.45	0.44
121	11	HB@	12	HN	0.55	0.33	0.32	0.33	0.33	0.33	0.32
122	11	HB@	13	HN	0.75	0.54	0.56	0.57	0.56	0.57	0.57
123	11	HA	14	HN	0.45	0.34	0.36	0.35	0.35	0.36	0.36
124	11	HB@	88	HD@	0.85	0.36	0.40	0.37	0.41	0.41	0.39
125	11	HN	88	HD@	0.85	0.55	0.62	0.55	0.60	0.60	0.55
126	12	HN	12	HA	0.30	0.28	0.27	0.27	0.27	0.27	0.27
127	12	HA	13	HN	0.45	0.35	0.35	0.35	0.35	0.35	0.35
128	12	HN	13	HN	0.30	0.27	0.29	0.29	0.29	0.29	0.30
129	12	HA	15	HB2	0.45	0.30	0.31	0.32	0.31	0.33	0.34
130	12	HN	88	HD@	0.75	0.38	0.50	0.39	0.44	0.44	0.45
131	12	HA	88	HD@	0.60	0.32	0.47	0.38	0.39	0.41	0.41
132	12	HE@	88	HD@	0.90	0.51	0.58	0.44	0.48	0.48	0.49
133	12	HE@	17	HD@	0.98	0.54	0.54	0.56	0.58	0.56	0.55

134	12	HA	17	HD@	0.98	0.51	0.56	0.55	0.51	0.62	0.53
135	12	HA	17	HG	0.45	0.41	0.55	0.55	0.50	0.56	0.50
136	12	HN	17	HD@	1.08	0.74	0.75	0.75	0.74	0.83	0.76
137	12	HN	14	HN	0.55	0.43	0.44	0.44	0.44	0.44	0.45
138	13	HN	13	HA	0.30	0.28	0.27	0.27	0.27	0.27	0.27
139	13	HN	13	HB@	0.50	0.24	0.24	0.25	0.25	0.24	0.24
140	13	HA	13	HB@	0.50	0.25	0.26	0.26	0.26	0.25	0.25
141	13	HN	14	HN	0.30	0.27	0.29	0.30	0.29	0.29	0.29
142	13	HA	14	HN	0.45	0.35	0.35	0.35	0.35	0.35	0.35
143	13	HN	129	HD@	0.98	0.60	1.46	1.56	1.40	1.11	0.98
144	13	HB@	14	HN	0.50	0.32	0.29	0.29	0.30	0.31	0.31
145	13	HN	25	HD@	0.83	0.38	0.40	0.42	0.42	0.39	0.39
146	13	HN	88	HD@	0.85	0.60	0.76	0.66	0.70	0.71	0.71
147	14	HN	14	HA	0.45	0.27	0.27	0.27	0.27	0.27	0.27
148	14	HN	14	HB@	0.50	0.30	0.28	0.27	0.28	0.28	0.28
149	14	HN	15	HN	0.30	0.27	0.29	0.29	0.29	0.29	0.29
150	15	HN	15	HA	0.45	0.28	0.28	0.28	0.27	0.28	0.28
151	15	HN	15	HB2	0.30	0.25	0.23	0.23	0.23	0.24	0.24
152	15	HN	15	HB1	0.45	0.36	0.35	0.35	0.34	0.35	0.36
153	15	HA	15	HB2	0.30	0.29	0.29	0.29	0.29	0.29	0.29
154	15	HN	16	HN	0.45	0.27	0.29	0.28	0.28	0.25	0.26
155	15	HB2	92	HG1@	0.60	0.34	0.39	0.37	0.44	0.39	0.34
156	15	HB2	92	HG2@	0.75	0.35	0.38	0.36	0.46	0.39	0.42
157	15	HE1	88	HG1@	0.65	0.41	0.70	0.66	0.69	0.71	0.73
158	15	HE1	88	HD@	0.60	0.55	0.72	0.77	0.73	0.81	0.81
159	15	HE1	92	HG2@	0.75	0.66	0.75	0.76	0.87	0.80	0.73
160	15	HE1	92	HG1@	0.75	0.77	0.78	0.80	0.88	0.80	0.73
161	16	HN	16	HA@	0.65	0.24	0.24	0.23	0.23	0.25	0.24
162	16	HN	17	HD@	1.28	0.67	0.71	0.73	0.67	0.67	0.68
163	16	HN	17	HN	0.45	0.26	0.28	0.29	0.30	0.37	0.34

164	17	HN	17	HA	0.30	0.28	0.28	0.28	0.28	0.21	0.22
165	17	HN	17	HB1	0.45	0.36	0.28	0.28	0.30	0.35	0.36
166	17	HN	17	HB2	0.45	0.24	0.25	0.25	0.25	0.31	0.26
167	17	HA	17	HB1	0.45	0.26	0.28	0.28	0.27	0.26	0.26
168	17	HA	17	HB2	0.45	0.29	0.26	0.25	0.26	0.28	0.28
169	17	HA	17	HD@	0.98	0.35	0.34	0.34	0.35	0.34	0.34
170	17	HN	17	HG	0.25	0.28	0.36	0.39	0.33	0.32	0.32
171	17	HG	17	HD@	0.83	0.18	0.18	0.18	0.18	0.18	0.18
172	17	HD@	28	HZ2	0.98	0.50	0.47	0.48	0.51	0.41	0.39
173	17	HD@	28	HE3	0.98	0.44	0.66	0.56	0.62	0.55	0.57
174	17	HD@	28	HH2	0.98	0.52	0.64	0.59	0.62	0.53	0.47
175	17	HD@	18	HN	0.98	0.59	0.51	0.50	0.51	0.55	0.57
176	17	HD@	28	HE1	0.98	0.50	0.37	0.40	0.46	0.39	0.42
177	17	HA	18	HN	0.30	0.33	0.24	0.23	0.26	0.30	0.31
178	17	HN	18	HN	0.30	0.26	0.30	0.32	0.26	0.30	0.30
179	17	HG	18	HN	0.45	0.49	0.40	0.37	0.42	0.45	0.47
180	17	HD@	28	HZ3	0.98	0.50	0.72	0.63	0.68	0.59	0.56
181	17	HD@	92	HG1@	1.13	0.37	0.46	0.43	0.41	0.46	0.44
182	17	HD@	92	HG2@	1.13	0.46	0.52	0.50	0.45	0.49	0.51
183	17	HB@	28	HE3	0.65	0.55	0.86	0.76	0.82	0.69	0.66
184	17	HD@	28	HD1	0.98	0.52	0.41	0.44	0.50	0.46	0.51
185	17	HB@	17	HD@	1.18	0.26	0.26	0.26	0.26	0.26	0.26
186	17	HB@	17	HG	0.45	0.25	0.25	0.25	0.25	0.25	0.25
187	17	HD@	19	HN	1.28	0.75	0.79	0.78	0.80	0.79	0.80
188	17	HD@	20	HD@	1.42	0.57	0.74	0.67	0.74	0.65	0.65
189	17	HD@	20	HB2	0.83	0.51	0.70	0.63	0.65	0.60	0.61
190	17	HB@	28	HZ3	0.75	0.67	0.95	0.86	0.90	0.79	0.71
191	17	HD@	92	HB	1.08	0.55	0.54	0.60	0.44	0.58	0.56
192	17	HD@	88	HD@	1.58	0.69	0.88	0.77	0.68	0.83	0.78
193	17	HD@	96	HN	1.08	0.53	0.48	0.48	0.57	0.47	0.45

194	17	HA	28	HE1	0.55	0.42	0.56	0.57	0.59	0.48	0.45
195	17	HD@	20	HE@	1.72	0.73	0.76	0.67	0.89	0.78	0.76
196	17	HD@	55	HG2@	1.58	0.89	1.15	1.06	1.12	1.08	1.02
197	18	HN	18	HA	0.45	0.28	0.27	0.27	0.28	0.27	0.27
198	18	HN	18	HB2	0.30	0.26	0.25	0.23	0.25	0.24	0.25
199	18	HN	18	HB1	0.30	0.26	0.27	0.27	0.25	0.25	0.25
200	18	HA	18	HB2	0.30	0.24	0.26	0.26	0.25	0.24	0.24
201	18	HA	18	HB1	0.45	0.29	0.26	0.27	0.28	0.28	0.29
202	18	HA	19	HN	0.25	0.22	0.24	0.25	0.24	0.22	0.22
203	18	HN	25	HD@	0.98	0.48	0.60	0.61	0.50	0.47	0.51
204	18	HN	25	HN	0.55	0.55	0.63	0.67	0.61	0.56	0.56
205	18	HA	28	HE1	0.55	0.44	0.60	0.59	0.54	0.55	0.40
206	19	HN	19	HA	0.30	0.22	0.24	0.25	0.23	0.21	0.21
207	19	HN	19	HB@	0.65	0.34	0.28	0.27	0.27	0.31	0.32
208	19	HA	19	HB@	0.50	0.26	0.26	0.25	0.26	0.25	0.24
209	19	HN	20	HN	0.30	0.26	0.37	0.36	0.36	0.32	0.32
210	19	HN	24	HN	0.75	0.43	0.61	0.66	0.59	0.54	0.47
211	19	HN	28	HE1	0.55	0.38	0.70	0.64	0.62	0.61	0.51
212	20	HN	20	HB2	0.30	0.26	0.25	0.24	0.25	0.24	0.23
213	20	HN	20	HB1	0.30	0.26	0.30	0.32	0.26	0.26	0.25
214	20	HA	20	HB2	0.25	0.24	0.26	0.28	0.25	0.24	0.25
215	20	HA	20	HB1	0.45	0.29	0.26	0.25	0.29	0.29	0.29
216	20	HB2	20	HD@	0.74	0.21	0.21	0.21	0.21	0.21	0.21
217	20	HB1	20	HD@	0.74	0.21	0.21	0.21	0.21	0.21	0.21
218	20	HA	20	HD@	0.89	0.27	0.28	0.27	0.27	0.27	0.27
219	20	HN	20	HA	0.45	0.28	0.28	0.27	0.27	0.27	0.27
220	20	HD@	21	HN	0.89	0.35	0.38	0.41	0.35	0.36	0.36
221	20	HN	28	HE1	0.45	0.31	0.60	0.47	0.50	0.53	0.49
222	20	HA	21	HN	0.25	0.21	0.22	0.22	0.21	0.21	0.21
223	20	HE@	96	HB@	1.09	0.37	0.51	0.50	0.44	0.47	0.48

224	20	HN	21	HN	0.55	0.45	0.43	0.44	0.43	0.44	0.44
225	20	HE@	21	HN	0.99	0.47	0.56	0.61	0.49	0.50	0.51
226	21	HN	21	HA	0.25	0.22	0.22	0.22	0.21	0.21	0.21
227	21	HA	21	HB@	0.50	0.25	0.26	0.26	0.26	0.26	0.26
228	21	HA	22	HN	0.45	0.28	0.24	0.25	0.26	0.26	0.26
229	21	HN	22	HN	0.30	0.28	0.32	0.31	0.30	0.29	0.30
230	21	HN	22	HA@	0.75	0.51	0.54	0.53	0.52	0.52	0.52
231	21	HA	23	HN	0.75	0.46	0.42	0.43	0.44	0.44	0.44
232	21	HN	23	HN	0.75	0.44	0.45	0.45	0.45	0.46	0.45
233	22	HN	22	HA@	0.65	0.24	0.23	0.24	0.23	0.23	0.24
234	23	HN	23	HA	0.45	0.29	0.28	0.28	0.28	0.28	0.28
235	23	HN	23	HB2	0.45	0.25	0.25	0.25	0.24	0.24	0.23
236	23	HN	23	HB1	0.45	0.36	0.36	0.35	0.35	0.35	0.35
237	23	HA	23	HB1	0.30	0.25	0.25	0.25	0.25	0.25	0.25
238	23	HA	23	HD@	0.89	0.26	0.28	0.27	0.27	0.27	0.27
239	23	HE@	111	HH2	0.89	0.51	0.48	0.38	0.42	0.39	0.47
240	23	HB2	23	HD@	0.74	0.21	0.21	0.21	0.21	0.21	0.21
241	23	HB1	23	HD@	0.74	0.21	0.21	0.21	0.21	0.21	0.21
242	23	HB1	28	HE1	0.45	0.30	0.39	0.37	0.39	0.36	0.29
243	23	HD@	24	HN	0.89	0.46	0.43	0.44	0.42	0.43	0.45
244	23	HD@	111	HZ2	0.89	0.57	0.56	0.41	0.41	0.37	0.48
245	23	HD@	105	HB@	1.09	0.47	0.57	0.65	0.84	0.77	0.56
246	23	HE@	111	HZ2	0.74	0.53	0.39	0.37	0.40	0.48	0.46
247	23	HE@	105	HB@	1.09	0.41	0.43	0.53	0.73	0.61	0.44
248	23	HD@	28	HE1	0.89	0.44	0.57	0.54	0.57	0.52	0.47
249	23	HN	28	HZ2	0.30	0.47	0.72	0.58	0.58	0.64	0.53
250	23	HE@	111	HE1	0.99	0.67	0.45	0.51	0.36	0.61	0.52
251	23	HE@	99	HG@	1.27	0.53	0.56	0.65	0.60	0.60	0.54
252	23	HN	28	HE1	0.55	0.38	0.66	0.54	0.59	0.57	0.52
253	23	HN	24	HN	0.55	0.43	0.44	0.42	0.44	0.44	0.40

254	24	HN	24	HA	0.30	0.27	0.28	0.28	0.28	0.28	0.27
255	25	HN	25	HA	0.30	0.27	0.25	0.26	0.26	0.26	0.26
256	25	HN	25	HB@	0.50	0.23	0.27	0.26	0.25	0.24	0.27
257	25	HN	26	HN	0.45	0.29	0.30	0.30	0.30	0.29	0.31
258	25	HN	25	HG	0.30	0.45	0.26	0.26	0.26	0.31	0.24
259	25	HN	25	HD@	0.98	0.47	0.40	0.40	0.41	0.43	0.39
260	25	HA	25	HB@	0.65	0.26	0.26	0.26	0.26	0.26	0.26
261	25	HB@	25	HD@	1.03	0.26	0.26	0.26	0.26	0.26	0.26
262	25	HB@	26	HN	0.50	0.32	0.32	0.31	0.32	0.31	0.29
263	25	HA	26	HN	0.45	0.35	0.34	0.34	0.34	0.34	0.35
264	25	HD@	28	HD1	0.83	0.54	0.53	0.50	0.50	0.51	0.53
265	25	HN	28	HE1	0.55	0.56	0.49	0.53	0.50	0.56	0.51
266	25	HA	28	HE1	0.55	0.47	0.43	0.40	0.36	0.42	0.41
267	26	HN	26	HA@	0.65	0.24	0.23	0.23	0.23	0.23	0.23
268	26	HN	27	HN	0.30	0.28	0.30	0.29	0.29	0.29	0.30
269	26	HN	29	HG@	0.98	0.66	0.63	0.62	0.64	0.64	0.63
270	26	HN	120	HG@	0.98	0.60	0.51	0.44	0.51	0.50	0.45
271	26	HN	30	HN	0.75	0.59	0.60	0.60	0.61	0.61	0.60
272	26	HN	29	HN	0.55	0.49	0.50	0.50	0.51	0.50	0.50
273	26	HN	28	HN	0.55	0.43	0.45	0.46	0.47	0.46	0.47
274	27	HN	27	HA	0.45	0.27	0.27	0.27	0.27	0.27	0.27
275	27	HN	27	HB2	0.30	0.23	0.33	0.24	0.22	0.22	0.22
276	27	HN	27	HB1	0.45	0.35	0.26	0.34	0.34	0.34	0.34
277	27	HA	27	HB2	0.30	0.29	0.25	0.29	0.29	0.29	0.29
278	27	HA	27	HB1	0.30	0.26	0.23	0.25	0.25	0.26	0.26
279	27	HB2	28	HN	0.30	0.28	0.33	0.25	0.27	0.26	0.26
280	27	HA	30	HN	0.45	0.37	0.37	0.38	0.36	0.38	0.37
281	27	HN	28	HN	0.30	0.28	0.29	0.28	0.28	0.28	0.28
282	27	HA	30	HB2	0.45	0.38	0.37	0.38	0.38	0.43	0.39
283	27	HN	120	HG@	0.98	0.43	0.75	0.55	0.66	0.73	0.52

284	27	HA	111	HE1	0.30	0.31	0.52	0.38	0.55	0.50	0.37
285	27	HA	123	HE1	0.75	0.74	0.54	0.67	0.68	0.71	0.65
286	27	HB1	111	HE1	0.30	0.26	0.37	0.26	0.35	0.31	0.29
287	27	HN	28	HE1	0.75	0.61	0.62	0.58	0.57	0.60	0.57
288	28	HN	28	HA	0.30	0.28	0.27	0.27	0.27	0.27	0.27
289	28	HA	28	HB@	0.65	0.26	0.25	0.25	0.25	0.26	0.25
290	28	HB@	29	HN	0.65	0.31	0.30	0.31	0.30	0.30	0.30
291	28	HN	29	HN	0.30	0.27	0.29	0.28	0.28	0.28	0.28
292	28	HH2	28	HZ2	0.45	0.25	0.25	0.25	0.25	0.25	0.25
293	28	HE3	28	HZ3	0.25	0.25	0.25	0.25	0.25	0.25	0.25
294	28	HZ3	28	HH2	0.30	0.25	0.25	0.25	0.25	0.25	0.25
295	28	HE3	28	HA	0.30	0.27	0.25	0.27	0.27	0.27	0.28
296	28	HD1	28	HB@	0.65	0.31	0.29	0.30	0.30	0.30	0.30
297	28	HZ3	56	HD@	0.59	0.41	0.44	0.40	0.40	0.36	0.38
298	28	HE1	28	HZ2	0.30	0.29	0.28	0.28	0.28	0.28	0.28
299	28	HE1	28	HN	0.45	0.47	0.47	0.45	0.44	0.46	0.44
300	28	HE1	28	HD1	0.25	0.26	0.25	0.25	0.25	0.25	0.25
301	28	HE3	56	HD@	0.83	0.34	0.32	0.34	0.33	0.30	0.32
302	28	HE3	32	HB@	0.75	0.65	0.66	0.65	0.72	0.66	0.65
303	28	HH2	95	HB@	0.75	0.41	0.42	0.41	0.41	0.38	0.38
304	28	HE3	95	HB@	0.75	0.46	0.52	0.44	0.41	0.47	0.49
305	28	HZ3	95	HB@	0.60	0.32	0.47	0.39	0.35	0.38	0.36
306	28	HZ3	108	HE1	0.75	0.54	0.42	0.71	0.82	0.53	0.78
307	28	HZ3	98	HG2@	1.05	0.66	0.49	0.54	0.58	0.50	0.62
308	28	HH2	99	HG@	0.83	0.37	0.39	0.39	0.33	0.40	0.34
309	28	HH2	99	HB	0.30	0.38	0.53	0.41	0.35	0.39	0.42
310	28	HZ2	99	HG@	0.83	0.41	0.28	0.35	0.39	0.33	0.36
311	28	HZ3	99	HN	0.55	0.61	0.62	0.60	0.52	0.58	0.57
312	28	HE3	88	HD@	1.05	0.87	1.04	0.91	0.94	0.91	0.92
313	29	HN	29	HA	0.45	0.28	0.27	0.27	0.27	0.27	0.27

314	29	HA	29	HB	0.45	0.29	0.27	0.27	0.28	0.28	0.28
315	29	HN	29	HG1@	0.75	0.44	0.39	0.38	0.43	0.42	0.42
316	29	HN	29	HG2@	0.60	0.30	0.29	0.29	0.27	0.28	0.29
317	29	HA	30	HN	0.45	0.35	0.35	0.35	0.35	0.35	0.35
318	29	HN	30	HN	0.30	0.27	0.28	0.29	0.29	0.29	0.29
319	29	HB	30	HN	0.30	0.27	0.28	0.27	0.26	0.26	0.26
320	29	HG@	30	HN	0.83	0.42	0.41	0.40	0.42	0.41	0.41
321	30	HN	30	HA	0.30	0.28	0.27	0.27	0.27	0.27	0.27
322	30	HN	30	HB2	0.30	0.23	0.23	0.23	0.24	0.25	0.24
323	30	HN	30	HB1	0.30	0.24	0.23	0.24	0.21	0.21	0.22
324	30	HB2	31	HN	0.45	0.38	0.38	0.37	0.38	0.40	0.38
325	30	HB1	31	HN	0.30	0.26	0.25	0.25	0.26	0.28	0.26
326	30	HN	120	HG@	0.83	0.60	0.82	0.82	0.88	0.91	0.83
327	30	HB1	123	HE1	0.55	0.52	0.49	0.55	0.62	0.58	0.53
328	31	HN	31	HA	0.30	0.27	0.27	0.27	0.26	0.26	0.27
329	31	HA	31	HB@	0.55	0.24	0.24	0.24	0.24	0.24	0.24
330	31	HA	32	HN	0.45	0.35	0.35	0.35	0.35	0.35	0.35
331	31	HA	34	HN	0.45	0.37	0.36	0.36	0.37	0.35	0.36
332	31	HN	32	HN	0.45	0.28	0.29	0.30	0.30	0.30	0.30
333	32	HN	32	HA	0.30	0.28	0.27	0.27	0.27	0.27	0.27
334	32	HN	32	HB@	0.55	0.27	0.26	0.27	0.26	0.27	0.27
335	32	HA	32	HB@	0.55	0.24	0.24	0.24	0.24	0.24	0.24
336	32	HA	33	HN	0.45	0.35	0.35	0.35	0.34	0.35	0.35
337	32	HA	35	HB@	0.50	0.35	0.44	0.38	0.57	0.39	0.37
338	32	HN	33	HN	0.45	0.27	0.30	0.30	0.29	0.30	0.30
339	32	HB@	33	HN	0.60	0.34	0.33	0.32	0.36	0.33	0.33
340	32	HB@	38	HE@	1.04	0.54	0.44	0.60	0.47	0.50	0.47
341	32	HB@	35	HN	0.75	0.54	0.59	0.57	0.80	0.58	0.57
342	32	HB@	38	HD@	0.99	0.51	0.45	0.58	0.45	0.48	0.46
343	32	HN	56	HD@	0.83	0.48	0.54	0.49	0.53	0.52	0.54

344	33	HN	33	HA	0.30	0.27	0.27	0.27	0.27	0.27	0.27
345	33	HA	34	HN	0.45	0.35	0.35	0.35	0.35	0.35	0.35
346	33	HN	34	HN	0.30	0.28	0.30	0.30	0.29	0.30	0.30
347	33	HA	38	HE@	0.89	0.35	0.39	0.49	0.41	0.41	0.41
348	33	HN	38	HE@	0.89	0.42	0.39	0.49	0.43	0.41	0.40
349	33	HN	38	HD@	0.89	0.51	0.50	0.59	0.52	0.49	0.49
350	34	HN	34	HA	0.45	0.28	0.27	0.27	0.27	0.27	0.27
351	34	HN	34	HB2	0.30	0.24	0.23	0.23	0.25	0.24	0.24
352	34	HN	34	HB1	0.45	0.36	0.33	0.35	0.35	0.32	0.34
353	34	HA	34	HB2	0.25	0.29	0.28	0.29	0.29	0.28	0.29
354	34	HA	34	HB1	0.30	0.25	0.26	0.25	0.24	0.25	0.25
355	34	HN	35	HN	0.30	0.24	0.28	0.27	0.39	0.29	0.27
356	34	HD@	34	HE@	1.18	0.28	0.28	0.28	0.28	0.28	0.28
357	34	HB2	34	HD@	0.74	0.21	0.21	0.21	0.21	0.21	0.21
358	34	HB1	34	HD@	0.74	0.21	0.21	0.21	0.21	0.21	0.21
359	34	HA	34	HD@	0.74	0.26	0.27	0.27	0.28	0.28	0.27
360	34	HE@	114	HB@	1.09	0.43	0.45	0.41	0.40	0.41	0.40
361	34	HE@	123	HZ2	0.89	0.32	0.59	0.44	0.65	0.68	0.45
362	35	HN	35	HA	0.45	0.28	0.28	0.27	0.21	0.27	0.27
363	35	HN	35	HB@	0.45	0.29	0.29	0.28	0.32	0.28	0.28
364	35	HA	35	HB@	0.65	0.26	0.26	0.26	0.26	0.26	0.26
365	35	HN	36	HN	0.30	0.26	0.28	0.30	0.35	0.29	0.29
366	35	HB@	38	HE@	1.09	0.96	1.00	1.10	1.13	1.00	0.98
367	35	HN	38	HE@	0.89	0.78	0.81	0.92	0.96	0.82	0.82
368	36	HN	36	HA	0.45	0.28	0.27	0.28	0.27	0.28	0.28
369	36	HN	36	HB1	0.45	0.31	0.28	0.30	0.28	0.30	0.29
370	36	HN	36	HB2	0.45	0.38	0.34	0.28	0.32	0.32	0.35
371	36	HA	37	HN	0.45	0.32	0.32	0.24	0.34	0.30	0.32
372	36	HB1	39	HN	0.45	0.40	0.42	0.41	0.45	0.41	0.44
373	36	HB2	39	HN	0.45	0.27	0.29	0.46	0.35	0.31	0.30

374	36	HN	37	HN	0.30	0.21	0.25	0.35	0.26	0.25	0.24
375	36	HB1	55	HA	0.30	0.25	0.36	0.29	0.32	0.32	0.31
376	36	HB2	55	HA	0.30	0.27	0.35	0.26	0.29	0.30	0.31
377	36	HA	42	HB@	0.75	0.35	0.35	0.47	0.34	0.40	0.38
378	37	HN	37	HA	0.25	0.22	0.21	0.25	0.21	0.21	0.21
379	37	HA	37	HB@	0.50	0.26	0.25	0.25	0.26	0.26	0.25
380	37	HA	38	HN	0.45	0.27	0.26	0.23	0.24	0.25	0.26
381	37	HN	38	HN	0.45	0.27	0.27	0.35	0.29	0.29	0.28
382	37	HA	39	HN	0.45	0.44	0.45	0.44	0.44	0.45	0.45
383	38	HN	38	HA	0.25	0.21	0.20	0.20	0.20	0.20	0.20
384	38	HN	38	HB@	0.65	0.37	0.36	0.35	0.35	0.36	0.36
385	38	HA	38	HB@	0.45	0.26	0.26	0.26	0.26	0.26	0.26
386	38	HB@	38	HD@	1.09	0.19	0.19	0.19	0.19	0.19	0.19
387	39	HN	39	HA	0.30	0.29	0.28	0.28	0.28	0.28	0.28
388	39	HN	39	HB2	0.30	0.26	0.26	0.25	0.26	0.26	0.26
389	39	HN	39	HB1	0.30	0.24	0.25	0.25	0.24	0.25	0.24
390	39	HA	39	HB2	0.30	0.25	0.24	0.24	0.24	0.24	0.24
391	39	HA	39	HB1	0.45	0.29	0.29	0.29	0.29	0.29	0.29
392	39	HA	41	HN	0.45	0.36	0.40	0.39	0.39	0.39	0.39
393	39	HB1	42	HN	0.45	0.34	0.33	0.36	0.37	0.37	0.36
394	39	HA	40	HN	0.25	0.21	0.21	0.21	0.21	0.21	0.21
395	39	HA	42	HN	0.55	0.47	0.49	0.51	0.51	0.51	0.50
396	39	HN	40	HN	0.55	0.44	0.44	0.45	0.44	0.44	0.44
397	39	HA	40	HA	0.55	0.41	0.41	0.41	0.41	0.41	0.41
398	40	HN	40	HA	0.45	0.27	0.27	0.27	0.27	0.27	0.27
399	40	HA	40	HB	0.25	0.25	0.26	0.26	0.25	0.25	0.25
400	40	HA	40	HG2@	0.55	0.30	0.28	0.28	0.29	0.29	0.29
401	40	HN	41	HN	0.30	0.28	0.28	0.27	0.28	0.28	0.28
402	40	HA	55	HN	0.45	0.30	0.34	0.35	0.33	0.34	0.33
403	40	HA	42	HN	0.45	0.37	0.40	0.39	0.38	0.38	0.39

404	40	HN	40	HG2@	0.55	0.30	0.32	0.32	0.31	0.31	0.31
405	40	HB	40	HG2@	0.55	0.24	0.24	0.24	0.24	0.24	0.24
406	40	HN	42	HN	0.55	0.44	0.45	0.46	0.46	0.46	0.46
407	41	HN	41	HA	0.45	0.29	0.27	0.28	0.28	0.28	0.28
408	41	HN	42	HN	0.30	0.26	0.28	0.28	0.29	0.29	0.27
409	42	HN	42	HA	0.30	0.27	0.27	0.27	0.27	0.27	0.27
410	42	HN	42	HB@	0.55	0.27	0.27	0.27	0.27	0.27	0.27
411	42	HA	42	HB@	0.55	0.24	0.24	0.24	0.24	0.24	0.24
412	42	HA	43	HN	0.25	0.22	0.22	0.22	0.22	0.22	0.22
413	42	HB@	43	HN	0.55	0.37	0.37	0.36	0.38	0.38	0.38
414	42	HB@	43	HA	0.75	0.47	0.49	0.48	0.49	0.49	0.48
415	43	HN	43	HA	0.45	0.28	0.28	0.28	0.28	0.28	0.28
416	43	HA	43	HB	0.25	0.25	0.26	0.25	0.25	0.26	0.25
417	43	HA	44	HN	0.25	0.23	0.23	0.23	0.23	0.23	0.23
418	43	HA	54	HN	0.45	0.34	0.43	0.35	0.37	0.38	0.36
419	43	HA	53	HA	0.30	0.30	0.34	0.31	0.32	0.32	0.32
420	43	HN	43	HB	0.45	0.38	0.35	0.36	0.36	0.36	0.36
421	43	HN	43	HG2@	0.75	0.39	0.38	0.38	0.38	0.38	0.38
422	43	HB	43	HG2@	0.55	0.24	0.24	0.24	0.24	0.24	0.24
423	43	HA	51	HG2@	0.60	0.43	0.51	0.46	0.40	0.44	0.45
424	44	HN	44	HA	0.30	0.29	0.28	0.28	0.28	0.28	0.28
425	44	HN	44	HB@	0.65	0.29	0.25	0.26	0.25	0.26	0.26
426	44	HA	44	HB@	0.45	0.25	0.25	0.25	0.25	0.25	0.25
427	44	HN	52	HB2	0.45	0.30	0.29	0.31	0.30	0.30	0.29
428	44	HN	51	HG2@	0.60	0.34	0.50	0.42	0.39	0.42	0.40
429	44	HA	45	HN	0.25	0.22	0.23	0.22	0.22	0.22	0.22
430	44	HN	45	HN	0.55	0.41	0.45	0.44	0.43	0.43	0.44
431	45	HN	45	HA	0.45	0.29	0.28	0.28	0.28	0.28	0.28
432	45	HN	45	HB@	0.50	0.30	0.27	0.27	0.27	0.28	0.29
433	45	HA	45	HB@	0.65	0.25	0.26	0.26	0.26	0.24	0.24

434	45	HA	46	HN	0.30	0.22	0.21	0.22	0.22	0.22	0.22
435	45	HA	52	HN	0.45	0.37	0.35	0.36	0.36	0.35	0.35
436	45	HA	51	HA	0.25	0.26	0.28	0.27	0.27	0.27	0.27
437	45	HA	51	HG2@	0.60	0.36	0.42	0.40	0.38	0.39	0.39
438	46	HN	46	HA	0.45	0.28	0.28	0.28	0.28	0.28	0.28
439	46	HN	46	HB2	0.30	0.24	0.22	0.24	0.25	0.25	0.25
440	46	HN	46	HB1	0.45	0.35	0.28	0.32	0.35	0.35	0.35
441	46	HA	46	HB2	0.45	0.29	0.26	0.28	0.29	0.29	0.29
442	46	HA	46	HB1	0.45	0.25	0.28	0.26	0.24	0.24	0.25
443	46	HA	47	HN	0.25	0.23	0.25	0.24	0.24	0.24	0.24
444	46	HB2	47	HN	0.30	0.37	0.38	0.37	0.36	0.36	0.36
445	46	HB1	47	HN	0.30	0.27	0.29	0.27	0.26	0.25	0.25
446	46	HB2	48	HN	0.30	0.31	0.47	0.37	0.36	0.36	0.34
447	46	HB1	48	HN	0.30	0.26	0.32	0.30	0.31	0.32	0.30
448	46	HN	51	HA	0.45	0.26	0.32	0.33	0.32	0.33	0.34
449	46	HA	48	HN	0.55	0.40	0.46	0.44	0.45	0.45	0.44
450	46	HN	48	HN	0.75	0.50	0.53	0.51	0.53	0.53	0.51
451	46	HN	49	HN	0.55	0.47	0.43	0.43	0.43	0.44	0.44
452	47	HN	47	HA	0.30	0.28	0.27	0.26	0.26	0.26	0.26
453	47	HA	47	HB	0.25	0.25	0.26	0.26	0.26	0.26	0.26
454	47	HB	47	HG2@	0.55	0.24	0.24	0.24	0.24	0.24	0.24
455	47	HN	48	HN	0.30	0.26	0.28	0.29	0.29	0.29	0.29
456	47	HN	47	HG2@	0.60	0.31	0.32	0.32	0.32	0.32	0.32
457	47	HN	49	HN	0.55	0.43	0.42	0.44	0.44	0.45	0.44
458	48	HN	48	HA	0.45	0.28	0.27	0.27	0.27	0.27	0.27
459	48	HN	48	HB2	0.45	0.36	0.34	0.34	0.34	0.34	0.34
460	48	HN	48	HB1	0.45	0.26	0.25	0.25	0.25	0.25	0.25
461	48	HA	48	HB2	0.25	0.23	0.24	0.24	0.24	0.24	0.25
462	48	HA	48	HB1	0.25	0.24	0.24	0.24	0.24	0.24	0.24
463	49	HN	49	HA@	0.65	0.24	0.25	0.24	0.25	0.25	0.24

464	49	HN	50	HN	0.45	0.27	0.26	0.26	0.26	0.26	0.26
465	50	HN	50	HA	0.45	0.28	0.27	0.27	0.27	0.27	0.27
466	50	HN	50	HB@	0.65	0.30	0.29	0.30	0.30	0.27	0.31
467	50	HA	51	HN	0.45	0.25	0.24	0.24	0.23	0.23	0.24
468	51	HN	51	HA	0.45	0.29	0.28	0.28	0.28	0.28	0.28
469	51	HN	51	HB	0.45	0.28	0.31	0.28	0.28	0.28	0.28
470	51	HA	51	HB	0.30	0.29	0.27	0.28	0.28	0.28	0.28
471	51	HB	51	HG2@	0.55	0.24	0.24	0.24	0.24	0.24	0.24
472	51	HA	51	HG2@	0.55	0.31	0.28	0.29	0.28	0.28	0.28
473	51	HA	52	HN	0.25	0.22	0.22	0.22	0.22	0.22	0.23
474	51	HG2@	52	HN	0.55	0.31	0.40	0.36	0.36	0.36	0.34
475	51	HG2@	53	HE@	1.04	0.35	0.48	0.43	0.44	0.45	0.44
476	51	HG2@	53	HD@	1.19	0.43	0.54	0.49	0.48	0.50	0.48
477	51	HN	51	HG2@	0.75	0.46	0.38	0.43	0.44	0.43	0.44
478	51	HB	52	HN	0.45	0.40	0.34	0.37	0.39	0.39	0.38
479	51	HB	53	HE@	0.74	0.28	0.37	0.35	0.35	0.37	0.35
480	51	HN	53	HE@	0.99	0.51	0.60	0.59	0.59	0.61	0.59
481	51	HG2@	59	HA	0.85	0.64	0.72	0.67	0.67	0.68	0.67
482	52	HN	52	HA	0.45	0.29	0.28	0.28	0.28	0.28	0.28
483	52	HN	52	HB2	0.30	0.26	0.24	0.24	0.24	0.24	0.25
484	52	HN	52	HB1	0.45	0.36	0.35	0.35	0.35	0.35	0.35
485	52	HA	52	HB2	0.45	0.29	0.29	0.29	0.29	0.29	0.29
486	52	HA	53	HN	0.25	0.23	0.22	0.22	0.22	0.22	0.22
487	52	HA	59	HA	0.30	0.24	0.23	0.23	0.24	0.23	0.23
488	52	HA	53	HE@	0.89	0.59	0.65	0.62	0.67	0.67	0.65
489	52	HA	57	HN	0.55	0.63	0.66	0.68	0.66	0.65	0.66
490	52	HA	53	HD@	0.89	0.46	0.48	0.47	0.50	0.49	0.48
491	52	HN	53	HN	0.55	0.45	0.44	0.44	0.44	0.44	0.44
492	52	HB1	59	HA	0.55	0.41	0.40	0.41	0.42	0.41	0.40
493	53	HN	53	HB2	0.30	0.26	0.23	0.24	0.22	0.22	0.23

494	53	HN	53	HB1	0.45	0.37	0.35	0.35	0.34	0.34	0.34
495	53	HA	53	HB2	0.45	0.29	0.29	0.29	0.29	0.29	0.29
496	53	HA	53	HB1	0.30	0.26	0.26	0.25	0.26	0.26	0.26
497	53	HB2	53	HD@	0.74	0.20	0.21	0.21	0.21	0.21	0.21
498	53	HB1	53	HD@	0.74	0.20	0.21	0.21	0.21	0.21	0.21
499	53	HA	54	HN	0.30	0.22	0.22	0.22	0.21	0.22	0.22
500	53	HN	59	HA	0.45	0.37	0.33	0.34	0.34	0.34	0.33
501	53	HN	57	HA	0.45	0.30	0.37	0.39	0.40	0.38	0.37
502	53	HN	58	HN	0.45	0.27	0.31	0.32	0.33	0.31	0.31
503	53	HD@	53	HE@	1.13	0.28	0.28	0.28	0.28	0.28	0.28
504	53	HD@	53	HA	0.89	0.28	0.27	0.27	0.27	0.27	0.27
505	53	HD@	60	HN	0.89	0.59	0.54	0.55	0.52	0.54	0.53
506	53	HN	53	HD@	0.89	0.35	0.34	0.33	0.34	0.34	0.34
507	53	HE@	60	HN	0.89	0.59	0.61	0.58	0.61	0.62	0.60
508	53	HE@	60	HB@	0.94	0.45	0.47	0.47	0.46	0.48	0.47
509	53	HE@	66	HB@	1.09	0.36	0.56	0.53	0.48	0.54	0.48
510	53	HD@	59	HA	0.99	0.56	0.58	0.56	0.59	0.59	0.57
511	53	HN	57	HN	0.75	0.46	0.54	0.61	0.57	0.55	0.54
512	53	HN	60	HN	0.75	0.52	0.41	0.44	0.40	0.41	0.42
513	54	HN	54	HA@	0.65	0.24	0.24	0.24	0.24	0.24	0.24
514	54	HA@	55	HN	0.65	0.24	0.24	0.24	0.24	0.24	0.24
515	54	HN	55	HN	0.55	0.46	0.45	0.45	0.45	0.45	0.45
516	55	HN	55	HA	0.45	0.28	0.26	0.27	0.27	0.26	0.27
517	55	HA	55	HB	0.25	0.24	0.26	0.24	0.25	0.25	0.24
518	55	HN	55	HB	0.30	0.24	0.28	0.26	0.28	0.27	0.24
519	55	HN	55	HG1@	0.65	0.43	0.26	0.31	0.27	0.28	0.33
520	55	HG2@	55	HD@	1.05	0.36	0.36	0.38	0.36	0.37	0.37
521	55	HG2@	56	HA	0.60	0.48	0.52	0.53	0.55	0.54	0.50
522	55	HG2@	56	HN	0.60	0.32	0.40	0.35	0.39	0.37	0.34
523	55	HN	55	HG2@	0.60	0.29	0.34	0.30	0.32	0.31	0.30

524	55	HG2@	56	HD@	1.28	0.51	0.60	0.56	0.60	0.58	0.52
525	55	HG1@	56	HD@	1.03	0.61	0.57	0.57	0.54	0.54	0.55
526	55	HN	56	HN	0.55	0.27	0.29	0.28	0.29	0.30	0.30
527	55	HN	57	HN	0.55	0.40	0.42	0.53	0.47	0.42	0.43
528	55	HG2@	88	HD@	1.15	0.60	0.54	0.60	0.59	0.55	0.54
529	56	HN	56	HA	0.45	0.28	0.28	0.28	0.28	0.28	0.28
530	56	HN	56	HB@	0.65	0.30	0.29	0.28	0.29	0.28	0.29
531	56	HA	56	HB@	0.65	0.26	0.26	0.26	0.26	0.26	0.26
532	56	HA	56	HD@	0.98	0.34	0.35	0.35	0.35	0.34	0.34
533	56	HA	57	HN	0.45	0.31	0.31	0.25	0.28	0.31	0.30
534	56	HN	57	HN	0.30	0.24	0.26	0.33	0.29	0.26	0.25
535	56	HD@	91	HB@	1.03	0.58	0.51	0.54	0.50	0.52	0.52
536	56	HB@	56	HD@	1.03	0.26	0.26	0.26	0.26	0.26	0.26
537	56	HB@	56	HG	0.65	0.25	0.25	0.25	0.25	0.25	0.25
538	56	HD@	108	HE3	0.98	0.68	0.88	0.61	0.72	0.78	0.77
539	56	HD@	108	HZ2	0.98	0.45	0.51	0.47	0.48	0.61	0.63
540	56	HB@	108	HZ2	0.65	0.38	0.32	0.35	0.44	0.42	0.48
541	56	HD@	108	HE1	0.98	0.44	0.58	0.61	0.75	0.59	0.82
542	56	HB@	108	HE1	0.65	0.28	0.41	0.47	0.63	0.46	0.65
543	57	HN	57	HA	0.25	0.22	0.21	0.24	0.22	0.21	0.21
544	57	HN	57	HB@	0.65	0.36	0.36	0.30	0.33	0.35	0.34
545	57	HA	58	HN	0.30	0.25	0.22	0.23	0.24	0.23	0.23
546	57	HN	58	HN	0.45	0.30	0.31	0.35	0.32	0.31	0.31
547	57	HN	58	HG1@	0.65	0.50	0.47	0.50	0.47	0.49	0.49
548	57	HN	58	HD@	0.60	0.60	0.58	0.62	0.61	0.60	0.59
549	58	HN	58	HA	0.45	0.28	0.28	0.28	0.28	0.28	0.28
550	58	HN	58	HB	0.45	0.23	0.24	0.24	0.27	0.25	0.24
551	58	HA	59	HN	0.30	0.22	0.21	0.21	0.21	0.21	0.21
552	58	HG2@	63	HD1	0.75	0.38	0.37	0.48	0.38	0.40	0.35
553	58	HG2@	59	HN	0.75	0.32	0.37	0.40	0.41	0.40	0.38

554	58	HD@	95	HN	0.75	0.37	0.44	0.52	0.45	0.46	0.42
555	58	HN	59	HA	0.75	0.52	0.50	0.50	0.52	0.51	0.51
556	58	HN	60	HN	0.55	0.71	0.65	0.68	0.66	0.65	0.67
557	59	HN	59	HA	0.45	0.29	0.27	0.27	0.27	0.27	0.27
558	59	HN	59	HB2	0.30	0.26	0.23	0.23	0.25	0.25	0.25
559	59	HN	59	HB1	0.30	0.24	0.25	0.25	0.22	0.23	0.23
560	59	HA	59	HB2	0.30	0.25	0.25	0.25	0.23	0.24	0.24
561	59	HA	59	HB1	0.45	0.29	0.29	0.29	0.29	0.28	0.29
562	59	HA	60	HN	0.25	0.22	0.21	0.21	0.21	0.21	0.21
563	59	HB2	60	HN	0.45	0.39	0.41	0.40	0.40	0.39	0.39
564	59	HB1	60	HN	0.55	0.38	0.39	0.38	0.42	0.41	0.41
565	59	HB1	63	HD1	0.25	0.27	0.30	0.25	0.29	0.26	0.28
566	59	HA	61	HN	0.55	0.41	0.43	0.42	0.42	0.44	0.44
567	59	HA	63	HE1	0.75	0.70	0.68	0.62	0.62	0.63	0.61
568	59	HN	60	HN	0.55	0.46	0.46	0.46	0.45	0.46	0.46
569	60	HN	60	HA	0.30	0.28	0.26	0.26	0.26	0.26	0.26
570	60	HN	60	HB@	0.65	0.30	0.30	0.29	0.29	0.29	0.29
571	60	HA	60	HB@	0.45	0.23	0.23	0.23	0.23	0.23	0.23
572	60	HN	61	HN	0.45	0.28	0.29	0.29	0.30	0.30	0.30
573	60	HA	65	HA	0.75	0.47	0.55	0.48	0.58	0.56	0.54
574	60	HA	64	HA	0.55	0.48	0.52	0.54	0.52	0.51	0.51
575	60	HN	62	HN	0.75	0.52	0.44	0.53	0.47	0.46	0.46
576	60	HN	64	HN	0.75	0.49	0.50	0.51	0.52	0.49	0.50
577	61	HN	61	HA	0.45	0.29	0.27	0.28	0.28	0.27	0.28
578	61	HN	61	HB2	0.30	0.26	0.23	0.24	0.26	0.25	0.25
579	61	HN	61	HB1	0.30	0.26	0.28	0.31	0.23	0.23	0.23
580	61	HN	62	HN	0.30	0.28	0.27	0.32	0.26	0.28	0.26
581	61	HA	72	HA	0.30	0.31	0.29	0.32	0.30	0.33	0.30
582	61	HN	63	HN	0.55	0.37	0.47	0.45	0.44	0.45	0.45
583	62	HN	62	HA	0.45	0.27	0.27	0.23	0.27	0.28	0.27

584	62	HH2	62	HZ2	0.25	0.24	0.25	0.25	0.25	0.25	0.25
585	62	HZ3	62	HH2	0.45	0.25	0.25	0.25	0.25	0.25	0.25
586	63	HN	63	HA	0.45	0.28	0.28	0.27	0.28	0.28	0.28
587	63	HA	63	HB@	0.50	0.26	0.26	0.26	0.26	0.26	0.26
588	63	HE3	63	HZ3	0.25	0.25	0.25	0.25	0.25	0.25	0.25
589	63	HH2	63	HZ2	0.45	0.25	0.25	0.25	0.25	0.25	0.25
590	63	HE3	63	HB@	0.65	0.35	0.34	0.32	0.34	0.34	0.34
591	63	HD1	63	HB@	0.65	0.31	0.30	0.32	0.30	0.30	0.30
592	63	HE3	63	HA	0.30	0.25	0.29	0.32	0.28	0.28	0.28
593	63	HE3	76	HN	0.45	0.32	0.35	0.39	0.33	0.34	0.34
594	63	HZ3	75	HN	0.45	0.52	0.58	0.68	0.56	0.56	0.57
595	63	HZ3	63	HA	0.45	0.46	0.50	0.54	0.49	0.49	0.49
596	63	HB@	76	HA	0.65	0.62	0.56	0.64	0.57	0.58	0.56
597	63	HA	75	HN	0.30	0.24	0.31	0.29	0.28	0.27	0.28
598	63	HA	76	HN	0.45	0.29	0.31	0.31	0.28	0.28	0.29
599	63	HE3	75	HB2	0.30	0.24	0.27	0.34	0.26	0.27	0.26
600	63	HZ3	75	HB1	0.45	0.29	0.29	0.38	0.29	0.30	0.29
601	63	HZ3	75	HB2	0.45	0.27	0.36	0.45	0.33	0.34	0.34
602	63	HD1	98	HG1@	0.65	0.50	0.49	0.75	0.50	0.55	0.53
603	63	HE3	98	HG1@	0.65	0.34	0.35	0.45	0.39	0.40	0.38
604	63	HA	76	HB@	0.45	0.49	0.47	0.48	0.45	0.46	0.46
605	63	HZ2	98	HG2@	0.60	0.40	0.39	0.52	0.38	0.44	0.38
606	63	HZ2	98	HG1@	0.65	0.42	0.46	0.56	0.42	0.41	0.45
607	63	HE1	63	HB@	0.75	0.49	0.49	0.48	0.48	0.48	0.48
608	63	HE1	98	HG2@	0.85	0.48	0.43	0.66	0.44	0.53	0.44
609	63	HE1	98	HG1@	0.95	0.46	0.47	0.69	0.45	0.48	0.48
610	63	HD1	98	HD@	0.75	0.46	0.45	0.70	0.46	0.52	0.46
611	63	HE3	75	HD@	0.83	0.52	0.47	0.56	0.50	0.50	0.47
612	63	HZ3	75	HD@	0.98	0.44	0.41	0.54	0.42	0.43	0.41
613	63	HE3	75	HN	0.55	0.38	0.46	0.52	0.44	0.44	0.44

614	63	HN	75	HN	0.75	0.44	0.52	0.47	0.48	0.47	0.50
615	63	HZ3	101	HB@	0.75	0.38	0.39	0.56	0.44	0.50	0.55
616	63	HE3	98	HG2@	1.05	0.63	0.59	0.44	0.63	0.67	0.58
617	63	HH2	98	HG2@	0.85	0.47	0.47	0.46	0.46	0.47	0.45
618	64	HN	64	HA	0.30	0.29	0.28	0.28	0.28	0.28	0.28
619	64	HA	64	HB@	0.50	0.21	0.21	0.21	0.21	0.21	0.21
620	64	HA	78	HN	0.45	0.33	0.47	0.35	0.45	0.45	0.39
621	64	HB@	80	HN	0.65	0.41	0.42	0.41	0.41	0.42	0.42
622	64	HA	78	HG2@	0.60	0.65	0.70	0.52	0.65	0.61	0.65
623	64	HN	64	HB@	0.65	0.34	0.31	0.32	0.31	0.31	0.32
624	64	HA	65	HN	0.30	0.24	0.23	0.24	0.23	0.24	0.24
625	64	HA	76	HN	0.55	0.39	0.36	0.38	0.37	0.37	0.38
626	64	HA	77	HN	0.55	0.45	0.45	0.46	0.44	0.42	0.44
627	64	HA	65	HA	0.75	0.43	0.42	0.43	0.42	0.42	0.42
628	64	HA	74	HA	0.30	0.31	0.42	0.32	0.40	0.41	0.39
629	64	HA	74	HB@	0.75	0.47	0.62	0.50	0.51	0.54	0.59
630	65	HN	65	HA	0.45	0.29	0.28	0.28	0.28	0.28	0.28
631	65	HN	65	HB@	0.50	0.25	0.24	0.25	0.24	0.24	0.24
632	65	HA	65	HB@	0.65	0.26	0.25	0.25	0.25	0.25	0.25
633	65	HA	66	HN	0.30	0.22	0.21	0.22	0.22	0.22	0.22
634	65	HA	67	HN	0.45	0.34	0.46	0.51	0.43	0.39	0.38
635	65	HN	78	HG2@	0.75	0.68	0.68	0.59	0.65	0.62	0.67
636	65	HB@	66	HN	0.75	0.42	0.41	0.38	0.42	0.42	0.41
637	65	HN	66	HN	0.55	0.44	0.44	0.45	0.44	0.44	0.44
638	65	HN	78	HN	0.55	0.37	0.47	0.37	0.47	0.46	0.43
639	66	HN	66	HA	0.45	0.28	0.28	0.28	0.28	0.28	0.28
640	66	HN	66	HB1	0.45	0.30	0.26	0.26	0.29	0.27	0.29
641	66	HA	66	HB2	0.25	0.23	0.24	0.24	0.24	0.24	0.24
642	66	HA	66	HB1	0.25	0.24	0.28	0.28	0.24	0.24	0.24
643	66	HA	67	HN	0.45	0.32	0.25	0.23	0.27	0.32	0.33

644	66	HN	67	HN	0.30	0.22	0.30	0.33	0.27	0.25	0.22
645	66	HN	67	HA@	0.75	0.45	0.50	0.53	0.49	0.47	0.45
646	66	HN	68	HN	0.75	0.35	0.52	0.49	0.45	0.45	0.41
647	66	HN	69	HN	0.75	0.43	0.54	0.48	0.49	0.51	0.46
648	67	HN	67	HA@	0.65	0.24	0.24	0.23	0.24	0.24	0.24
649	67	HN	68	HA	0.55	0.52	0.52	0.52	0.51	0.52	0.51
650	68	HN	68	HA	0.45	0.28	0.28	0.28	0.28	0.28	0.28
651	68	HA	68	HB@	0.50	0.26	0.26	0.26	0.26	0.25	0.25
652	68	HN	68	HB@	0.65	0.32	0.28	0.28	0.28	0.29	0.28
653	68	HA	69	HN	0.45	0.31	0.34	0.34	0.35	0.34	0.33
654	69	HA	69	HB	0.45	0.30	0.26	0.26	0.29	0.28	0.28
655	69	HN	69	HB	0.45	0.26	0.29	0.30	0.27	0.28	0.27
656	69	HB	69	HG2@	0.55	0.24	0.24	0.24	0.24	0.24	0.24
657	71	HN	71	HA@	0.50	0.24	0.23	0.24	0.23	0.23	0.23
658	71	HA@	72	HN	0.65	0.31	0.31	0.30	0.30	0.31	0.31
659	71	HN	72	HN	0.30	0.27	0.28	0.28	0.28	0.28	0.28
660	72	HN	72	HA	0.45	0.28	0.27	0.27	0.27	0.27	0.27
661	72	HN	72	HB@	0.50	0.24	0.28	0.26	0.25	0.28	0.29
662	72	HA	72	HB@	0.50	0.26	0.25	0.25	0.25	0.25	0.25
663	72	HA	73	HN	0.45	0.21	0.22	0.22	0.22	0.22	0.22
664	72	HA	74	HN	0.55	0.36	0.41	0.39	0.39	0.42	0.40
665	74	HN	74	HA	0.25	0.22	0.20	0.21	0.20	0.21	0.20
666	74	HA	74	HB@	0.65	0.25	0.25	0.25	0.24	0.25	0.26
667	74	HA	75	HN	0.45	0.25	0.27	0.25	0.26	0.26	0.28
668	74	HA	76	HN	0.45	0.35	0.38	0.36	0.37	0.38	0.38
669	74	HN	74	HB@	0.65	0.31	0.35	0.31	0.34	0.35	0.35
670	74	HN	75	HN	0.45	0.32	0.29	0.30	0.30	0.29	0.29
671	74	HN	76	HN	0.75	0.52	0.51	0.53	0.52	0.53	0.51
672	75	HN	75	HA	0.45	0.28	0.27	0.27	0.27	0.27	0.27
673	75	HN	75	HB2	0.30	0.25	0.23	0.23	0.23	0.23	0.23

674	75	HN	75	HB1	0.45	0.36	0.34	0.30	0.34	0.34	0.34
675	75	HA	75	HB2	0.45	0.29	0.29	0.27	0.29	0.29	0.29
676	75	HA	75	HB1	0.45	0.24	0.26	0.27	0.26	0.26	0.26
677	75	HN	75	HG	0.30	0.24	0.24	0.28	0.24	0.24	0.24
678	75	HA	76	HN	0.45	0.34	0.34	0.34	0.34	0.34	0.34
679	75	HN	76	HN	0.45	0.26	0.29	0.29	0.29	0.30	0.30
680	75	HB2	76	HN	0.45	0.32	0.31	0.33	0.31	0.31	0.32
681	75	HN	75	HD@	0.98	0.40	0.40	0.42	0.40	0.39	0.40
682	75	HB1	76	HB@	0.50	0.63	0.57	0.52	0.58	0.57	0.57
683	75	HG	76	HN	0.75	0.46	0.47	0.44	0.48	0.48	0.48
684	75	HD@	76	HN	1.08	0.57	0.56	0.54	0.57	0.57	0.57
685	76	HN	76	HA	0.45	0.28	0.28	0.28	0.28	0.28	0.28
686	76	HN	76	HB@	0.50	0.29	0.27	0.27	0.28	0.28	0.28
687	76	HN	77	HN	0.30	0.26	0.27	0.27	0.26	0.26	0.26
688	76	HN	78	HG2@	0.75	0.71	0.77	0.63	0.77	0.74	0.74
689	76	HA	76	HB@	0.45	0.26	0.26	0.26	0.26	0.26	0.26
690	76	HA	77	HN	0.45	0.33	0.31	0.31	0.32	0.32	0.32
691	76	HB@	78	HG2@	0.75	0.49	0.56	0.43	0.55	0.50	0.52
692	76	HN	77	HA	0.55	0.46	0.45	0.46	0.45	0.44	0.44
693	76	HN	77	HB@	0.95	0.55	0.60	0.60	0.59	0.58	0.58
694	76	HN	78	HN	0.55	0.42	0.49	0.43	0.48	0.49	0.45
695	77	HA	77	HB@	0.45	0.26	0.25	0.26	0.26	0.25	0.25
696	77	HB@	78	HN	0.65	0.44	0.33	0.42	0.33	0.34	0.38
697	77	HN	77	HB@	0.65	0.33	0.35	0.35	0.35	0.34	0.34
698	77	HN	78	HN	0.45	0.29	0.35	0.30	0.35	0.35	0.33
699	77	HA	78	HN	0.30	0.27	0.34	0.26	0.33	0.34	0.30
700	78	HN	78	HA	0.45	0.29	0.27	0.28	0.27	0.27	0.28
701	78	HA	78	HB	0.25	0.26	0.25	0.25	0.26	0.25	0.25
702	78	HA	78	HG2@	0.55	0.29	0.28	0.29	0.28	0.29	0.28
703	78	HN	78	HG1@	0.50	0.25	0.24	0.29	0.25	0.25	0.24

704	78	HN	78	HG2@	0.75	0.42	0.31	0.38	0.33	0.31	0.34
705	79	HA	79	HB@	0.50	0.24	0.25	0.24	0.25	0.25	0.25
706	79	HA	80	HN	0.25	0.22	0.22	0.22	0.22	0.22	0.22
707	80	HN	80	HA	0.45	0.27	0.27	0.27	0.27	0.27	0.27
708	80	HA	83	HN	0.45	0.34	0.34	0.36	0.33	0.34	0.34
709	80	HN	81	HN	0.45	0.28	0.28	0.29	0.29	0.28	0.29
710	81	HN	81	HA	0.30	0.27	0.26	0.26	0.26	0.26	0.26
711	81	HN	82	HN	0.30	0.28	0.30	0.30	0.30	0.30	0.30
712	81	HA	84	HN	0.55	0.35	0.36	0.37	0.35	0.36	0.36
713	81	HN	83	HN	0.55	0.43	0.45	0.44	0.44	0.44	0.44
714	82	HN	82	HA	0.30	0.28	0.27	0.27	0.27	0.27	0.27
715	82	HN	82	HB@	0.55	0.28	0.27	0.27	0.27	0.27	0.27
716	82	HA	82	HB@	0.55	0.24	0.24	0.24	0.24	0.24	0.24
717	82	HN	83	HN	0.30	0.26	0.28	0.28	0.29	0.29	0.28
718	82	HB@	83	HN	0.75	0.35	0.35	0.36	0.35	0.35	0.36
719	82	HA	84	HN	0.55	0.36	0.40	0.37	0.42	0.40	0.38
720	83	HN	83	HA	0.30	0.27	0.27	0.27	0.27	0.27	0.27
721	83	HN	83	HB@	0.50	0.30	0.28	0.28	0.26	0.27	0.28
722	83	HA	83	HB@	0.50	0.26	0.26	0.26	0.26	0.26	0.26
723	83	HA	83	HD@	0.78	0.35	0.35	0.36	0.35	0.34	0.35
724	83	HA	84	HN	0.45	0.33	0.34	0.33	0.34	0.34	0.33
725	83	HN	84	HN	0.30	0.27	0.27	0.27	0.27	0.28	0.26
726	83	HN	83	HG	0.30	0.24	0.24	0.24	0.26	0.26	0.24
727	83	HN	83	HD@	0.98	0.40	0.39	0.39	0.40	0.39	0.39
728	84	HN	84	HA	0.45	0.29	0.27	0.28	0.27	0.27	0.28
729	84	HA	84	HB@	0.65	0.25	0.26	0.25	0.26	0.26	0.26
730	84	HN	84	HB@	0.50	0.30	0.28	0.29	0.28	0.27	0.28
731	84	HA	85	HN	0.45	0.31	0.31	0.26	0.30	0.31	0.29
732	85	HN	85	HA	0.30	0.28	0.27	0.28	0.27	0.27	0.27
733	85	HN	85	HB@	0.50	0.28	0.26	0.28	0.26	0.27	0.28

734	85	HA	85	HB@	0.50	0.26	0.25	0.25	0.26	0.25	0.25
735	85	HA	86	HN	0.25	0.23	0.23	0.24	0.23	0.24	0.24
736	86	HN	86	HA	0.30	0.27	0.27	0.26	0.25	0.26	0.27
737	86	HN	86	HB@	0.65	0.30	0.29	0.29	0.30	0.29	0.28
738	86	HA	86	HB@	0.45	0.26	0.25	0.25	0.25	0.24	0.24
739	86	HN	87	HN	0.30	0.26	0.29	0.29	0.30	0.30	0.30
740	87	HA	87	HB2	0.45	0.24	0.24	0.24	0.24	0.23	0.23
741	87	HA	87	HB1	0.30	0.29	0.29	0.29	0.29	0.28	0.29
742	87	HA	88	HN	0.25	0.23	0.22	0.22	0.22	0.22	0.22
743	87	HN	87	HA	0.45	0.28	0.27	0.28	0.28	0.28	0.28
744	87	HN	87	HB2	0.45	0.27	0.25	0.27	0.26	0.27	0.29
745	87	HN	87	HB1	0.45	0.24	0.24	0.26	0.24	0.24	0.24
746	88	HN	88	HG2@	0.60	0.33	0.33	0.35	0.33	0.32	0.33
747	88	HN	88	HD@	0.75	0.45	0.43	0.40	0.39	0.42	0.41
748	88	HG1@	89	HN	0.65	0.38	0.44	0.45	0.42	0.44	0.41
749	88	HG2@	89	HN	0.75	0.52	0.44	0.53	0.46	0.44	0.48
750	88	HD@	89	HN	0.75	0.59	0.54	0.58	0.49	0.55	0.54
751	88	HN	88	HG1@	0.50	0.26	0.29	0.23	0.28	0.28	0.26
752	88	HD@	88	HG1@	0.95	0.22	0.22	0.22	0.22	0.22	0.22
753	88	HD@	92	HG2@	0.85	0.33	0.47	0.38	0.43	0.43	0.42
754	88	HB	88	HD@	0.60	0.29	0.29	0.28	0.29	0.28	0.28
755	88	HD@	88	HG2@	0.85	0.35	0.39	0.36	0.39	0.39	0.37
756	88	HB	88	HG2@	0.60	0.24	0.24	0.24	0.24	0.24	0.24
757	88	HB	88	HG1@	0.65	0.25	0.25	0.25	0.25	0.25	0.25
758	89	HA	92	HN	0.45	0.38	0.35	0.36	0.35	0.35	0.35
759	89	HA	92	HB	0.30	0.32	0.33	0.32	0.30	0.28	0.31
760	89	HA	90	HN	0.45	0.35	0.35	0.35	0.35	0.35	0.35
761	89	HN	90	HN	0.45	0.31	0.29	0.30	0.29	0.29	0.30
762	89	HN	89	HG2@	0.75	0.44	0.33	0.33	0.42	0.41	0.42
763	89	HA	89	HG2@	0.55	0.31	0.28	0.28	0.28	0.29	0.29

764	89	HB	89	HG2@	0.55	0.24	0.24	0.24	0.24	0.24	0.24
765	89	HG2@	90	HN	0.75	0.39	0.46	0.46	0.45	0.44	0.43
766	89	HG2@	93	HN	0.75	0.51	0.61	0.63	0.50	0.50	0.51
767	90	HN	90	HA	0.25	0.28	0.27	0.27	0.27	0.27	0.27
768	90	HN	90	HB@	0.55	0.28	0.27	0.27	0.27	0.27	0.27
769	90	HA	90	HB@	0.55	0.24	0.24	0.24	0.24	0.24	0.24
770	90	HA	93	HN	0.45	0.34	0.36	0.35	0.36	0.36	0.36
771	90	HN	91	HN	0.30	0.27	0.29	0.28	0.29	0.29	0.29
772	90	HB@	91	HN	0.60	0.34	0.32	0.33	0.32	0.32	0.32
773	91	HN	91	HA	0.30	0.27	0.26	0.26	0.26	0.26	0.26
774	91	HN	91	HB@	0.65	0.29	0.27	0.29	0.29	0.29	0.29
775	91	HA	92	HN	0.45	0.35	0.35	0.35	0.35	0.35	0.35
776	91	HA	94	HN	0.45	0.35	0.37	0.35	0.36	0.36	0.36
777	91	HB@	92	HN	0.65	0.31	0.30	0.31	0.30	0.30	0.31
778	91	HN	92	HN	0.30	0.28	0.30	0.30	0.30	0.30	0.30
779	92	HN	92	HA	0.30	0.28	0.27	0.27	0.27	0.27	0.27
780	92	HN	92	HB	0.25	0.24	0.25	0.24	0.23	0.22	0.23
781	92	HA	92	HB	0.30	0.29	0.27	0.28	0.28	0.28	0.28
782	92	HA	92	HG2@	0.55	0.29	0.30	0.29	0.28	0.28	0.29
783	92	HA	92	HG1@	0.55	0.31	0.30	0.31	0.31	0.31	0.31
784	92	HN	92	HG2@	0.55	0.31	0.30	0.29	0.30	0.30	0.30
785	92	HN	92	HG1@	0.75	0.44	0.38	0.42	0.42	0.41	0.40
786	92	HA	95	HN	0.30	0.34	0.34	0.34	0.34	0.35	0.34
787	92	HN	93	HN	0.30	0.27	0.29	0.29	0.29	0.29	0.28
788	92	HA	95	HB@	0.55	0.34	0.33	0.33	0.32	0.33	0.33
789	92	HG2@	93	HN	0.75	0.47	0.41	0.47	0.47	0.46	0.45
790	92	HB	93	HN	0.30	0.28	0.28	0.26	0.28	0.27	0.27
791	92	HG1@	93	HN	0.75	0.41	0.40	0.40	0.40	0.38	0.39
792	92	HG1@	96	HA	0.60	0.62	0.69	0.67	0.67	0.69	0.69
793	92	HB	92	HG2@	0.55	0.23	0.24	0.24	0.24	0.24	0.24

794	92	HB	92	HG1@	0.60	0.24	0.24	0.24	0.24	0.24	0.24
795	92	HN	94	HN	0.55	0.42	0.44	0.44	0.44	0.44	0.43
796	93	HN	93	HA	0.25	0.28	0.27	0.26	0.27	0.27	0.27
797	93	HN	93	HB2	0.30	0.24	0.23	0.23	0.23	0.23	0.23
798	93	HN	93	HB1	0.25	0.35	0.32	0.33	0.33	0.34	0.34
799	93	HA	93	HB2	0.30	0.29	0.28	0.28	0.28	0.29	0.29
800	93	HA	93	HB1	0.25	0.25	0.26	0.25	0.26	0.25	0.25
801	93	HB2	94	HN	0.45	0.29	0.28	0.28	0.29	0.28	0.28
802	93	HA	96	HN	0.45	0.34	0.37	0.36	0.36	0.36	0.36
803	93	HN	94	HN	0.30	0.27	0.30	0.30	0.30	0.30	0.30
804	94	HN	94	HA	0.30	0.28	0.27	0.27	0.27	0.27	0.27
805	94	HN	94	HB2	0.30	0.25	0.23	0.23	0.23	0.23	0.23
806	94	HN	94	HB1	0.30	0.24	0.25	0.25	0.24	0.24	0.24
807	94	HA	94	HB2	0.30	0.23	0.24	0.24	0.23	0.24	0.23
808	94	HA	95	HN	0.45	0.35	0.35	0.35	0.35	0.35	0.35
809	94	HB1	95	HN	0.30	0.27	0.26	0.26	0.26	0.27	0.27
810	94	HA	97	HN	0.45	0.31	0.34	0.33	0.34	0.34	0.34
811	94	HN	95	HN	0.30	0.27	0.29	0.29	0.29	0.29	0.29
812	95	HN	95	HA	0.30	0.27	0.26	0.26	0.26	0.26	0.26
813	95	HN	95	HB@	0.55	0.27	0.27	0.27	0.27	0.27	0.27
814	95	HA	95	HB@	0.55	0.24	0.24	0.24	0.24	0.24	0.24
815	95	HN	96	HN	0.30	0.28	0.30	0.30	0.30	0.30	0.30
816	95	HN	97	HN	0.45	0.42	0.45	0.43	0.44	0.45	0.45
817	95	HB@	96	HN	0.60	0.33	0.33	0.32	0.33	0.33	0.33
818	95	HB@	108	HH2	0.55	0.35	0.61	0.53	0.44	0.61	0.61
819	95	HB@	108	HZ2	0.60	0.34	0.67	0.71	0.68	0.73	0.77
820	95	HA	108	HZ2	0.30	0.27	0.59	0.61	0.72	0.62	0.68
821	95	HN	98	HG2@	1.05	0.76	0.74	0.68	0.76	0.76	0.73
822	96	HN	96	HA	0.30	0.27	0.26	0.26	0.26	0.26	0.26
823	96	HN	96	HB@	0.45	0.29	0.28	0.28	0.28	0.28	0.27

824	96	HA	96	HB@	0.50	0.26	0.26	0.25	0.26	0.26	0.26
825	96	HN	97	HN	0.30	0.28	0.29	0.29	0.30	0.30	0.30
826	97	HN	97	HA	0.30	0.28	0.27	0.27	0.27	0.27	0.27
827	97	HA	98	HN	0.45	0.35	0.35	0.35	0.35	0.35	0.35
828	97	HN	98	HN	0.30	0.28	0.28	0.27	0.27	0.27	0.27
829	97	HN	99	HN	0.45	0.43	0.46	0.45	0.45	0.45	0.45
830	98	HN	98	HB	0.30	0.24	0.24	0.28	0.25	0.24	0.26
831	98	HN	98	HG2@	0.75	0.43	0.43	0.35	0.43	0.43	0.41
832	98	HN	98	HG1@	0.50	0.28	0.26	0.26	0.25	0.26	0.25
833	98	HN	98	HD@	0.75	0.40	0.40	0.41	0.40	0.39	0.41
834	98	HN	99	HN	0.45	0.28	0.30	0.29	0.30	0.29	0.29
835	98	HG2@	99	HA	0.75	0.42	0.44	0.53	0.46	0.45	0.47
836	98	HB	99	HN	0.30	0.26	0.25	0.32	0.26	0.26	0.26
837	98	HG2@	108	HZ3	0.75	0.40	0.30	0.44	0.50	0.39	0.48
838	98	HG2@	108	HZ2	0.75	0.50	0.59	0.63	0.71	0.55	0.54
839	98	HN	98	HA	0.45	0.27	0.27	0.27	0.27	0.27	0.27
840	98	HG2@	99	HN	0.75	0.40	0.40	0.46	0.42	0.41	0.42
841	98	HA	98	HG2@	0.55	0.31	0.30	0.29	0.29	0.30	0.29
842	98	HG2@	98	HD@	0.90	0.36	0.35	0.36	0.35	0.35	0.35
843	98	HB	98	HG2@	0.60	0.23	0.24	0.24	0.24	0.24	0.24
844	98	HD@	108	HZ2	0.60	0.31	0.44	0.43	0.65	0.40	0.47
845	98	HG2@	108	HE1	0.85	0.67	0.80	0.78	0.76	0.58	0.70
846	98	HG1@	99	HN	0.75	0.46	0.46	0.35	0.45	0.46	0.42
847	98	HG2@	99	HB	1.05	0.57	0.63	0.71	0.62	0.60	0.64
848	98	HG2@	107	HN	0.85	0.51	0.61	0.71	0.65	0.63	0.68
849	98	HG2@	108	HE3	0.85	0.54	0.49	0.61	0.38	0.39	0.66
850	99	HN	99	HA	0.45	0.28	0.27	0.27	0.27	0.27	0.27
851	99	HN	99	HB	0.25	0.23	0.34	0.29	0.28	0.27	0.30
852	99	HA	99	HB	0.25	0.29	0.25	0.25	0.24	0.25	0.24
853	99	HN	99	HG@	0.83	0.34	0.26	0.27	0.29	0.30	0.27

854	99	HN	100	HN	0.45	0.24	0.28	0.30	0.29	0.30	0.29
855	99	HA	100	HN	0.45	0.32	0.34	0.32	0.34	0.28	0.34
856	99	HB	100	HN	0.45	0.27	0.38	0.38	0.36	0.33	0.36
857	99	HG@	108	HZ3	0.83	0.42	0.73	0.82	0.51	0.78	0.70
858	99	HB	108	HZ3	0.45	0.50	0.82	0.84	0.61	0.74	0.76
859	99	HN	108	HZ3	0.55	0.47	0.65	0.74	0.50	0.67	0.70
860	100	HN	100	HA	0.30	0.28	0.26	0.27	0.27	0.27	0.27
861	100	HN	100	HB@	0.65	0.30	0.28	0.29	0.28	0.29	0.28
862	100	HA	100	HB@	0.65	0.23	0.25	0.25	0.25	0.25	0.26
863	100	HN	101	HN	0.45	0.26	0.28	0.27	0.28	0.30	0.28
864	101	HN	101	HA	0.45	0.28	0.28	0.28	0.28	0.27	0.27
865	101	HN	101	HB@	0.45	0.28	0.23	0.24	0.24	0.26	0.29
866	101	HA	101	HB@	0.45	0.25	0.26	0.26	0.26	0.25	0.25
867	101	HA	102	HN	0.45	0.31	0.23	0.23	0.22	0.22	0.27
868	102	HN	102	HA@	0.65	0.25	0.23	0.23	0.23	0.23	0.24
869	104	HN	104	HA@	0.65	0.24	0.23	0.24	0.23	0.24	0.24
870	104	HN	105	HN	0.45	0.46	0.46	0.38	0.32	0.31	0.30
871	104	HA@	105	HN	0.65	0.24	0.25	0.27	0.28	0.28	0.27
872	105	HN	105	HA	0.45	0.27	0.28	0.27	0.26	0.27	0.22
873	105	HN	105	HB@	0.65	0.30	0.28	0.28	0.27	0.26	0.30
874	105	HA	105	HB@	0.50	0.26	0.26	0.25	0.26	0.26	0.26
875	105	HA	105	HE@	0.45	0.45	0.48	0.52	0.48	0.50	0.48
876	105	HB@	108	HE3	0.65	0.37	0.39	0.39	0.57	0.52	0.46
877	105	HA	108	HE3	0.25	0.22	0.26	0.30	0.35	0.36	0.29
878	105	HA	108	HZ3	0.30	0.26	0.29	0.36	0.52	0.44	0.34
879	105	HE@	105	HB@	0.65	0.35	0.35	0.39	0.39	0.36	0.42
880	105	HB@	111	HZ2	0.65	0.43	0.49	0.51	0.54	0.68	0.59
881	105	HB@	108	HZ3	0.75	0.46	0.50	0.43	0.69	0.66	0.50
882	105	HB@	111	HE1	0.75	0.44	0.46	0.55	0.64	0.75	0.64
883	105	HE@	111	HE1	0.70	0.45	0.57	0.46	0.60	0.70	0.55

884	105	HB@	106	HN	0.75	0.37	0.42	0.37	0.30	0.32	0.29
885	106	HN	106	HB@	0.65	0.28	0.28	0.26	0.26	0.28	0.28
886	106	HA	106	HB@	0.50	0.28	0.23	0.25	0.26	0.26	0.26
887	106	HA	107	HN	0.45	0.34	0.34	0.34	0.34	0.35	0.34
888	106	HN	107	HN	0.30	0.29	0.30	0.29	0.28	0.29	0.28
889	107	HN	107	HA	0.30	0.27	0.27	0.26	0.27	0.27	0.27
890	107	HN	107	HB@	0.55	0.27	0.27	0.26	0.27	0.27	0.26
891	107	HA	107	HB@	0.55	0.24	0.24	0.25	0.24	0.24	0.25
892	107	HA	108	HN	0.45	0.34	0.34	0.33	0.34	0.34	0.32
893	107	HB@	108	HN	0.75	0.36	0.37	0.38	0.34	0.34	0.38
894	107	HB@	108	HE3	0.60	0.41	0.40	0.50	0.70	0.39	0.59
895	107	HN	108	HN	0.30	0.28	0.29	0.30	0.27	0.29	0.31
896	107	HN	109	HN	0.75	0.66	0.68	0.67	0.64	0.58	0.69
897	108	HN	108	HB@	0.65	0.29	0.27	0.28	0.25	0.27	0.28
898	108	HH2	108	HZ2	0.30	0.25	0.25	0.25	0.25	0.25	0.25
899	108	HE3	108	HZ3	0.30	0.25	0.25	0.25	0.25	0.25	0.25
900	108	HZ3	108	HH2	0.30	0.25	0.25	0.25	0.25	0.25	0.25
901	108	HE3	108	HB@	0.65	0.33	0.33	0.29	0.28	0.31	0.28
902	108	HA	108	HD1	0.45	0.31	0.33	0.27	0.25	0.31	0.26
903	108	HN	108	HE3	0.45	0.26	0.23	0.33	0.44	0.27	0.37
904	108	HN	108	HZ3	0.55	0.47	0.47	0.56	0.68	0.51	0.60
905	109	HA	109	HB	0.25	0.23	0.27	0.27	0.26	0.26	0.26
906	109	HA	112	HN	0.30	0.36	0.36	0.35	0.39	0.36	0.35
907	109	HN	109	HB	0.30	0.25	0.27	0.25	0.25	0.26	0.25
908	109	HN	110	HN	0.30	0.28	0.28	0.28	0.25	0.28	0.28
909	109	HN	111	HN	0.45	0.42	0.43	0.43	0.48	0.45	0.44
910	109	HN	109	HG@	0.83	0.35	0.30	0.31	0.32	0.31	0.31
911	109	HG@	110	HN	0.98	0.35	0.40	0.40	0.41	0.40	0.40
912	109	HB	110	HN	0.30	0.40	0.28	0.28	0.32	0.29	0.30
913	109	HA	110	HN	0.45	0.35	0.35	0.35	0.33	0.35	0.35

914	109	HN	112	HN	0.55	0.50	0.50	0.50	0.56	0.50	0.50
915	110	HN	110	HA	0.30	0.28	0.27	0.26	0.26	0.27	0.26
916	110	HA	110	HB@	0.55	0.24	0.24	0.24	0.24	0.24	0.24
917	110	HA	113	HB@	0.50	0.35	0.39	0.41	0.37	0.46	0.44
918	110	HN	110	HB@	0.55	0.28	0.27	0.27	0.27	0.27	0.27
919	110	HN	111	HN	0.30	0.26	0.29	0.29	0.31	0.29	0.30
920	111	HN	111	HA	0.30	0.28	0.27	0.28	0.27	0.27	0.28
921	111	HN	112	HN	0.25	0.28	0.27	0.26	0.28	0.28	0.27
922	111	HE3	111	HZ3	0.25	0.25	0.25	0.25	0.25	0.25	0.25
923	111	HZ3	111	HH2	0.30	0.25	0.25	0.25	0.25	0.25	0.25
924	111	HE3	112	HA	0.30	0.26	0.35	0.35	0.40	0.36	0.37
925	111	HE3	112	HB@	0.65	0.44	0.47	0.43	0.44	0.37	0.45
926	111	HE1	116	HA	0.55	0.41	0.39	0.41	0.86	0.59	0.44
927	111	HZ2	116	HB@	0.75	0.44	0.74	0.58	1.05	0.89	0.57
928	112	HN	112	HA	0.30	0.26	0.26	0.27	0.28	0.27	0.27
929	112	HN	112	HB@	0.45	0.31	0.28	0.27	0.28	0.25	0.27
930	112	HN	113	HN	0.45	0.28	0.30	0.30	0.27	0.29	0.30
931	112	HB@	113	HN	0.50	0.32	0.29	0.29	0.31	0.29	0.28
932	112	HA	116	HB@	0.50	0.27	0.70	0.42	0.63	0.60	0.37
933	112	HN	116	HB@	0.95	0.51	0.89	0.66	0.90	0.85	0.61
934	113	HN	113	HA	0.45	0.28	0.27	0.27	0.27	0.27	0.27
935	113	HN	113	HB@	0.45	0.28	0.27	0.28	0.26	0.28	0.28
936	113	HA	113	HB@	0.45	0.26	0.26	0.26	0.26	0.26	0.26
937	113	HB@	114	HN	0.50	0.33	0.30	0.31	0.33	0.30	0.32
938	113	HN	114	HN	0.45	0.22	0.26	0.28	0.33	0.27	0.27
939	114	HN	114	HA	0.30	0.28	0.27	0.28	0.22	0.27	0.28
940	114	HA	114	HB@	0.50	0.25	0.25	0.26	0.26	0.26	0.26
941	114	HN	114	HB@	0.50	0.35	0.30	0.29	0.32	0.28	0.29
942	114	HN	115	HN	0.45	0.23	0.24	0.24	0.30	0.27	0.25
943	115	HN	115	HA	0.45	0.28	0.27	0.28	0.27	0.28	0.28

944	115	HN	115	HB@	0.65	0.30	0.28	0.29	0.29	0.28	0.29
945	115	HA	115	HB@	0.50	0.25	0.25	0.25	0.24	0.25	0.25
946	116	HN	116	HA	0.30	0.27	0.28	0.27	0.28	0.28	0.27
947	116	HN	116	HB@	0.45	0.23	0.28	0.27	0.29	0.28	0.26
948	116	HA	116	HB@	0.50	0.25	0.26	0.26	0.25	0.26	0.25
949	116	HA	117	HN	0.25	0.22	0.25	0.25	0.23	0.23	0.24
950	116	HB@	117	HN	0.65	0.41	0.33	0.32	0.32	0.34	0.34
951	116	HN	117	HN	0.55	0.46	0.31	0.34	0.40	0.43	0.38
952	117	HN	117	HA@	0.50	0.24	0.24	0.24	0.23	0.24	0.24
953	117	HN	118	HN	0.30	0.27	0.36	0.30	0.30	0.34	0.29
954	118	HN	118	HA	0.45	0.29	0.27	0.27	0.27	0.27	0.28
955	118	HN	118	HB	0.45	0.37	0.35	0.35	0.35	0.32	0.35
956	118	HA	118	HB	0.25	0.24	0.26	0.25	0.26	0.24	0.25
957	118	HA	119	HN	0.30	0.25	0.29	0.23	0.25	0.23	0.24
958	118	HN	118	HG2@	0.60	0.34	0.34	0.37	0.33	0.31	0.36
959	118	HB	118	HG2@	0.55	0.23	0.24	0.24	0.24	0.24	0.24
960	118	HG2@	119	HN	0.75	0.44	0.47	0.43	0.48	0.43	0.44
961	119	HN	119	HA	0.30	0.28	0.27	0.28	0.28	0.27	0.28
962	119	HA	119	HB2	0.25	0.24	0.25	0.24	0.24	0.24	0.24
963	119	HA	119	HB1	0.25	0.29	0.28	0.29	0.29	0.29	0.29
964	119	HA	120	HN	0.25	0.24	0.22	0.22	0.22	0.21	0.21
965	119	HN	119	HB2	0.45	0.26	0.24	0.25	0.26	0.25	0.26
966	119	HN	119	HB1	0.30	0.25	0.26	0.25	0.23	0.24	0.24
967	119	HA	121	HN	0.45	0.34	0.40	0.40	0.41	0.40	0.40
968	120	HN	120	HA	0.30	0.28	0.28	0.27	0.27	0.27	0.27
969	120	HA	120	HB	0.45	0.24	0.25	0.25	0.25	0.25	0.25
970	120	HN	121	HN	0.30	0.27	0.26	0.28	0.28	0.28	0.28
971	120	HN	120	HG@	0.83	0.26	0.33	0.29	0.30	0.30	0.27
972	120	HG@	121	HN	0.98	0.41	0.41	0.39	0.39	0.40	0.41
973	120	HA	123	HE1	0.45	0.45	0.45	0.56	0.57	0.56	0.52

974	121	HN	121	HA	0.30	0.28	0.27	0.27	0.27	0.27	0.28
975	121	HA	121	HB@	0.50	0.25	0.26	0.26	0.26	0.26	0.26
976	121	HN	121	HB@	0.50	0.24	0.27	0.27	0.27	0.27	0.27
977	121	HA	122	HN	0.45	0.34	0.35	0.35	0.35	0.35	0.35
978	121	HN	122	HN	0.30	0.27	0.29	0.27	0.27	0.27	0.26
979	121	HN	123	HN	0.55	0.44	0.44	0.42	0.41	0.42	0.42
980	121	HN	123	HD1	0.55	0.55	0.51	0.58	0.56	0.63	0.56
981	122	HN	122	HA	0.30	0.27	0.27	0.27	0.27	0.27	0.26
982	122	HA	122	HB@	0.55	0.24	0.24	0.24	0.24	0.24	0.24
983	122	HN	122	HB@	0.55	0.27	0.27	0.27	0.27	0.27	0.27
984	122	HA	123	HN	0.45	0.35	0.35	0.35	0.35	0.35	0.35
985	122	HB@	123	HN	0.60	0.36	0.33	0.33	0.33	0.33	0.33
986	123	HN	123	HA	0.30	0.28	0.27	0.27	0.27	0.27	0.26
987	123	HN	123	HB2	0.45	0.23	0.24	0.22	0.22	0.22	0.22
988	123	HN	123	HB1	0.45	0.35	0.35	0.33	0.34	0.34	0.34
989	123	HA	123	HB2	0.45	0.29	0.29	0.28	0.29	0.28	0.29
990	123	HA	123	HB1	0.30	0.26	0.25	0.26	0.26	0.26	0.26
991	123	HB1	124	HN	0.45	0.40	0.36	0.34	0.34	0.33	0.34
992	123	HH2	123	HZ2	0.25	0.25	0.25	0.25	0.25	0.25	0.25
993	123	HE3	123	HZ3	0.25	0.25	0.25	0.25	0.25	0.25	0.25
994	123	HE3	123	HB2	0.45	0.40	0.39	0.39	0.37	0.32	0.39
995	123	HE3	123	HB1	0.45	0.26	0.27	0.25	0.25	0.28	0.26
996	123	HE3	123	HA	0.30	0.29	0.28	0.34	0.36	0.35	0.31
997	123	HZ2	123	HE3	0.45	0.50	0.49	0.49	0.49	0.49	0.49
998	123	HE1	123	HB2	0.55	0.48	0.47	0.48	0.48	0.49	0.47
999	124	HN	124	HA	0.45	0.28	0.27	0.28	0.27	0.27	0.28
1000	124	HA	124	HB	0.25	0.24	0.25	0.25	0.25	0.25	0.25
1001	124	HN	124	HG1@	0.50	0.26	0.25	0.25	0.28	0.27	0.25
1002	124	HN	124	HG2@	0.60	0.35	0.33	0.34	0.32	0.32	0.35
1003	124	HN	125	HN	0.25	0.22	0.29	0.26	0.28	0.29	0.26

1004	124	HB	124	HD@	0.60	0.28	0.28	0.28	0.28	0.28	0.28
1005	124	HB	124	HG2@	0.55	0.24	0.24	0.24	0.24	0.24	0.24
1006	125	HN	125	HA	0.30	0.28	0.27	0.27	0.27	0.28	0.27
1007	125	HN	125	HB@	0.45	0.23	0.27	0.27	0.27	0.27	0.26
1008	125	HA	125	HB@	0.50	0.25	0.26	0.26	0.26	0.25	0.25
1009	125	HB@	126	HN	0.65	0.41	0.35	0.31	0.31	0.32	0.34
1010	125	HA	126	HN	0.25	0.22	0.23	0.27	0.27	0.26	0.23
1011	125	HN	126	HN	0.55	0.46	0.40	0.33	0.31	0.32	0.40
1012	126	HN	126	HA@	0.50	0.24	0.24	0.24	0.24	0.24	0.24
1013	126	HN	127	HN	0.30	0.27	0.32	0.36	0.34	0.33	0.32
1014	127	HN	127	HA	0.30	0.28	0.26	0.27	0.27	0.27	0.25
1015	127	HN	127	HB2	0.45	0.24	0.24	0.24	0.25	0.24	0.26
1016	127	HN	127	HB1	0.45	0.35	0.35	0.35	0.35	0.34	0.36
1017	127	HA	127	HB2	0.25	0.29	0.29	0.29	0.29	0.29	0.29
1018	127	HA	127	HB1	0.30	0.24	0.24	0.24	0.24	0.24	0.24
1019	127	HB1	128	HN	0.45	0.29	0.36	0.36	0.33	0.37	0.35
1020	127	HN	128	HN	0.45	0.46	0.29	0.28	0.29	0.31	0.30
1021	127	HA	128	HN	0.30	0.22	0.30	0.29	0.29	0.26	0.27
1022	127	HB1	129	HD@	0.98	0.41	0.76	0.81	0.76	0.65	0.60
1023	127	HA	129	HN	0.75	0.58	0.50	0.44	0.48	0.55	0.53
1024	128	HN	128	HA	0.45	0.28	0.27	0.28	0.26	0.28	0.28
1025	128	HA	128	HB@	0.45	0.25	0.25	0.26	0.25	0.24	0.24
1026	128	HA	129	HN	0.25	0.23	0.25	0.26	0.24	0.23	0.23
1027	128	HN	129	HB@	0.65	0.64	0.58	0.54	0.57	0.61	0.59
1028	129	HN	129	HA	0.30	0.29	0.28	0.28	0.28	0.28	0.28
1029	129	HA	129	HB@	0.50	0.25	0.26	0.26	0.26	0.26	0.25
1030	129	HA	129	HD@	0.78	0.35	0.35	0.35	0.34	0.34	0.35
1031	129	HN	129	HB@	0.65	0.32	0.29	0.29	0.29	0.29	0.29
1032	129	HN	129	HD@	0.98	0.46	0.45	0.47	0.46	0.44	0.45
1033	129	HN	129	HG	0.30	0.30	0.32	0.34	0.33	0.32	0.31

1034	129	HB@	129	HG	0.45	0.25	0.25	0.25	0.25	0.25	0.25
1035	1	HB@	1	HG@	0.70	0.24	0.23	0.23	0.23	0.23	0.23
1036	1	HB@	1	HA	0.50	0.25	0.25	0.25	0.25	0.25	0.25
1037	1	HB@	86	HA	0.65	0.34	0.42	0.37	0.44	0.41	0.39
1038	1	HG@	1	HA	0.50	0.28	0.27	0.27	0.28	0.27	0.27
1039	1	HD@	1	HE@	0.85	0.22	0.24	0.24	0.24	0.24	0.24
1040	1	HD@	1	HG@	0.70	0.24	0.24	0.24	0.24	0.24	0.24
1041	1	HE@	1	HG@	0.70	0.31	0.27	0.27	0.27	0.27	0.27
1042	1	HE@	86	HA	0.65	0.64	0.61	0.58	0.64	0.63	0.59
1043	2	HA	39	HB2	0.45	0.39	0.38	0.41	0.36	0.36	0.39
1044	2	HA	40	HG2@	0.75	0.53	0.57	0.53	0.57	0.57	0.54
1045	2	HG1@	38	HB2	0.75	0.34	0.37	0.38	0.34	0.34	0.33
1046	2	HG1@	39	HB2	0.75	0.41	0.43	0.41	0.42	0.43	0.44
1047	2	HG2@	39	HB1	0.75	0.50	0.53	0.59	0.50	0.50	0.51
1048	2	HG2@	39	HB2	0.60	0.36	0.37	0.42	0.35	0.35	0.36
1049	2	HG2@	1	HA	0.75	0.47	0.49	0.48	0.47	0.47	0.47
1050	3	HB1	38	HB1	0.45	0.34	0.36	0.45	0.37	0.36	0.38
1051	3	HB1	8	HB2	0.45	0.23	0.28	0.29	0.28	0.27	0.28
1052	3	HB1	7	HB@	0.65	0.41	0.47	0.37	0.40	0.41	0.38
1053	3	HB2	8	HB1	0.45	0.44	0.52	0.54	0.52	0.51	0.53
1054	3	HB2	8	HB2	0.45	0.29	0.39	0.40	0.38	0.36	0.37
1055	3	HB2	38	HB1	0.45	0.22	0.26	0.33	0.27	0.26	0.28
1056	4	HA@	7	HB@	0.85	0.48	0.44	0.45	0.45	0.47	0.45
1057	5	HA	5	HB@	0.50	0.22	0.25	0.25	0.25	0.25	0.25
1058	5	HA	5	HD@	0.65	0.46	0.45	0.44	0.45	0.45	0.44
1059	6	HB1	127	HB2	0.45	0.40	0.50	0.52	0.52	0.51	0.49
1060	6	HB1	9	HB@	0.75	0.50	0.55	0.53	0.56	0.57	0.53
1061	6	HB2	9	HB@	0.75	0.59	0.63	0.62	0.63	0.64	0.61
1062	7	HB@	7	HG@	0.70	0.22	0.23	0.23	0.23	0.23	0.23
1063	9	HB@	12	HB1	0.75	0.67	0.68	0.65	0.65	0.64	0.63

1064	12	HA	12	HG@	0.65	0.25	0.26	0.25	0.25	0.25	0.27
1065	12	HA	12	HB1	0.30	0.25	0.25	0.25	0.25	0.25	0.26
1066	12	HA	12	HB2	0.30	0.29	0.29	0.29	0.29	0.28	0.28
1067	12	HB2	17	HD@	0.98	0.59	0.56	0.58	0.60	0.66	0.65
1068	12	HB2	25	HD@	0.98	0.38	0.39	0.39	0.37	0.36	0.40
1069	12	HB2	12	HG@	0.50	0.25	0.24	0.24	0.24	0.24	0.24
1070	12	HG@	88	HD@	0.95	0.30	0.42	0.33	0.37	0.37	0.40
1071	12	HG@	17	HD@	1.18	0.53	0.55	0.53	0.52	0.58	0.53
1072	12	HE@	17	HG	0.45	0.62	0.61	0.64	0.66	0.61	0.63
1073	12	HE@	28	HA	0.60	0.50	0.59	0.54	0.56	0.55	0.54
1074	12	HE@	28	HB1	0.60	0.33	0.37	0.34	0.36	0.34	0.34
1075	12	HE@	28	HB2	0.60	0.46	0.37	0.38	0.34	0.39	0.38
1076	12	HE@	28	HE3	0.60	0.44	0.61	0.52	0.55	0.49	0.44
1077	12	HE@	28	HD1	0.60	0.68	0.47	0.52	0.45	0.54	0.51
1078	13	HA	13	HG@	0.50	0.27	0.27	0.26	0.27	0.28	0.28
1079	13	HA	13	HD@	0.65	0.43	0.35	0.38	0.36	0.38	0.39
1080	13	HA	13	HE@	0.65	0.51	0.48	0.48	0.49	0.51	0.50
1081	13	HA	25	HD@	0.98	0.40	0.44	0.45	0.43	0.39	0.42
1082	14	HA	14	HB@	0.50	0.26	0.26	0.26	0.26	0.26	0.26
1083	14	HA	14	HD@	0.65	0.44	0.38	0.38	0.40	0.40	0.40
1084	15	HA	92	HG1@	0.75	0.51	0.57	0.54	0.63	0.54	0.55
1085	15	HB1	92	HG2@	0.75	0.40	0.38	0.40	0.49	0.41	0.44
1086	15	HB1	92	HG1@	0.75	0.30	0.36	0.34	0.41	0.35	0.35
1087	17	HA	20	HB2	0.30	0.22	0.49	0.55	0.43	0.33	0.33
1088	17	HB2	12	HB2	0.45	0.40	0.44	0.47	0.47	0.50	0.46
1089	17	HD@	12	HB1	0.98	0.43	0.41	0.43	0.45	0.51	0.51
1090	20	HB1	17	HD@	0.98	0.48	0.71	0.71	0.63	0.54	0.54
1091	20	HB1	17	HA	0.45	0.29	0.54	0.60	0.47	0.34	0.33
1092	21	HA	21	HG@	0.65	0.32	0.27	0.27	0.27	0.27	0.28
1093	21	HA	21	HD@	0.65	0.25	0.35	0.32	0.31	0.31	0.30

1094	21	HB@	21	HG@	0.70	0.23	0.23	0.23	0.23	0.23	0.23
1095	21	HB@	21	HD@	0.85	0.30	0.28	0.28	0.28	0.28	0.29
1096	24	HA	24	HB1	0.25	0.25	0.28	0.27	0.28	0.27	0.26
1097	24	HA	24	HB2	0.25	0.24	0.24	0.25	0.25	0.25	0.25
1098	24	HA	19	HB@	0.65	0.44	0.53	0.46	0.50	0.52	0.52
1099	25	HA	28	HB@	0.65	0.38	0.41	0.41	0.44	0.40	0.42
1100	25	HA	28	HD1	0.30	0.23	0.24	0.24	0.24	0.23	0.24
1101	25	HB@	18	HA	0.65	0.34	0.57	0.57	0.47	0.41	0.51
1102	25	HB@	18	HB1	0.65	0.46	0.65	0.72	0.59	0.56	0.60
1103	25	HB@	18	HB2	0.65	0.32	0.53	0.57	0.42	0.40	0.45
1104	25	HD@	18	HB1	0.98	0.49	0.56	0.67	0.57	0.53	0.51
1105	25	HD@	18	HB2	0.98	0.33	0.53	0.55	0.43	0.39	0.37
1106	25	HD@	13	HB@	1.18	0.36	0.44	0.47	0.48	0.44	0.40
1107	25	HD@	17	HD@	1.51	0.78	0.64	0.65	0.62	0.67	0.78
1108	25	HD@	9	HA	0.98	0.36	0.38	0.40	0.43	0.42	0.39
1109	27	HB1	105	HE@	0.60	0.36	0.48	0.41	0.80	0.73	0.43
1110	27	HB2	105	HE@	0.60	0.49	0.37	0.49	0.90	0.85	0.58
1111	28	HA	31	HB@	0.60	0.35	0.35	0.33	0.33	0.33	0.34
1112	28	HA	105	HE@	0.45	0.32	0.41	0.50	0.71	0.74	0.49
1113	28	HA	28	HD1	0.45	0.44	0.45	0.44	0.44	0.43	0.44
1114	28	HB@	17	HD@	1.18	0.47	0.55	0.51	0.56	0.52	0.58
1115	29	HA	8	HD@	0.98	0.47	0.52	0.45	0.46	0.47	0.46
1116	29	HG2@	28	HD1	0.75	0.64	0.61	0.62	0.57	0.61	0.59
1117	29	HG2@	28	HB2	0.75	0.44	0.44	0.46	0.42	0.44	0.44
1118	29	HG2@	8	HD@	1.28	0.56	0.58	0.55	0.53	0.54	0.52
1119	29	HG1@	8	HD@	1.28	0.59	0.61	0.54	0.54	0.55	0.61
1120	29	HG1@	123	HA	0.75	0.50	0.59	0.61	0.66	0.68	0.57
1121	29	HG1@	30	HA	0.75	0.46	0.50	0.49	0.49	0.49	0.47
1122	29	HG1@	123	HD1	0.75	0.64	0.54	0.72	0.76	0.49	0.72
1123	31	HA	111	HB2	0.45	0.33	0.53	0.39	0.50	0.45	0.43

1124	31	HB@	111	HB1	0.75	0.46	0.60	0.52	0.45	0.48	0.56
1125	31	HB@	111	HB2	0.75	0.31	0.46	0.36	0.37	0.32	0.40
1126	31	HB@	105	HE@	0.90	0.32	0.38	0.50	0.62	0.65	0.49
1127	31	HB@	56	HD@	1.28	0.48	0.58	0.47	0.49	0.51	0.55
1128	33	HA	37	HA	0.45	0.30	0.29	0.34	0.29	0.28	0.28
1129	33	HA	33	HB1	0.30	0.29	0.29	0.27	0.28	0.29	0.29
1130	33	HA	33	HB2	0.30	0.23	0.24	0.26	0.25	0.24	0.24
1131	33	HA	33	HG@	0.65	0.34	0.28	0.29	0.28	0.28	0.29
1132	33	HB1	33	HG@	0.50	0.22	0.24	0.25	0.24	0.24	0.24
1133	33	HB2	33	HG@	0.50	0.25	0.25	0.24	0.25	0.25	0.25
1134	33	HG@	33	HD@	0.70	0.24	0.24	0.23	0.23	0.24	0.24
1135	33	HD@	33	HE@	0.70	0.24	0.23	0.24	0.23	0.23	0.23
1136	35	HA	35	HG@	0.65	0.31	0.26	0.27	0.27	0.28	0.29
1137	36	HB1	42	HB@	0.75	0.35	0.38	0.33	0.36	0.34	0.36
1138	36	HB2	42	HB@	0.75	0.28	0.32	0.38	0.34	0.32	0.30
1139	38	HB1	2	HG1@	0.75	0.43	0.43	0.36	0.39	0.40	0.38
1140	38	HB2	3	HB2	0.45	0.38	0.41	0.50	0.42	0.40	0.43
1141	38	HB2	55	HD@	0.75	0.55	0.73	0.70	0.71	0.66	0.61
1142	40	HA	55	HD@	0.75	0.43	0.50	0.42	0.48	0.47	0.44
1143	40	HA	55	HG2@	0.75	0.44	0.39	0.39	0.36	0.38	0.43
1144	40	HG2@	86	HA	0.60	0.35	0.34	0.42	0.34	0.36	0.36
1145	41	HA	41	HB1	0.30	0.25	0.25	0.25	0.25	0.26	0.25
1146	41	HA	41	HB2	0.45	0.29	0.28	0.28	0.28	0.28	0.29
1147	41	HA	84	HA	0.45	0.33	0.40	0.31	0.36	0.37	0.35
1148	41	HA	84	HD@	0.98	0.38	0.43	0.41	0.39	0.39	0.39
1149	41	HB1	84	HD@	0.98	0.51	0.59	0.51	0.53	0.55	0.52
1150	43	HA	53	HB1	0.45	0.53	0.58	0.52	0.55	0.56	0.55
1151	43	HG2@	53	HA	0.75	0.40	0.37	0.39	0.40	0.38	0.37
1152	43	HB	51	HG2@	0.60	0.32	0.36	0.34	0.32	0.33	0.33
1153	45	HA	51	HB	0.45	0.45	0.51	0.50	0.49	0.49	0.50

1154	51	HA	45	HB@	0.65	0.51	0.51	0.51	0.50	0.46	0.46
1155	51	HG2@	45	HB@	0.95	0.53	0.57	0.55	0.53	0.49	0.51
1156	55	HD@	38	HB1	0.75	0.42	0.58	0.62	0.58	0.53	0.48
1157	55	HD@	8	HD@	1.28	0.33	0.40	0.39	0.39	0.39	0.40
1158	55	HG2@	8	HD@	1.28	0.54	0.46	0.54	0.51	0.53	0.54
1159	55	HD@	56	HD@	1.28	0.74	0.60	0.64	0.58	0.60	0.65
1160	55	HG2@	91	HB@	0.95	0.35	0.54	0.43	0.48	0.45	0.38
1161	57	HA	57	HB@	0.65	0.25	0.25	0.26	0.26	0.26	0.26
1162	57	HA	57	HG@	0.65	0.31	0.30	0.25	0.28	0.27	0.27
1163	66	HA	80	HB2	0.45	0.35	0.35	0.30	0.36	0.34	0.34
1164	66	HB1	80	HB2	0.45	0.26	0.45	0.39	0.29	0.34	0.29
1165	66	HB2	80	HB2	0.45	0.36	0.36	0.34	0.37	0.40	0.38
1166	70	HA	70	HB@	0.50	0.25	0.24	0.24	0.24	0.24	0.24
1167	76	HB@	78	HA	0.65	0.57	0.58	0.58	0.58	0.58	0.58
1168	78	HA	79	HD@	0.50	0.23	0.22	0.22	0.22	0.22	0.22
1169	78	HB	79	HD@	0.50	0.30	0.34	0.37	0.36	0.36	0.34
1170	78	HG2@	64	HB1	0.75	0.63	0.71	0.44	0.62	0.60	0.63
1171	78	HG2@	64	HB2	0.75	0.62	0.68	0.46	0.59	0.58	0.62
1172	78	HG2@	79	HD@	0.95	0.41	0.44	0.45	0.42	0.43	0.43
1173	78	HG1@	79	HD@	0.85	0.50	0.53	0.47	0.51	0.49	0.51
1174	78	HD@	79	HD@	0.95	0.59	0.59	0.51	0.56	0.56	0.59
1175	81	HA	84	HB1	0.45	0.48	0.47	0.51	0.44	0.45	0.46
1176	81	HA	84	HB2	0.45	0.34	0.33	0.38	0.32	0.34	0.35
1177	86	HB@	40	HG2@	0.95	0.56	0.55	0.64	0.53	0.54	0.57
1178	86	HB@	1	HG@	0.85	0.56	0.51	0.50	0.54	0.52	0.52
1179	86	HB@	1	HD@	0.85	0.39	0.41	0.43	0.45	0.41	0.41
1180	92	HA	91	HA	0.45	0.46	0.46	0.47	0.47	0.47	0.47
1181	92	HG1@	93	HA	0.75	0.41	0.48	0.46	0.46	0.45	0.46
1182	92	HG1@	95	HB@	0.90	0.56	0.57	0.56	0.55	0.59	0.58
1183	92	HG2@	95	HB@	0.90	0.60	0.59	0.58	0.56	0.57	0.58

1184	93	HA	96	HD@	0.65	0.35	0.37	0.36	0.35	0.36	0.37
1185	93	HB1	90	HA	0.45	0.45	0.38	0.39	0.40	0.43	0.44
1186	93	HB2	90	HA	0.45	0.29	0.29	0.28	0.28	0.29	0.29
1187	94	HA	97	HB@	0.65	0.28	0.35	0.34	0.37	0.38	0.36
1188	96	HA	96	HG@	0.65	0.26	0.26	0.26	0.26	0.26	0.26
1189	96	HA	96	HD@	0.65	0.44	0.43	0.44	0.44	0.44	0.43
1190	96	HB@	96	HG@	0.65	0.23	0.24	0.24	0.24	0.24	0.24
1191	96	HG@	96	HE@	0.70	0.25	0.28	0.27	0.27	0.27	0.27
1192	96	HG@	96	HD@	0.70	0.24	0.23	0.23	0.23	0.23	0.23
1193	96	HD@	96	HE@	0.70	0.24	0.24	0.24	0.24	0.24	0.24
1194	96	HD@	17	HD@	1.18	0.63	0.62	0.61	0.61	0.62	0.56
1195	96	HE@	17	HD@	1.18	0.47	0.59	0.56	0.54	0.57	0.54
1196	98	HA	95	HA	0.45	0.54	0.54	0.56	0.53	0.54	0.53
1197	98	HA	95	HB@	0.75	0.76	0.76	0.78	0.75	0.75	0.76
1198	98	HB	95	HA	0.45	0.30	0.29	0.35	0.30	0.30	0.30
1199	98	HD@	95	HA	0.75	0.33	0.32	0.35	0.32	0.32	0.33
1200	98	HG2@	107	HB@	1.05	0.32	0.69	0.69	0.67	0.66	0.69
1201	98	HD@	58	HD@	1.05	0.40	0.42	0.52	0.41	0.45	0.42
1202	108	HB@	111	HB1	0.65	0.28	0.27	0.29	0.44	0.30	0.33
1203	108	HB@	111	HB2	0.65	0.29	0.30	0.34	0.49	0.38	0.38
1204	111	HA	115	HB@	0.65	0.34	0.28	0.31	0.42	0.33	0.32
1205	113	HB@	109	HG@	1.18	0.58	0.67	0.58	0.48	0.54	0.54
1206	116	HB@	116	HG@	0.65	0.22	0.23	0.24	0.23	0.23	0.24
1207	116	HG@	116	HD@	0.65	0.24	0.24	0.23	0.23	0.24	0.23
1208	118	HA	118	HG2@	0.60	0.30	0.28	0.29	0.28	0.31	0.29
1209	121	HA	124	HD@	0.75	0.47	0.39	0.41	0.42	0.40	0.44
1210	121	HA	124	HG1@	0.65	0.31	0.29	0.30	0.32	0.30	0.33
1211	123	HB@	29	HG1@	0.95	0.36	0.40	0.47	0.53	0.53	0.47
1212	123	HB@	29	HG2@	0.95	0.51	0.47	0.49	0.55	0.56	0.59
1213	124	HG2@	26	HA@	0.95	0.35	0.74	0.44	0.58	0.72	0.40

1214	124	HD@	26	HA@	0.95	0.52	0.81	0.60	0.73	0.80	0.54
1215	124	HD@	121	HG@	0.95	0.45	0.55	0.56	0.58	0.54	0.59
1216	2	HN	39	HA	0.55	0.48	0.47	0.49	0.47	0.48	0.49
1217	3	HN	8	HD@	1.08	0.60	0.64	0.62	0.62	0.63	0.63
1218	3	HN	38	HB2	0.45	0.33	0.34	0.40	0.36	0.35	0.35
1219	3	HN	39	HN	0.55	0.50	0.49	0.50	0.48	0.49	0.50
1220	3	HN	40	HB	0.55	0.69	0.75	0.71	0.72	0.72	0.73
1221	4	HN	38	HB2	0.55	0.59	0.58	0.66	0.58	0.58	0.61
1222	5	HN	38	HD@	0.89	0.55	0.54	0.62	0.55	0.55	0.57
1223	8	HN	3	HE@	0.89	0.60	0.68	0.64	0.65	0.63	0.65
1224	9	HN	124	HG2@	0.75	0.57	0.54	0.52	0.61	0.56	0.52
1225	11	HN	129	HD@	1.08	0.71	1.21	1.42	1.33	1.10	1.01
1226	12	HN	25	HD@	0.98	0.54	0.57	0.59	0.58	0.56	0.58
1227	14	HN	25	HD@	0.98	0.62	0.67	0.69	0.69	0.66	0.66
1228	17	HN	12	HB2	0.45	0.52	0.54	0.54	0.57	0.69	0.61
1229	17	HN	12	HA	0.75	0.42	0.43	0.42	0.45	0.57	0.44
1230	17	HN	25	HD@	1.08	0.59	0.61	0.60	0.55	0.67	0.70
1231	19	HN	25	HB@	0.65	0.48	0.69	0.67	0.59	0.52	0.61
1232	19	HN	28	HD1	0.75	0.38	0.74	0.70	0.68	0.62	0.58
1233	20	HN	28	HZ2	0.45	0.49	0.72	0.54	0.53	0.67	0.56
1234	24	HN	19	HA	0.45	0.41	0.52	0.52	0.52	0.50	0.45
1235	27	HN	120	HB	0.45	0.36	0.62	0.47	0.59	0.67	0.46
1236	28	HN	105	HE@	0.60	0.50	0.53	0.59	0.92	0.90	0.60
1237	31	HN	56	HD@	0.98	0.65	0.70	0.67	0.66	0.68	0.72
1238	31	HN	105	HE@	0.60	0.46	0.51	0.66	0.90	0.93	0.67
1239	31	HN	111	HB1	0.55	0.63	0.71	0.67	0.60	0.65	0.69
1240	31	HN	111	HB2	0.55	0.47	0.56	0.52	0.51	0.50	0.54
1241	39	HN	2	HA	0.55	0.46	0.46	0.47	0.45	0.46	0.46
1242	39	HN	2	HG1@	0.85	0.51	0.53	0.51	0.50	0.52	0.51
1243	39	HN	2	HG2@	0.85	0.57	0.54	0.59	0.54	0.55	0.55

1244	39	HN	55	HG2@	0.85	0.70	0.51	0.53	0.49	0.50	0.60
1245	39	HN	55	HD@	0.85	0.52	0.66	0.53	0.62	0.56	0.51
1246	40	HN	55	HD@	0.75	0.48	0.59	0.54	0.58	0.55	0.51
1247	42	HN	54	HN	0.75	0.41	0.47	0.40	0.40	0.42	0.41
1248	45	HN	51	HG2@	0.85	0.51	0.58	0.54	0.50	0.52	0.53
1249	46	HN	51	HG2@	0.85	0.51	0.55	0.54	0.54	0.54	0.56
1250	54	HN	42	HB@	0.85	0.44	0.56	0.44	0.42	0.46	0.45
1251	59	HN	98	HD@	0.85	0.52	0.59	0.79	0.56	0.62	0.56
1252	60	HN	51	HB	0.55	0.46	0.46	0.42	0.41	0.42	0.42
1253	61	HN	50	HB1	0.45	0.40	0.36	0.40	0.36	0.36	0.36
1254	61	HN	50	HB2	0.45	0.31	0.27	0.31	0.30	0.32	0.28
1255	63	HN	58	HG2@	0.75	0.51	0.48	0.55	0.55	0.53	0.50
1256	64	HN	58	HG2@	0.75	0.46	0.39	0.42	0.45	0.42	0.44
1257	64	HN	74	HA	0.55	0.39	0.52	0.44	0.47	0.45	0.47
1258	65	HN	78	HG1@	0.75	0.44	0.48	0.49	0.50	0.49	0.48
1259	65	HN	79	HA	0.45	0.28	0.31	0.31	0.30	0.30	0.30
1260	65	HN	74	HD@	0.75	0.40	0.67	0.45	0.63	0.54	0.61
1261	80	HN	66	HB@	0.65	0.42	0.49	0.49	0.44	0.48	0.45
1262	86	HN	40	HG2@	0.75	0.61	0.53	0.65	0.54	0.56	0.59
1263	88	HN	3	HE@	0.99	0.50	0.52	0.45	0.51	0.49	0.45
1264	88	HN	3	HZ	0.45	0.40	0.43	0.37	0.43	0.40	0.38
1265	91	HN	84	HD@	1.08	1.01	1.13	1.05	1.08	1.09	1.07
1266	92	HN	17	HD@	1.08	0.66	0.67	0.67	0.62	0.66	0.68
1267	95	HN	17	HD@	1.08	0.63	0.59	0.59	0.68	0.58	0.60
1268	98	HN	108	HZ2	0.55	0.55	0.81	0.80	0.97	0.77	0.85
1269	99	HN	20	HE@	0.99	0.60	0.70	0.76	0.70	0.69	0.70
1270	100	HN	20	HE@	0.99	0.50	0.53	0.71	0.56	0.58	0.54
1271	103	HN	98	HG2@	0.85	0.51	0.71	0.76	0.70	0.81	0.44
1272	104	HN	99	HA	0.45	0.53	0.40	0.56	0.69	0.60	0.50
1273	104	HN	99	HG@	0.98	0.64	0.68	0.82	0.97	0.84	0.75

1274	111	HN	31	HB@	0.75	0.49	0.51	0.55	0.44	0.46	0.55
1275	116	HN	111	HA	0.45	0.41	0.50	0.45	0.62	0.54	0.42
1276	127	HN	6	HB1	0.55	0.54	0.61	0.62	0.60	0.60	0.60
1277	128	HN	6	HB1	0.45	0.35	0.68	0.69	0.67	0.62	0.57
1278	128	HN	6	HB2	0.55	0.48	0.63	0.60	0.60	0.62	0.60
1279	129	HN	6	HB1	0.55	0.71	0.87	0.85	0.91	0.89	0.83
1280	2	HN	1	HB@	0.65	0.40	0.37	0.36	0.37	0.37	0.37
1281	2	HN	1	HG@	0.65	0.28	0.34	0.34	0.35	0.34	0.32
1282	3	HN	3	HE@	0.99	0.56	0.57	0.58	0.56	0.56	0.57
1283	3	HN	4	HN	0.45	0.47	0.45	0.44	0.45	0.45	0.45
1284	4	HN	3	HB1	0.45	0.29	0.26	0.30	0.26	0.26	0.27
1285	4	HN	3	HB2	0.45	0.41	0.38	0.41	0.38	0.39	0.39
1286	4	HN	5	HA	0.55	0.55	0.54	0.55	0.54	0.54	0.55
1287	4	HN	7	HG@	0.65	0.44	0.42	0.41	0.41	0.39	0.40
1288	4	HN	7	HN	0.45	0.42	0.46	0.42	0.43	0.43	0.43
1289	4	HN	3	HA	0.45	0.23	0.23	0.22	0.24	0.23	0.23
1290	5	HN	5	HB@	0.50	0.30	0.27	0.27	0.24	0.24	0.27
1291	5	HN	5	HG@	0.65	0.28	0.29	0.29	0.36	0.35	0.29
1292	6	HN	4	HA@	0.75	0.41	0.43	0.43	0.43	0.43	0.43
1293	6	HN	5	HB@	0.50	0.38	0.30	0.29	0.31	0.31	0.29
1294	6	HN	5	HG@	0.65	0.25	0.36	0.38	0.36	0.37	0.39
1295	6	HN	9	HN	0.55	0.46	0.48	0.48	0.48	0.48	0.48
1296	7	HN	3	HD@	0.99	0.64	0.75	0.68	0.70	0.69	0.69
1297	7	HN	4	HA@	0.65	0.41	0.43	0.43	0.44	0.44	0.43
1298	7	HN	6	HA	0.45	0.35	0.35	0.35	0.35	0.35	0.35
1299	7	HN	7	HG@	0.65	0.34	0.33	0.31	0.31	0.31	0.31
1300	8	HN	5	HA	0.55	0.36	0.38	0.38	0.39	0.39	0.39
1301	8	HN	7	HA	0.45	0.35	0.35	0.35	0.35	0.35	0.35
1302	8	HN	7	HG@	0.65	0.49	0.41	0.45	0.42	0.41	0.43
1303	9	HN	6	HB1	0.45	0.51	0.55	0.54	0.55	0.56	0.54

1304	9	HN	6	HB2	0.55	0.54	0.57	0.57	0.57	0.57	0.56
1305	9	HN	7	HA	0.45	0.45	0.47	0.46	0.47	0.47	0.47
1306	9	HN	12	HN	0.45	0.48	0.48	0.48	0.48	0.48	0.48
1307	9	HN	5	HA	0.45	0.39	0.41	0.42	0.40	0.39	0.41
1308	10	HN	8	HA	0.55	0.43	0.47	0.46	0.46	0.46	0.46
1309	11	HN	9	HA	0.45	0.46	0.47	0.46	0.46	0.46	0.46
1310	11	HN	12	HG@	0.75	0.55	0.55	0.56	0.56	0.57	0.59
1311	11	HN	8	HD@	0.98	0.63	0.69	0.69	0.68	0.68	0.67
1312	12	HN	8	HA	0.55	0.38	0.39	0.39	0.39	0.38	0.39
1313	12	HN	12	HG@	0.50	0.30	0.28	0.29	0.29	0.30	0.31
1314	13	HN	9	HA	0.45	0.41	0.40	0.40	0.40	0.39	0.39
1315	13	HN	11	HA	0.45	0.43	0.46	0.46	0.46	0.46	0.46
1316	13	HN	12	HG@	0.65	0.47	0.46	0.46	0.47	0.46	0.47
1317	13	HN	15	HN	0.45	0.40	0.44	0.43	0.44	0.43	0.43
1318	13	HN	16	HN	0.55	0.51	0.52	0.52	0.52	0.49	0.51
1319	14	HN	12	HA	0.55	0.45	0.46	0.46	0.46	0.46	0.46
1320	14	HN	13	HG@	0.65	0.42	0.42	0.44	0.43	0.40	0.39
1321	14	HN	16	HN	0.45	0.41	0.43	0.43	0.43	0.44	0.44
1322	14	HN	15	HB2	0.45	0.51	0.50	0.51	0.50	0.50	0.51
1323	15	HN	12	HA	0.45	0.34	0.35	0.35	0.35	0.35	0.36
1324	15	HN	13	HA	0.45	0.41	0.45	0.43	0.44	0.42	0.40
1325	15	HN	14	HA	0.45	0.34	0.35	0.34	0.35	0.34	0.34
1326	15	HN	14	HB@	0.65	0.33	0.31	0.32	0.31	0.32	0.33
1327	15	HN	17	HD@	1.28	0.70	0.80	0.78	0.71	0.83	0.72
1328	16	HN	15	HA	0.45	0.31	0.30	0.30	0.30	0.33	0.30
1329	16	HN	18	HN	0.45	0.41	0.53	0.58	0.55	0.47	0.48
1330	16	HN	17	HG	0.45	0.49	0.62	0.64	0.56	0.52	0.54
1331	16	HN	15	HB1	0.45	0.44	0.41	0.42	0.41	0.38	0.41
1332	16	HN	15	HB2	0.45	0.39	0.38	0.38	0.37	0.33	0.35
1333	17	HN	16	HA@	0.65	0.32	0.30	0.30	0.29	0.25	0.27

1334	17	HN	18	HB1	0.45	0.51	0.54	0.57	0.50	0.53	0.51
1335	17	HN	18	HB2	0.55	0.50	0.53	0.52	0.48	0.53	0.51
1336	18	HN	19	HN	0.45	0.45	0.44	0.44	0.44	0.44	0.45
1337	18	HN	20	HN	0.55	0.43	0.54	0.49	0.51	0.47	0.45
1338	20	HN	17	HD@	0.98	0.62	0.74	0.66	0.73	0.67	0.68
1339	20	HN	19	HA	0.30	0.30	0.23	0.24	0.25	0.28	0.30
1340	20	HN	19	HB@	0.65	0.42	0.39	0.41	0.38	0.40	0.40
1341	20	HN	23	HA	0.45	0.57	0.54	0.58	0.56	0.59	0.60
1342	21	HN	20	HB1	0.45	0.42	0.32	0.31	0.40	0.41	0.41
1343	21	HN	20	HB2	0.45	0.42	0.38	0.39	0.42	0.42	0.42
1344	21	HN	21	HB@	0.65	0.36	0.32	0.33	0.34	0.35	0.35
1345	21	HN	21	HG@	0.75	0.35	0.31	0.32	0.32	0.32	0.32
1346	22	HN	19	HA	0.55	0.37	0.51	0.51	0.47	0.46	0.43
1347	22	HN	20	HA	0.45	0.36	0.42	0.41	0.39	0.39	0.39
1348	22	HN	20	HN	0.55	0.44	0.47	0.49	0.45	0.48	0.47
1349	22	HN	21	HB@	0.65	0.42	0.42	0.41	0.42	0.43	0.42
1350	23	HN	19	HA	0.45	0.32	0.47	0.46	0.44	0.43	0.42
1351	23	HN	20	HN	0.55	0.37	0.38	0.41	0.38	0.43	0.42
1352	23	HN	22	HA@	0.65	0.32	0.31	0.31	0.31	0.31	0.32
1353	23	HN	21	HB@	0.75	0.45	0.44	0.44	0.43	0.43	0.43
1354	24	HN	23	HB1	0.45	0.36	0.28	0.30	0.29	0.30	0.34
1355	24	HN	23	HB2	0.45	0.43	0.39	0.40	0.39	0.40	0.42
1356	24	HN	24	HB1	0.30	0.25	0.30	0.27	0.27	0.27	0.28
1357	24	HN	24	HB2	0.45	0.35	0.32	0.29	0.27	0.27	0.28
1358	24	HN	25	HN	0.45	0.46	0.42	0.45	0.45	0.45	0.45
1359	24	HN	27	HB1	0.55	0.48	0.45	0.46	0.49	0.48	0.40
1360	24	HN	27	HB2	0.55	0.35	0.50	0.34	0.36	0.36	0.28
1361	24	HN	27	HD@	0.65	0.27	0.39	0.41	0.49	0.48	0.40
1362	24	HN	27	HN	0.55	0.41	0.38	0.38	0.41	0.41	0.40
1363	25	HN	24	HA	0.30	0.23	0.26	0.25	0.24	0.24	0.25

1364	26	HN	27	HD@	0.75	0.55	0.63	0.62	0.70	0.70	0.60
1365	26	HN	28	HD1	0.55	0.50	0.51	0.52	0.51	0.52	0.51
1366	27	HN	25	HA	0.45	0.43	0.45	0.42	0.40	0.41	0.42
1367	27	HN	26	HA@	0.65	0.31	0.30	0.31	0.31	0.31	0.31
1368	27	HN	27	HD@	0.65	0.34	0.42	0.39	0.44	0.44	0.37
1369	27	HN	29	HN	0.45	0.44	0.45	0.43	0.43	0.44	0.43
1370	27	HN	28	HD1	0.45	0.45	0.49	0.45	0.44	0.44	0.44
1371	28	HN	25	HA	0.45	0.34	0.38	0.36	0.37	0.35	0.37
1372	28	HN	27	HB1	0.45	0.34	0.39	0.33	0.32	0.32	0.31
1373	28	HN	29	HB	0.55	0.49	0.52	0.51	0.50	0.49	0.49
1374	28	HN	29	HG2@	0.75	0.54	0.54	0.54	0.52	0.53	0.54
1375	28	HN	30	HN	0.55	0.42	0.43	0.44	0.43	0.43	0.44
1376	29	HN	28	HA	0.45	0.35	0.35	0.35	0.35	0.35	0.35
1377	29	HN	30	HB1	0.55	0.47	0.48	0.49	0.46	0.46	0.48
1378	29	HN	30	HB2	0.55	0.49	0.50	0.51	0.52	0.54	0.52
1379	29	HN	25	HA	0.55	0.42	0.42	0.43	0.46	0.43	0.44
1380	29	HN	28	HD1	0.55	0.47	0.45	0.45	0.44	0.45	0.43
1381	30	HN	31	HN	0.30	0.28	0.30	0.30	0.30	0.29	0.29
1382	31	HN	27	HA	0.45	0.41	0.39	0.40	0.40	0.41	0.40
1383	31	HN	29	HA	0.45	0.43	0.46	0.46	0.46	0.45	0.46
1384	31	HN	30	HA	0.45	0.35	0.35	0.35	0.35	0.35	0.35
1385	31	HN	33	HN	0.55	0.42	0.44	0.45	0.45	0.44	0.44
1386	32	HN	30	HA	0.55	0.44	0.46	0.46	0.44	0.45	0.46
1387	32	HN	29	HA	0.45	0.34	0.35	0.35	0.35	0.35	0.36
1388	32	HN	31	HB@	0.55	0.33	0.32	0.32	0.33	0.32	0.32
1389	33	HN	31	HA	0.55	0.44	0.46	0.46	0.40	0.44	0.45
1390	33	HN	29	HA	0.45	0.41	0.40	0.40	0.47	0.43	0.41
1391	33	HN	33	HB1	0.30	0.24	0.25	0.28	0.26	0.25	0.25
1392	33	HN	33	HB2	0.30	0.26	0.23	0.23	0.23	0.23	0.23
1393	33	HN	33	HD@	0.75	0.49	0.47	0.46	0.47	0.48	0.47

1394	33	HN	33	HG@	0.75	0.44	0.41	0.35	0.37	0.42	0.42
1395	33	HN	35	HN	0.45	0.42	0.45	0.44	0.61	0.44	0.44
1396	34	HN	32	HN	0.55	0.44	0.46	0.45	0.47	0.46	0.46
1397	34	HN	33	HB1	0.45	0.26	0.24	0.28	0.27	0.24	0.25
1398	34	HN	33	HB2	0.45	0.39	0.36	0.33	0.35	0.37	0.37
1399	34	HN	33	HG@	0.65	0.38	0.40	0.43	0.42	0.39	0.41
1400	35	HN	32	HA	0.45	0.34	0.38	0.36	0.56	0.37	0.36
1401	35	HN	33	HA	0.55	0.46	0.47	0.46	0.57	0.47	0.47
1402	35	HN	34	HA	0.45	0.34	0.34	0.34	0.23	0.35	0.34
1403	35	HN	35	HG@	0.50	0.32	0.32	0.29	0.34	0.29	0.30
1404	35	HN	37	HN	0.45	0.39	0.39	0.52	0.42	0.42	0.41
1405	36	HN	32	HA	0.45	0.36	0.31	0.36	0.44	0.33	0.32
1406	36	HN	32	HB@	0.85	0.53	0.52	0.55	0.65	0.53	0.51
1407	36	HN	35	HB@	0.65	0.31	0.28	0.26	0.29	0.27	0.28
1408	36	HN	34	HA	0.45	0.42	0.52	0.49	0.45	0.51	0.49
1409	38	HN	38	HD@	0.89	0.35	0.33	0.34	0.33	0.34	0.34
1410	39	HN	38	HA	0.45	0.30	0.29	0.31	0.30	0.29	0.29
1411	40	HN	39	HB1	0.45	0.43	0.40	0.40	0.41	0.41	0.41
1412	40	HN	39	HB2	0.45	0.44	0.41	0.41	0.41	0.41	0.41
1413	40	HN	39	HD@	0.75	0.55	0.50	0.51	0.52	0.52	0.52
1414	40	HN	41	HA	0.55	0.53	0.52	0.52	0.52	0.52	0.52
1415	40	HN	41	HB1	0.55	0.62	0.60	0.60	0.60	0.60	0.61
1416	40	HN	41	HB2	0.45	0.49	0.48	0.48	0.49	0.48	0.49
1417	41	HN	40	HB	0.45	0.40	0.35	0.38	0.36	0.36	0.38
1418	41	HN	40	HG2@	0.75	0.50	0.49	0.50	0.49	0.49	0.50
1419	41	HN	41	HB@	0.50	0.29	0.28	0.28	0.28	0.28	0.28
1420	42	HN	39	HD@	0.75	0.51	0.45	0.48	0.45	0.44	0.44
1421	42	HN	40	HG2@	0.85	0.64	0.65	0.65	0.64	0.64	0.65
1422	42	HN	41	HA	0.45	0.32	0.31	0.29	0.29	0.30	0.30
1423	42	HN	41	HB@	0.65	0.40	0.37	0.40	0.40	0.39	0.40

1424	42	HN	43	HN	0.45	0.46	0.44	0.44	0.43	0.43	0.44
1425	43	HN	44	HN	0.55	0.44	0.40	0.41	0.38	0.39	0.42
1426	44	HN	43	HB	0.45	0.29	0.34	0.32	0.35	0.34	0.31
1427	44	HN	43	HG2@	0.75	0.45	0.46	0.45	0.47	0.46	0.45
1428	46	HN	45	HB@	0.65	0.39	0.39	0.37	0.37	0.35	0.35
1429	46	HN	46	HD@	0.65	0.33	0.46	0.37	0.34	0.33	0.33
1430	46	HN	50	HN	0.55	0.44	0.43	0.39	0.43	0.44	0.39
1431	47	HN	46	HB@	0.50	0.31	0.33	0.32	0.30	0.30	0.30
1432	48	HN	47	HA	0.45	0.33	0.34	0.34	0.34	0.34	0.34
1433	48	HN	47	HG2@	0.75	0.50	0.49	0.50	0.50	0.50	0.50
1434	48	HN	50	HN	0.45	0.37	0.39	0.38	0.40	0.41	0.38
1435	49	HN	47	HA	0.45	0.32	0.38	0.36	0.37	0.38	0.35
1436	49	HN	48	HA	0.45	0.32	0.33	0.32	0.33	0.32	0.32
1437	49	HN	48	HB1	0.55	0.44	0.41	0.43	0.42	0.41	0.43
1438	49	HN	48	HB2	0.55	0.44	0.39	0.41	0.40	0.35	0.41
1439	50	HN	49	HA@	0.65	0.31	0.31	0.31	0.31	0.31	0.31
1440	51	HN	52	HA	0.55	0.47	0.52	0.50	0.50	0.50	0.50
1441	51	HN	52	HN	0.45	0.43	0.44	0.44	0.43	0.43	0.44
1442	52	HN	53	HD@	0.99	0.55	0.58	0.57	0.59	0.59	0.57
1443	52	HN	53	HE@	0.99	0.57	0.67	0.64	0.68	0.68	0.65
1444	53	HN	51	HG2@	0.85	0.56	0.63	0.58	0.56	0.58	0.57
1445	54	HN	52	HA	0.45	0.61	0.62	0.61	0.60	0.61	0.61
1446	54	HN	57	HN	0.55	0.43	0.49	0.60	0.53	0.49	0.48
1447	54	HN	53	HB2	0.45	0.42	0.39	0.41	0.42	0.41	0.42
1448	54	HN	53	HE@	0.99	0.69	0.64	0.67	0.64	0.64	0.65
1449	54	HN	53	HD@	0.99	0.45	0.41	0.44	0.42	0.41	0.43
1450	56	HN	55	HA	0.55	0.34	0.35	0.34	0.35	0.35	0.34
1451	56	HN	55	HB	0.55	0.41	0.30	0.37	0.35	0.36	0.37
1452	56	HN	57	HA	0.45	0.45	0.46	0.47	0.47	0.45	0.44
1453	56	HN	58	HN	0.55	0.41	0.45	0.44	0.43	0.44	0.42

1454	57	HN	57	HG@	0.65	0.34	0.32	0.31	0.32	0.32	0.31
1455	60	HN	61	HB@	0.75	0.50	0.53	0.55	0.52	0.52	0.52
1456	62	HN	61	HB@	0.65	0.30	0.31	0.33	0.33	0.33	0.33
1457	62	HN	62	HB@	0.65	0.29	0.27	0.31	0.28	0.25	0.28
1458	62	HN	63	HN	0.45	0.27	0.30	0.32	0.28	0.28	0.29
1459	63	HN	62	HA	0.45	0.35	0.35	0.35	0.35	0.35	0.35
1460	63	HN	62	HB@	0.65	0.35	0.26	0.28	0.28	0.29	0.27
1461	63	HN	63	HB@	0.65	0.31	0.30	0.29	0.30	0.30	0.30
1462	63	HN	64	HN	0.45	0.24	0.27	0.28	0.29	0.27	0.27
1463	64	HN	65	HN	0.55	0.45	0.46	0.45	0.46	0.46	0.45
1464	67	HN	68	HN	0.30	0.27	0.28	0.28	0.28	0.29	0.28
1465	67	HN	69	HN	0.45	0.38	0.43	0.43	0.44	0.43	0.40
1466	68	HN	67	HA@	0.65	0.31	0.31	0.31	0.31	0.31	0.31
1467	68	HN	69	HN	0.25	0.20	0.25	0.23	0.24	0.23	0.22
1468	69	HN	68	HB@	0.65	0.40	0.31	0.33	0.30	0.32	0.34
1469	69	HN	70	HD@	0.75	0.49	0.47	0.44	0.46	0.47	0.46
1470	69	HN	69	HA	0.45	0.29	0.27	0.28	0.27	0.27	0.28
1471	71	HN	69	HG2@	0.85	0.62	0.70	0.72	0.65	0.66	0.66
1472	71	HN	70	HB@	0.65	0.34	0.32	0.30	0.32	0.32	0.31
1473	72	HN	69	HG2@	0.85	0.56	0.62	0.72	0.61	0.60	0.61
1474	72	HN	70	HB@	0.75	0.55	0.56	0.56	0.56	0.55	0.54
1475	72	HN	73	HN	0.45	0.46	0.45	0.44	0.44	0.45	0.45
1476	73	HN	73	HA	0.45	0.29	0.27	0.27	0.28	0.28	0.27
1477	73	HN	73	HB@	0.65	0.30	0.28	0.27	0.28	0.29	0.29
1478	73	HN	74	HN	0.45	0.21	0.26	0.27	0.25	0.25	0.25
1479	74	HN	73	HA	0.45	0.34	0.34	0.33	0.34	0.34	0.34
1480	74	HN	75	HD@	1.08	0.60	0.62	0.65	0.62	0.61	0.63
1481	74	HN	75	HG	0.45	0.43	0.46	0.51	0.45	0.44	0.45
1482	75	HN	76	HA	0.55	0.51	0.53	0.53	0.53	0.53	0.53
1483	75	HN	77	HN	0.45	0.41	0.43	0.44	0.43	0.44	0.43

1484	77	HN	77	HA	0.25	0.22	0.20	0.21	0.20	0.20	0.20
1485	77	HN	78	HD@	0.75	0.65	0.68	0.66	0.68	0.68	0.68
1486	77	HN	78	HG2@	0.85	0.61	0.66	0.58	0.67	0.65	0.64
1487	78	HN	79	HD@	0.65	0.49	0.48	0.46	0.48	0.48	0.48
1488	80	HN	79	HB@	0.65	0.35	0.34	0.35	0.34	0.34	0.34
1489	80	HN	80	HB1	0.45	0.35	0.35	0.35	0.35	0.35	0.35
1490	80	HN	80	HB2	0.45	0.25	0.24	0.24	0.24	0.24	0.24
1491	80	HN	82	HN	0.55	0.44	0.45	0.45	0.45	0.45	0.45
1492	81	HN	79	HA	0.45	0.39	0.42	0.42	0.42	0.42	0.42
1493	81	HN	79	HB@	0.65	0.34	0.35	0.37	0.35	0.36	0.36
1494	81	HN	80	HB1	0.45	0.39	0.36	0.36	0.36	0.36	0.36
1495	81	HN	80	HB2	0.45	0.30	0.28	0.28	0.28	0.28	0.28
1496	81	HN	84	HD@	0.98	0.68	0.66	0.69	0.66	0.67	0.68
1497	82	HN	81	HA	0.45	0.35	0.35	0.34	0.35	0.35	0.35
1498	82	HN	79	HB@	0.65	0.41	0.34	0.36	0.34	0.33	0.35
1499	82	HN	83	HD@	0.98	0.61	0.64	0.62	0.65	0.63	0.62
1500	82	HN	84	HN	0.45	0.42	0.44	0.44	0.44	0.45	0.44
1501	83	HN	85	HN	0.45	0.43	0.45	0.50	0.45	0.45	0.45
1502	84	HN	85	HN	0.30	0.23	0.29	0.30	0.30	0.29	0.27
1503	85	HN	84	HB@	0.65	0.40	0.36	0.40	0.36	0.36	0.37
1504	85	HN	84	HD@	0.98	0.59	0.57	0.58	0.57	0.57	0.57
1505	85	HN	84	HG	0.45	0.48	0.48	0.49	0.47	0.47	0.47
1506	85	HN	86	HN	0.55	0.47	0.45	0.45	0.44	0.45	0.45
1507	86	HN	87	HA	0.55	0.52	0.53	0.53	0.54	0.54	0.54
1508	86	HN	87	HB1	0.45	0.46	0.51	0.52	0.51	0.51	0.51
1509	86	HN	87	HB2	0.45	0.51	0.51	0.53	0.53	0.54	0.54
1510	88	HN	87	HB1	0.55	0.41	0.39	0.36	0.39	0.39	0.39
1511	88	HN	88	HB	0.45	0.37	0.27	0.35	0.30	0.28	0.31
1512	88	HN	89	HN	0.45	0.27	0.26	0.31	0.27	0.27	0.28
1513	89	HN	88	HA	0.45	0.31	0.32	0.27	0.31	0.30	0.30

1514	89	HN	87	HA	0.45	0.41	0.40	0.44	0.40	0.40	0.41
1515	89	HN	90	HB@	0.75	0.59	0.55	0.55	0.55	0.55	0.55
1516	89	HN	91	HN	0.55	0.45	0.44	0.43	0.45	0.45	0.45
1517	90	HN	87	HA	0.55	0.66	0.60	0.70	0.59	0.61	0.63
1518	91	HN	93	HN	0.45	0.43	0.45	0.45	0.45	0.46	0.46
1519	91	HN	89	HA	0.75	0.46	0.46	0.46	0.46	0.46	0.46
1520	92	HN	88	HD@	0.85	0.55	0.55	0.54	0.54	0.53	0.56
1521	92	HN	93	HA	0.55	0.52	0.53	0.53	0.53	0.53	0.52
1522	93	HN	92	HA	0.45	0.35	0.35	0.35	0.35	0.35	0.35
1523	94	HN	93	HA	0.45	0.35	0.35	0.35	0.35	0.35	0.35
1524	94	HN	95	HB@	0.75	0.53	0.54	0.54	0.54	0.54	0.54
1525	94	HN	92	HA	0.55	0.43	0.46	0.46	0.45	0.46	0.45
1526	95	HN	93	HA	0.45	0.45	0.46	0.45	0.45	0.45	0.45
1527	96	HN	95	HA	0.45	0.35	0.35	0.35	0.35	0.35	0.35
1528	96	HN	92	HA	0.55	0.40	0.40	0.41	0.40	0.42	0.41
1529	96	HN	94	HN	0.45	0.41	0.45	0.45	0.45	0.45	0.45
1530	96	HN	96	HD@	0.65	0.44	0.43	0.43	0.42	0.42	0.43
1531	96	HN	96	HG@	0.75	0.30	0.28	0.28	0.28	0.28	0.29
1532	96	HN	94	HA	0.55	0.42	0.44	0.44	0.44	0.43	0.43
1533	97	HN	96	HG@	0.75	0.47	0.46	0.44	0.47	0.47	0.45
1534	97	HN	97	HB@	0.50	0.23	0.28	0.26	0.28	0.28	0.27
1535	97	HN	93	HA	0.55	0.43	0.45	0.45	0.48	0.49	0.47
1536	97	HN	96	HA	0.45	0.35	0.35	0.35	0.35	0.35	0.35
1537	98	HN	99	HB	0.55	0.50	0.64	0.59	0.57	0.55	0.60
1538	98	HN	99	HG@	1.08	0.62	0.55	0.54	0.57	0.58	0.55
1539	98	HN	100	HN	0.55	0.42	0.43	0.47	0.44	0.45	0.44
1540	98	HN	96	HA	0.55	0.45	0.45	0.46	0.44	0.44	0.46
1541	99	HN	98	HA	0.45	0.35	0.35	0.34	0.33	0.35	0.35
1542	99	HN	97	HA	0.45	0.43	0.46	0.44	0.45	0.45	0.46
1543	100	HN	97	HN	0.55	0.47	0.49	0.56	0.51	0.53	0.50

1544	100	HN	99	HG@	0.98	0.41	0.41	0.40	0.40	0.41	0.40
1545	101	HN	100	HA	0.45	0.34	0.34	0.33	0.32	0.30	0.33
1546	102	HN	100	HA	0.55	0.33	0.63	0.63	0.61	0.56	0.54
1547	102	HN	101	HB@	0.65	0.40	0.37	0.36	0.37	0.37	0.32
1548	102	HN	103	HA	0.55	0.49	0.53	0.52	0.53	0.53	0.51
1549	103	HN	99	HG@	0.98	0.57	0.93	0.94	1.05	0.94	0.82
1550	103	HN	102	HA@	0.65	0.31	0.31	0.31	0.30	0.30	0.26
1551	103	HN	103	HD@	0.75	0.26	0.33	0.42	0.38	0.37	0.46
1552	104	HN	103	HA	0.30	0.23	0.34	0.28	0.29	0.30	0.23
1553	104	HN	106	HN	0.55	0.40	0.48	0.41	0.45	0.41	0.41
1554	105	HN	105	HG@	0.75	0.28	0.32	0.32	0.32	0.30	0.36
1555	105	HN	106	HB1	0.55	0.61	0.50	0.56	0.59	0.63	0.63
1556	105	HN	106	HB2	0.55	0.46	0.57	0.50	0.51	0.51	0.52
1557	105	HN	106	HN	0.30	0.28	0.31	0.28	0.30	0.31	0.31
1558	105	HN	107	HN	0.45	0.45	0.57	0.48	0.45	0.47	0.45
1559	105	HN	105	HE@	0.60	0.59	0.60	0.47	0.49	0.50	0.53
1560	107	HN	106	HB1	0.45	0.34	0.37	0.33	0.32	0.34	0.34
1561	107	HN	106	HB2	0.45	0.32	0.35	0.32	0.31	0.29	0.28
1562	107	HN	108	HE3	0.45	0.37	0.34	0.45	0.64	0.40	0.54
1563	107	HN	108	HZ3	0.45	0.46	0.43	0.56	0.84	0.52	0.66
1564	108	HN	106	HA	0.45	0.37	0.39	0.36	0.44	0.43	0.37
1565	108	HN	109	HN	0.45	0.43	0.44	0.42	0.43	0.39	0.43
1566	109	HN	108	HA	0.45	0.22	0.21	0.21	0.22	0.22	0.21
1567	109	HN	108	HB@	0.65	0.39	0.39	0.38	0.39	0.41	0.36
1568	109	HN	110	HB@	0.75	0.56	0.54	0.54	0.50	0.54	0.54
1569	110	HN	108	HA	0.45	0.39	0.40	0.41	0.39	0.38	0.41
1570	110	HN	108	HB@	0.65	0.39	0.42	0.41	0.45	0.48	0.39
1571	110	HN	112	HN	0.45	0.43	0.46	0.46	0.46	0.45	0.46
1572	111	HN	108	HA	0.55	0.43	0.44	0.45	0.53	0.43	0.47
1573	111	HN	112	HB@	0.65	0.57	0.53	0.51	0.55	0.53	0.51

1574	111	HN	110	HA	0.45	0.35	0.35	0.34	0.35	0.35	0.35
1575	111	HN	110	HB@	0.75	0.36	0.34	0.34	0.33	0.32	0.33
1576	112	HN	111	HA	0.45	0.36	0.35	0.35	0.34	0.35	0.35
1577	112	HN	110	HA	0.45	0.44	0.46	0.47	0.44	0.46	0.48
1578	112	HN	112	HG@	0.65	0.27	0.27	0.30	0.29	0.33	0.30
1579	113	HN	110	HA	0.45	0.32	0.38	0.39	0.38	0.40	0.41
1580	113	HN	112	HA	0.45	0.37	0.35	0.35	0.34	0.35	0.35
1581	113	HN	114	HB1	0.55	0.57	0.58	0.62	0.65	0.59	0.61
1582	113	HN	114	HB2	0.55	0.49	0.50	0.50	0.53	0.49	0.49
1583	113	HN	115	HN	0.45	0.41	0.44	0.45	0.44	0.43	0.45
1584	113	HN	109	HA	0.55	0.44	0.41	0.36	0.38	0.34	0.34
1585	114	HN	111	HA	0.55	0.37	0.40	0.40	0.62	0.47	0.40
1586	114	HN	116	HN	0.45	0.38	0.54	0.44	0.54	0.55	0.43
1587	115	HN	114	HA	0.45	0.35	0.33	0.33	0.32	0.34	0.34
1588	115	HN	116	HB1	0.55	0.38	0.66	0.54	0.63	0.65	0.52
1589	115	HN	116	HB2	0.55	0.43	0.57	0.46	0.57	0.57	0.46
1590	115	HN	116	HN	0.30	0.20	0.37	0.26	0.36	0.37	0.25
1591	115	HN	111	HA	0.55	0.34	0.28	0.31	0.45	0.36	0.31
1592	116	HN	115	HB@	0.65	0.29	0.37	0.36	0.36	0.33	0.33
1593	116	HN	116	HG@	0.65	0.42	0.32	0.33	0.31	0.35	0.32
1594	116	HN	113	HN	0.55	0.49	0.70	0.58	0.66	0.69	0.54
1595	117	HN	116	HG@	0.65	0.34	0.44	0.41	0.42	0.39	0.39
1596	118	HN	116	HA	0.45	0.36	0.49	0.40	0.41	0.45	0.38
1597	118	HN	117	HA@	0.65	0.32	0.28	0.30	0.29	0.29	0.30
1598	119	HN	120	HN	0.45	0.39	0.44	0.44	0.45	0.45	0.44
1599	120	HN	119	HB1	0.45	0.44	0.35	0.40	0.41	0.39	0.41
1600	120	HN	119	HB2	0.45	0.46	0.40	0.40	0.39	0.39	0.40
1601	120	HN	120	HB	0.45	0.36	0.25	0.27	0.26	0.26	0.30
1602	120	HN	122	HN	0.45	0.44	0.49	0.46	0.46	0.46	0.45
1603	120	HN	123	HN	0.45	0.57	0.55	0.50	0.49	0.50	0.50

1604	121	HN	121	HG@	0.65	0.44	0.31	0.31	0.31	0.31	0.32
1605	122	HN	119	HA	0.55	0.46	0.55	0.48	0.49	0.50	0.48
1606	122	HN	121	HB@	0.65	0.35	0.30	0.30	0.30	0.30	0.30
1607	122	HN	121	HG@	0.65	0.50	0.46	0.46	0.45	0.46	0.45
1608	123	HN	124	HD@	0.85	0.70	0.66	0.68	0.70	0.68	0.69
1609	123	HN	124	HG2@	0.85	0.55	0.58	0.59	0.58	0.58	0.59
1610	123	HN	125	HB@	0.75	0.52	0.62	0.61	0.61	0.62	0.62
1611	124	HN	121	HA	0.45	0.34	0.37	0.36	0.36	0.36	0.37
1612	124	HN	122	HA	0.45	0.40	0.47	0.43	0.46	0.47	0.44
1613	124	HN	123	HA	0.45	0.34	0.35	0.34	0.35	0.35	0.34
1614	124	HN	125	HB@	0.65	0.42	0.55	0.51	0.54	0.54	0.50
1615	125	HN	122	HA	0.45	0.33	0.41	0.36	0.37	0.39	0.38
1616	125	HN	124	HA	0.45	0.31	0.29	0.33	0.34	0.31	0.30
1617	125	HN	124	HD@	0.75	0.57	0.52	0.47	0.50	0.51	0.49
1618	125	HN	124	HB	0.45	0.44	0.40	0.39	0.38	0.39	0.40
1619	125	HN	123	HB1	0.55	0.55	0.55	0.56	0.55	0.53	0.55
1620	125	HN	123	HB2	0.55	0.52	0.52	0.55	0.53	0.53	0.54
1621	126	HN	127	HB1	0.55	0.62	0.63	0.65	0.64	0.62	0.63
1622	126	HN	127	HB2	0.55	0.50	0.53	0.58	0.57	0.55	0.54
1623	127	HN	124	HA	0.45	0.44	0.59	0.64	0.62	0.59	0.57
1624	127	HN	124	HB	0.55	0.45	0.63	0.73	0.77	0.67	0.60
1625	127	HN	126	HA@	0.65	0.32	0.28	0.27	0.28	0.28	0.28
1626	127	HN	129	HD@	0.98	0.57	0.83	0.75	0.77	0.83	0.75
1627	128	HN	129	HD@	1.08	0.59	0.69	0.63	0.66	0.67	0.65
1628	129	HN	127	HB1	0.55	0.52	0.56	0.55	0.57	0.57	0.54
1629	129	HN	127	HB2	0.55	0.51	0.57	0.57	0.58	0.59	0.56
1630	129	HN	128	HB@	0.65	0.40	0.33	0.34	0.35	0.34	0.34

Table S2: S^2_{CH} values (51), S^2_{NH} values (11) for Trp (NE1-HE1) and Arg (NE-HE) side chains, and S^2_{NH} values (17) for Asn (ND2-HD21, -HD22) and Gln (NE2-HE21, -HE22) side chains derived from relaxation measurements and from the unrestrained and ^3J-coupling time-averaging local elevation restrained MD simulations starting from the 2VB1 X-ray crystal structure. The experimental values for the third set correspond to either HD/E21 or HD/E22 (Buck et al. 1995). The assignment in the second column is based on the best agreement with the values of the MD_2VB1 simulation (third column). Experimental order parameter values larger than 0.95 were set to 0.95 (second column between brackets). Values differing more than 0.2 from the experimental value (0.95 in case the experimental value is larger than 0.95) are denoted using italics.

Residue	Experi-mental value	Unre-strained MD	^3J-coupling local elevation restrained MD			
		2VB1	*2VB1_bb1+bb2*	*2VB1_sc1*	*2VB1_sc1+sc2*	*2VB1_bb1+bb2+sc1+sc2*
S^2_{CH}						
Val 2 CG2	0.598	*0.39*	0.45	0.67	0.59	0.63
Leu 8 CD1	0.767	0.58	0.62	*0.44*	*0.36*	*0.55*
Leu 8 CD2	0.803	0.63	*0.59*	*0.40*	*0.37*	*0.59*
Ala 9 CB	1.0 (0.95)	0.93	0.93	0.93	0.91	0.93
Ala 10 CB	0.901	0.91	0.93	0.92	0.90	0.92
Ala 11 CB	0.861	0.91	0.93	0.92	0.90	0.93
Met 12 CE	0.812	*0.33*	*0.56*	*0.60*	*0.33*	*0.54*
Leu 17 CD1	0.630	0.46	0.49	0.80	*0.37*	0.62
Leu 17 CD2	0.632	0.49	0.52	0.82	*0.41*	0.60
Leu 25 CD1	1.0 (0.95)	*0.40*	*0.35*	*0.48*	*0.35*	*0.53*
Leu 25 CD2	0.609	0.42	*0.37*	0.50	*0.34*	0.51
Val 29 CG1	0.871	*0.57*	0.74	0.79	0.78	0.81
Val 29 CG2	0.791	*0.57*	0.73	0.78	0.77	0.81
Ala 31 CB	0.98 (0.95)	0.94	0.94	0.94	0.93	0.93
Thr 43 CG2	0.361	*0.68*	*0.76*	*0.79*	*0.65*	*0.77*
Thr 47 CG2	0.327	*0.73*	*0.71*	*0.64*	*0.70*	*0.70*
Thr 51 CG2	0.778	*0.49*	*0.53*	0.72	*0.53*	0.71
Ile 55 CG2	0.739	*0.49*	0.78	0.74	0.80	0.62
Ile 55 CD	0.323	*0.55*	*0.54*	0.39	*0.64*	0.44
Leu 56 CD1	0.734	0.79	0.67	0.72	*0.35*	0.69
Leu 56 CD2	0.681	0.75	0.64	0.68	*0.38*	0.63
Ile 58 CG2	1.0 (0.95)	0.84	0.85	*0.69*	0.83	0.83
Ile 58 CD	0.160	*0.81*	*0.77*	*0.75*	*0.77*	*0.79*
Thr 69 CG2	0.98 (0.95)	*0.72*	*0.62*	*0.68*	0.77	0.68
Leu 75 CD1	0.590	0.62	0.49	0.71	0.68	0.69
Ile 78 CG2	0.810	0.85	0.62	0.66	0.75	0.72
Ile 78 CD	0.416	0.43	0.54	0.37	0.40	0.47
Leu 83 CD1	0.884	*0.68*	0.71	*0.26*	*0.58*	*0.61*
Leu 83 CD2	0.783	0.66	0.68	*0.26*	*0.52*	*0.57*
Leu 84 CD1	1.0 (0.95)	*0.46*	*0.62*	*0.68*	*0.40*	*0.56*
Leu 84 CD2	0.879	*0.45*	*0.60*	*0.65*	*0.38*	*0.54*

Ile 88 CG2	0.697	0.55	0.87	0.76	0.72	0.66
Ile 88 CD	0.722	*0.27*	*0.49*	*0.44*	*0.41*	*0.32*
Thr 89 CG2	1.0 (0.95)	*0.71*	*0.64*	*0.69*	*0.62*	*0.61*
Ala 90 CB	0.919	0.91	0.93	0.92	0.92	0.91
Val 92 CG1	0.764	0.63	0.82	0.77	0.68	0.63
Val 92 CG2	0.707	0.61	0.79	0.76	0.68	0.62
Ala 95 CB	0.680	*0.94*	*0.94*	*0.94*	*0.93*	*0.93*
Ile 98 CG2	0.740	0.90	0.82	0.86	0.78	0.83
Ile 98 CD	0.815	0.89	0.63	0.85	0.74	0.86
Val 99 CG1	0.487	*0.85*	0.68	0.48	0.51	0.52
Val 99 CG2	0.517	*0.85*	0.69	0.47	0.52	0.53
Met 105 CE	0.630	0.80	0.46	*0.35*	0.46	0.48
Ala 107 CB	0.832	0.88	0.80	0.77	0.71	0.79
Val 109 CG2	0.354	0.36	0.37	*0.13*	0.20	0.16
Val 120 CG1	0.660	0.69	0.60	0.50	*0.40*	0.56
Ala 122 CB	0.879	0.78	0.83	0.84	0.82	0.83
Ile 124 CG2	0.753	0.75	0.74	0.67	0.61	0.75
Ile 124 CD	0.351	0.48	0.41	0.42	0.36	0.54
Leu 129 CD1	0.525	*0.12*	*0.21*	*0.25*	*0.10*	*0.13*
Leu 129 CD2	0.507	*0.11*	*0.16*	*0.22*	*0.06*	*0.11*
S^2_{NH}						
Trp 28	0.90	0.88	0.86	0.89	0.83	0.82
Trp 62	0.41	*0.73*	*0.67*	*0.65*	*0.71*	*0.72*
Trp 63	0.88	0.83	0.79	0.82	0.84	0.81
Trp 108	0.87	0.87	0.79	0.67	*0.66*	0.70
Trp 111	0.88	0.83	0.76	0.77	*0.65*	0.78
Trp 123	0.85	0.70	0.69	0.71	*0.61*	0.68
Arg 61	0.28	0.22	0.22	0.27	0.22	0.18
Arg 73	0.12	0.24	0.14	0.18	0.19	0.20
Arg 112	0.31	0.28	0.19	0.22	0.11	0.20
Arg 114	0.27	0.13	0.10	0.19	0.14	0.12
Arg 125	0.05	0.12	0.10	0.18	0.10	0.09
S^2_{NH}						
Asn 19 HD21	0.43	0.49	0.44	0.52	*0.13*	0.23
Asn 19 HD22		0.24	0.24	0.37	0.15	0.15
Asn 27 HD21		0.86	0.79	0.76	0.74	0.81
Asn 27 HD22	0.72	0.82	0.52	0.53	0.60	0.54
Asn 37 HD21	0.51	0.37	0.31	0.35	0.53	0.45
Asn 37 HD22		0.21	0.17	0.17	0.30	0.23
Asn 39 HD21	0.74	0.80	0.79	0.69	0.67	0.74
Asn 39 HD22		0.61	0.60	0.60	0.52	0.63
Gln 41 HE21		0.31	0.41	0.41	0.63	0.43
Gln 41 HE22	0.19	0.21	0.22	0.25	0.25	0.21
Asn 44 HD21		0.75	0.67	0.66	0.58	0.79

Asn 44 HD22	0.51	0.71	0.70	0.59	0.46	0.71
Asn 46 HD21		0.85	0.81	0.74	0.80	0.83
Asn 46 HD22	0.62	0.82	0.65	0.51	0.54	0.59
Gln 57 HE21	0.82	0.79	0.74	*0.59*	0.74	*0.59*
Gln 57 HE22		0.76	0.51	0.37	0.66	0.45
Asn 59 HD21		0.92	0.92	0.79	0.81	0.84
Asn 59 HD22	0.78	0.90	0.89	0.71	0.64	0.80
Asn 65 HD21		0.76	0.71	0.77	0.73	0.69
Asn 65 HD22	0.57	0.42	0.34	0.37	*0.28*	*0.28*
Asn 74 HD21	0.74	0.66	0.77	0.65	0.67	0.64
Asn 74 HD22		0.41	0.67	0.40	0.26	0.25
Asn 77 HD21		0.54	0.53	0.57	0.17	0.22
Asn 77 HD22	0.24	0.31	0.35	0.31	0.16	0.18
Asn 93 HD21	0.59	0.53	0.62	0.56	0.79	0.78
Asn 93 HD22		0.34	0.32	0.34	0.48	0.45
Asn 103 HD21		0.72	0.30	0.35	0.33	0.43
Asn 103 HD22	0.26	*0.61*	0.18	0.27	0.28	0.33
Asn 106 HD21	0.58	0.68	0.44	0.58	0.58	0.55
Asn 106 HD22		0.46	0.25	0.31	0.23	0.24
Asn 113 HD21	0.47	0.40	0.61	0.55	0.42	0.59
Asn 113 HD22		0.21	0.25	0.25	0.20	0.25
Gln 121 HE21	0.36	0.34	0.37	0.33	0.34	0.33
Gln 121 HE22		0.18	0.13	0.11	0.13	0.11

Table S3: Backbone S^2_{NH} order parameters derived from relaxation measurements (Buck et al. 1995), from four unrestrained MD simulations starting from four X-ray crystal structures, and from four ^3J-coupling time-averaging local elevation restrained MD simulations starting from the 2VB1 X-ray crystal structure. Experimental order parameter values larger than 0.95 were set to 0.95 (second column between brackets). Values differing more than 0.2 from the experimental value (0.95 in case the experimental value is larger than 0.95) are denoted using italics.

		Unrestrained MD				^3J-coupling local-elevation restrained MD			
Residue	Experi-mental value	*2VB1*	*4LZT*	*1IEE*	*1AKI*	*2VB1_bb1 +bb2*	*2VB1_* sc1	*2VB1_* sc1+sc2	*2VB1_bb1+ bb2+sc1+sc2*
2	0.83	0.73	0.75	0.73	0.73	0.76	0.75	0.67	0.79
3	0.83	0.81	0.81	0.84	0.82	0.77	0.81	0.82	0.83
4	0.83	0.66	0.79	0.78	0.77	0.76	0.76	0.74	0.72
5	0.85	0.76	0.80	0.72	0.77	0.82	0.83	0.76	0.79
6	0.86	0.83	0.83	0.83	0.84	0.83	0.83	0.84	0.84
7	0.88	0.83	0.85	0.85	0.85	0.84	0.85	0.84	0.84
8	0.89	0.89	0.90	0.89	0.90	0.87	0.87	0.88	0.89
9	0.93	0.89	0.89	0.88	0.89	0.88	0.89	0.89	0.89
10	0.89	0.89	0.89	0.89	0.90	0.88	0.89	0.89	0.89
11	0.89	0.89	0.89	0.89	0.89	0.88	0.87	0.88	0.87
12	0.91	0.89	0.90	0.90	0.89	0.89	0.89	0.89	0.89
13	0.92	0.89	0.89	0.88	0.89	0.89	0.89	0.87	0.89
14	0.82	0.89	0.88	0.88	0.89	0.89	0.87	0.85	0.88
15	0.84	0.82	0.80	0.79	0.83	0.81	0.78	0.81	0.80
17	0.89	*0.53*	*0.61*	*0.57*	*0.68*	*0.57*	*0.51*	*0.64*	0.71
18	0.86	*0.55*	*0.58*	*0.46*	0.66	*0.48*	0.75	0.73	*0.63*
19	0.84	0.71	0.64	0.69	0.75	0.67	0.74	0.68	0.71
20	0.85	*0.46*	*0.55*	*0.43*	*0.50*	*0.48*	0.65	*0.58*	*0.54*
21	0.89	0.72	*0.60*	0.70	0.77	0.72	0.82	0.77	0.78
22	0.99 (0.95)	*0.71*	*0.32*	*0.63*	*0.70*	*0.59*	*0.73*	*0.69*	*0.69*
23	0.88	*0.57*	*0.40*	*0.59*	*0.65*	*0.57*	*0.59*	*0.63*	0.72
24	0.89	*0.68*	0.71	*0.65*	0.79	*0.62*	*0.67*	*0.67*	*0.58*
25	0.87	0.73	0.83	0.83	0.85	0.81	0.87	0.85	0.84
26	0.91	0.85	0.85	0.86	0.87	0.84	0.85	0.85	0.86
27	0.94	0.84	0.81	0.84	0.84	0.83	0.81	0.83	0.84
28	0.87	0.86	0.86	0.85	0.86	0.85	0.84	0.87	0.86
29	0.90	0.90	0.89	0.89	0.89	0.89	0.89	0.88	0.88
31	0.93	0.90	0.91	0.90	0.91	0.90	0.90	0.89	0.90
32	0.94	0.81	0.88	0.85	0.88	0.89	0.90	0.85	0.89
33	0.91	0.88	0.87	0.88	0.85	0.89	0.88	0.86	0.88
34	0.92	0.84	0.88	0.85	0.73	0.84	0.84	0.82	0.82
35	0.88	0.83	0.87	0.82	0.74	0.85	0.84	0.84	0.83
36	0.86	0.73	*0.65*	0.76	0.83	*0.65*	0.68	0.67	0.74
37	0.96 (0.95)	0.76	*0.62*	0.74	0.76	*0.58*	*0.60*	*0.74*	0.76

38	0.90	0.85	0.87	0.84	0.89	0.78	0.70	0.82	0.85
39	0.89	0.72	0.71	0.73	0.76	0.73	0.74	*0.66*	0.71
40	0.91	0.85	0.85	0.85	0.85	0.84	0.84	0.80	0.85
41	0.86	0.82	0.81	0.79	0.79	0.81	0.80	0.67	0.82
42	0.87	0.72	*0.64*	0.73	0.71	*0.63*	*0.55*	0.73	0.72
43	0.83	*0.61*	*0.62*	0.65	0.68	0.64	0.63	0.65	*0.59*
44	0.83	0.67	0.66	0.64	0.65	0.68	0.69	*0.50*	0.73
45	0.78	0.71	0.67	0.69	0.66	0.66	0.62	*0.59*	0.65
46	0.83	0.75	0.74	0.73	0.73	0.72	*0.59*	0.74	0.71
47	0.78	0.80	0.75	0.74	0.73	0.74	0.62	0.70	0.73
48	0.77	0.79	0.76	0.75	0.72	0.74	0.57	0.72	0.74
49	0.82	0.72	0.70	0.67	0.62	0.64	*0.52*	0.67	*0.61*
51	0.89	*0.66*	0.77	0.79	0.70	0.78	*0.64*	0.69	0.74
52	0.89	0.83	0.84	0.85	0.83	0.85	0.83	0.82	0.86
53	0.87	0.84	0.83	0.87	0.82	0.83	0.82	0.81	0.86
54	0.91	0.86	0.83	0.84	0.86	0.83	0.84	0.85	0.82
55	0.94	0.84	0.82	0.80	0.83	0.79	0.84	0.83	0.84
56	0.92	0.89	0.88	0.88	0.88	0.86	0.86	0.78	0.89
57	0.94	0.86	0.85	0.88	0.85	0.79	0.78	0.81	0.83
58	0.90	0.86	0.83	0.87	0.84	0.83	0.79	0.83	0.79
59	0.91	0.86	0.85	0.86	0.81	0.79	0.83	0.81	0.83
60	0.93	0.87	0.87	0.87	0.85	0.86	0.85	0.85	0.87
61	0.95	0.77	0.78	0.78	0.76	0.81	0.76	*0.73*	*0.69*
62	0.85	0.85	0.81	0.84	0.76	0.72	0.82	0.84	0.85
63	0.90	0.74	0.77	0.73	0.77	0.80	0.79	0.82	0.78
64	0.91	0.81	0.77	0.80	0.77	0.83	0.73	0.74	0.77
65	0.86	0.84	0.85	0.81	0.81	0.84	0.81	0.82	0.83
66	0.89	*0.61*	*0.58*	*0.52*	*0.68*	*0.64*	0.70	0.69	0.70
67	0.85	*0.56*	0.65	0.60	*0.63*	*0.64*	0.69	0.66	0.68
68	0.78	0.63	0.67	0.66	0.67	0.66	0.59	*0.53*	0.67
69	0.76	0.78	0.81	0.80	0.79	0.71	0.78	0.80	0.71
71	0.72	0.62	0.62	0.65	0.67	0.55	0.68	0.67	0.67
72	0.76	0.62	0.60	0.64	0.68	*0.44*	0.68	0.66	0.65
73	0.88	0.79	0.85	0.82	0.71	0.77	0.75	0.78	0.75
74	0.87	0.84	0.81	0.81	0.68	0.81	0.84	0.82	0.82
75	0.94	0.76	0.84	*0.71*	0.77	0.87	0.83	0.85	0.82
76	0.92	0.86	0.87	0.85	0.87	0.87	0.86	0.87	0.86
77	0.90	0.86	0.87	0.83	0.80	0.84	0.85	0.86	0.86
78	0.91	0.84	0.77	0.73	*0.52*	*0.65*	*0.70*	0.85	*0.52*
80	0.91	0.86	0.87	0.87	0.88	0.87	0.86	0.87	0.85
81	0.86	0.86	0.85	0.87	0.86	0.86	0.85	0.86	0.86
82	0.88	0.81	0.82	0.83	0.83	0.82	0.81	0.80	0.82
83	0.83	0.83	0.85	0.84	0.84	0.86	0.84	0.83	0.84
84	0.83	0.81	0.80	0.82	0.79	0.81	0.80	0.84	0.81
85	0.55	0.45	0.56	0.37	0.45	0.36	0.50	0.71	0.41
86	0.80	0.75	0.75	0.76	0.72	0.75	*0.59*	0.70	0.73

87	0.80	*0.36*	*0.57*	*0.42*	*0.41*	0.60	*0.48*	*0.55*	*0.45*
88	0.80	0.64	0.73	0.69	0.68	0.74	0.72	0.71	0.69
89	0.92	0.81	0.81	0.80	0.81	0.81	0.81	0.81	0.79
90	0.91	0.85	0.85	0.85	0.85	0.84	0.86	0.89	0.86
91	0.85	0.85	0.86	0.84	0.86	0.87	0.87	0.86	0.85
92	0.93	0.88	0.88	0.88	0.89	0.88	0.89	0.88	0.87
93	0.93	0.88	0.87	0.88	0.88	0.88	0.89	0.88	0.88
94	0.92	0.90	0.89	0.89	0.89	0.90	0.90	0.88	0.87
95	0.92	0.91	0.90	0.91	0.90	0.90	0.91	0.89	0.89
96	0.92	0.90	0.87	0.90	0.88	0.88	0.89	0.88	0.88
97	0.94	0.85	0.82	0.84	0.85	0.82	0.84	0.84	0.83
98	0.92	0.88	0.86	0.87	0.86	0.86	0.86	0.85	0.85
100	0.89	0.82	0.62	0.74	0.70	*0.66*	0.76	*0.63*	0.70
101	0.85	0.74	*0.60*	*0.62*	0.66	*0.56*	0.66	*0.59*	*0.59*
102	0.72	0.75	0.70	0.65	*0.45*	0.61	*0.49*	*0.33*	*0.44*
103	0.52	*0.78*	0.68	0.66	0.45	0.58	0.60	0.44	0.50
104	0.81	0.76	0.80	0.63	*0.39*	*0.51*	*0.53*	*0.50*	*0.47*
105	0.88	0.84	0.79	0.75	0.72	*0.62*	0.71	*0.60*	0.68
106	0.96 (0.95)	0.82	0.78	0.79	*0.69*	*0.74*	0.75	*0.70*	*0.69*
107	0.91	0.83	0.80	0.83	0.78	0.77	0.73	*0.60*	0.75
108	0.84	0.84	0.78	0.83	0.80	0.73	0.74	0.65	0.68
109	0.85	0.83	0.80	0.83	0.80	0.76	0.76	*0.63*	0.75
111	0.84	0.82	0.80	0.84	0.81	0.80	0.80	*0.59*	0.79
112	0.89	0.82	0.86	0.76	0.85	0.83	0.83	0.72	0.81
113	0.89	0.76	0.75	0.81	0.83	0.81	0.72	*0.65*	0.77
114	0.87	*0.63*	0.73	0.74	*0.63*	0.76	0.74	*0.63*	0.75
115	0.79	0.72	0.81	0.81	0.77	0.75	0.75	0.73	0.77
116	0.84	*0.58*	*0.59*	*0.59*	0.73	*0.51*	*0.50*	*0.55*	*0.63*
117	0.81	*0.55*	*0.54*	*0.53*	0.69	*0.55*	*0.46*	*0.48*	*0.57*
118	0.72	0.60	0.43	0.61	0.66	*0.44*	*0.44*	*0.41*	*0.47*
119	0.80	0.65	0.62	0.66	0.70	*0.59*	*0.58*	*0.52*	0.65
120	0.80	0.67	0.70	0.67	0.72	0.70	0.71	0.69	0.70
121	0.91	0.76	0.78	0.77	0.81	0.76	0.79	0.77	0.77
122	0.92	0.74	0.83	0.74	0.79	0.79	0.80	0.74	0.82
123	0.90	0.70	0.75	*0.67*	0.72	0.75	0.79	0.76	0.74
124	0.90	0.78	0.77	0.75	0.77	0.77	0.80	0.77	0.78
125	0.87	*0.60*	0.73	*0.51*	*0.65*	0.72	*0.59*	*0.62*	0.68
126	0.82	*0.59*	*0.52*	*0.47*	*0.46*	*0.57*	*0.53*	*0.53*	*0.55*
127	0.77	0.54	0.58	0.39	0.57	0.61	0.62	*0.48*	*0.49*
128	0.76	*0.45*	*0.41*	*0.53*	*0.53*	*0.51*	0.63	*0.45*	*0.43*
129	0.60	*0.25*	*0.29*	0.48	*0.37*	0.40	*0.36*	*0.21*	*0.25*

Figure S1: Secondary structure elements as a function of time calculated for the unrestrained MD simulation *MD_2VB1* starting from the *2VB1* X-ray structure. Red: α-helix; green: π-helix; black: 3_{10}-helix; blue: ß-strand; yellow: ß-bridge; brown: bend; grey: turn.

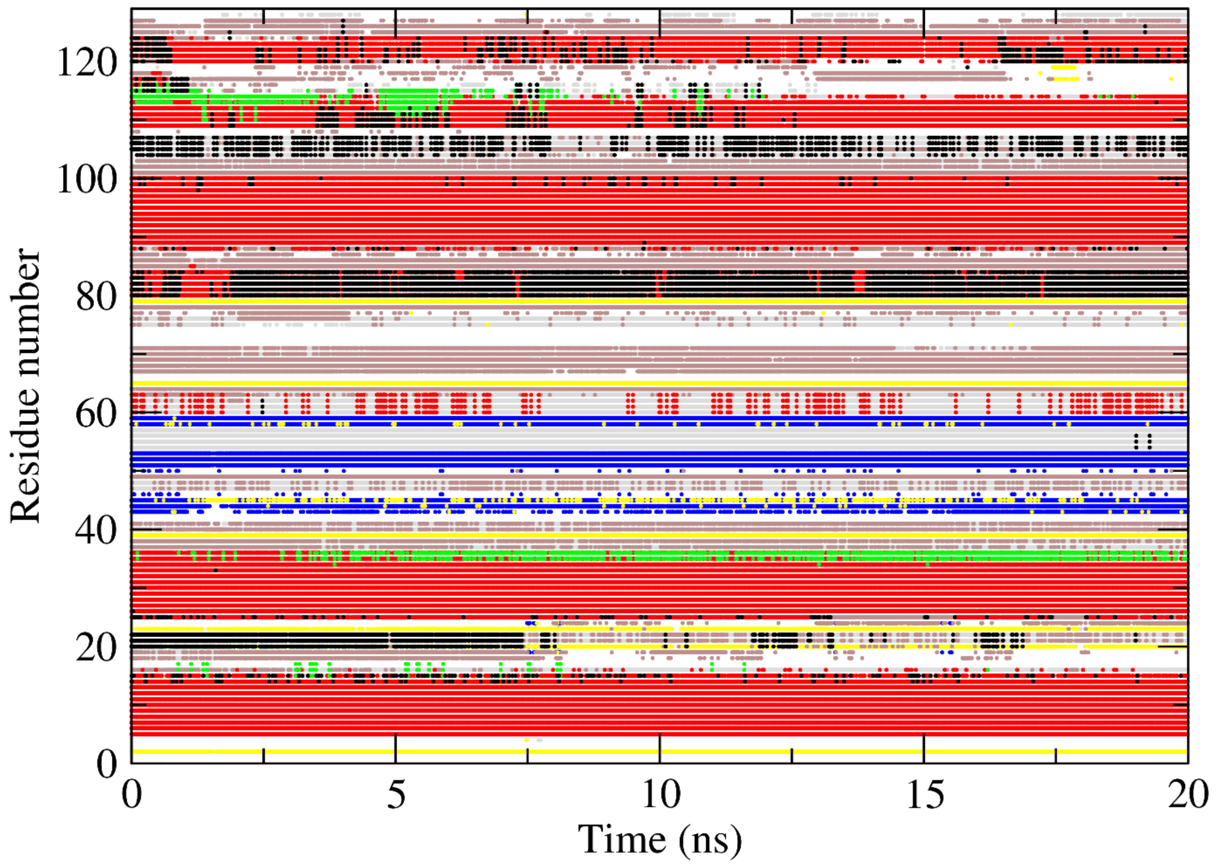

Figure S2: Secondary structure elements as a function of time calculated for the backbone restrained MD simulation *MD_2VB1_bb1+bb2* starting from the *2VB1* X-ray structure. Red: α-helix; green: π-helix; black: 3_{10}-helix; blue: ß-strand; yellow: ß-bridge; brown: bend; grey: turn.

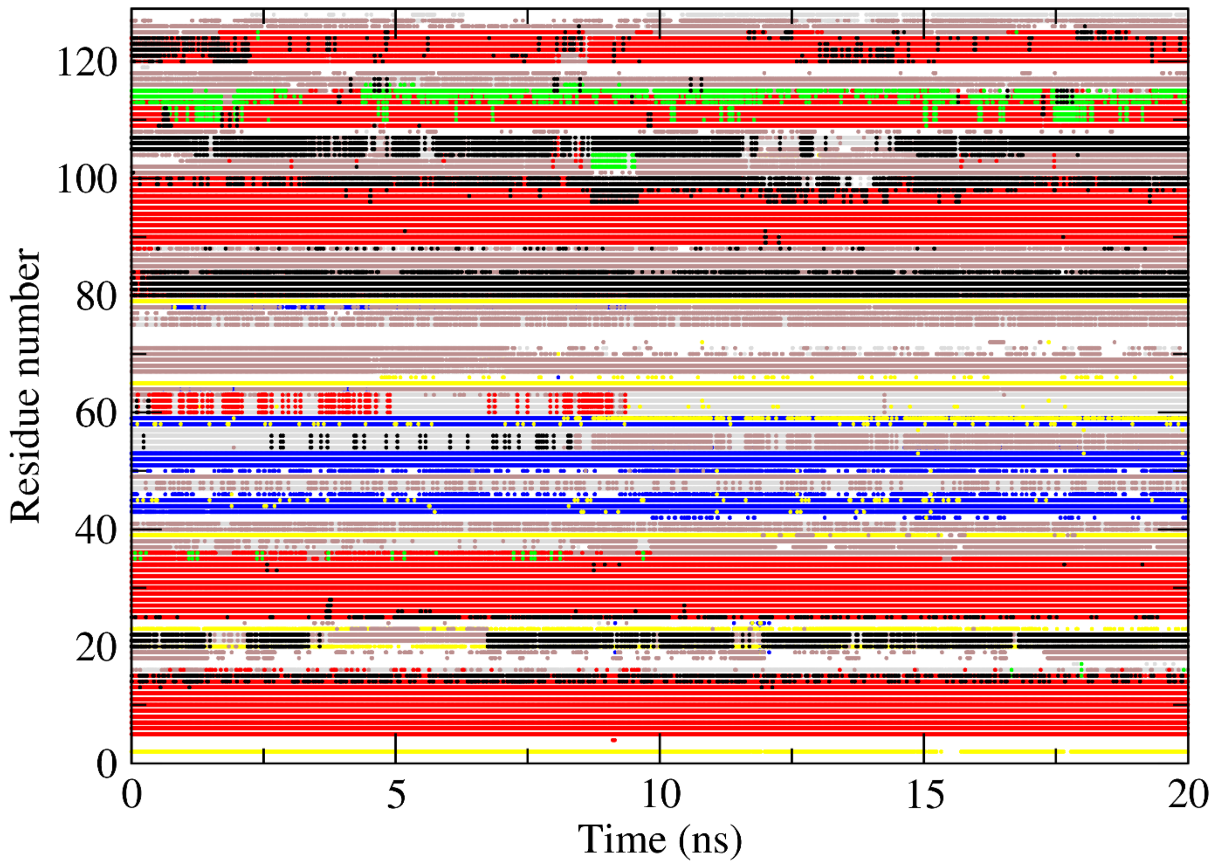

Figure S3: Backbone Cα atom-positional root-mean-square fluctuations (RMSF) as function of residue sequence number for the unrestrained MD simulation *MD_2VB1* (black) and for the four ^3J-coupling restraining MD simulations *MD_2VB1_bb1+bb2* (magenta), *MD_2VB1_sc1* (cyan), *MD_2VB1_sc1+sc2* (orange), and *MD_2VB1_bb1+bb2+sc1+sc2* (blue) all starting from the *2VB1* X-ray structure. The trajectory structures are translationally and rotationally superimposed using the backbone atoms (N, Cα, C) of residues 3 – 126. The black bars at the top indicate secondary structure elements of HEWL (thick bars: α-helix; thin bars, ß-strand).

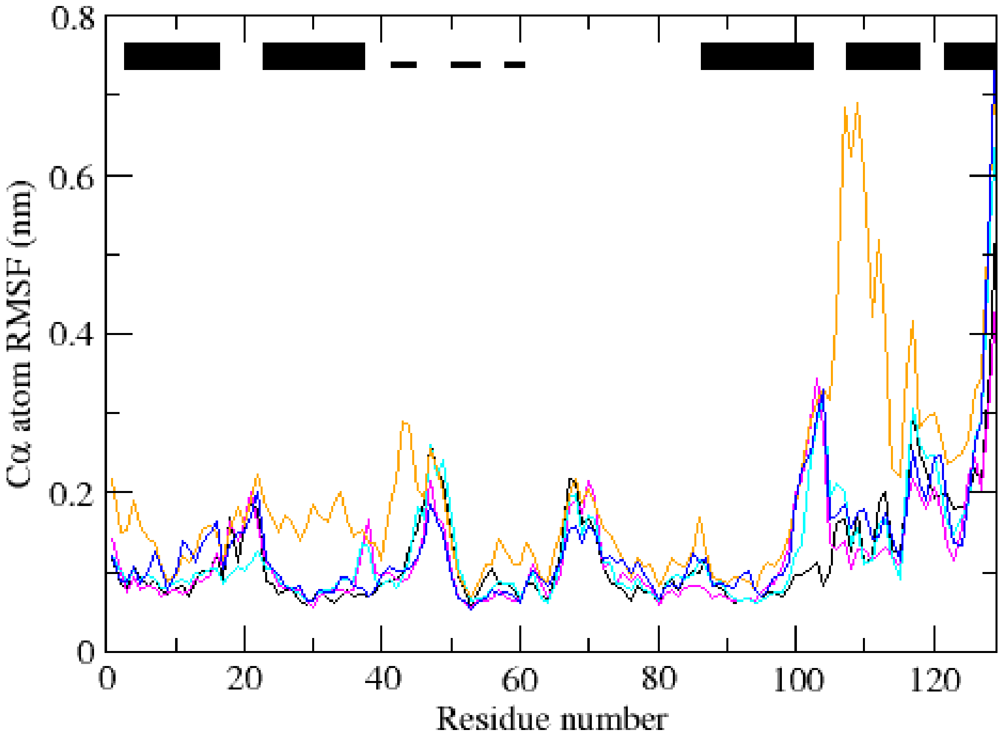

Supplement: Supplementary file 1 — Electronic supplementary material 1 (PDF 12901635 kb) [file 10858_2020_355_MOESM1_ESM.pdf]
